# Pleiotropy and disease interactors: the dual nature of genes linking ageing and ageing-related diseases

**DOI:** 10.1007/s10522-026-10429-w

**Published:** 2026-04-29

**Authors:** Gustavo Daniel Vega Magdaleno, João Pedro de Magalhães

**Affiliations:** 1 Integrative Genomics of Ageing Group, Institute of Life Course and Medical Sciences, University of Liverpool, Liverpool, L78TX UK; 2 Genomics of Ageing and Rejuvenation Lab, Department of Inflammation and Ageing, College of Medicine and Health, University of Birmingham, Birmingham, B152WB UK

**Keywords:** Coexpression, Tissue specificity, Multimorbidity, Longevity

## Abstract

Ageing-related diseases (ARDs) display diverse phenotypes yet share an age-dependent rise in incidence, suggesting mechanistic links with ageing processes. We examined whether ageing-related genes differ systematically from genes associated with multiple ARD clusters. Across 57 ARDs from UK Biobank, network analyses showed that ageing-related genes, although rarely ARD-associated, lie significantly closer to many ARDs through greater-than-chance proximity in protein–protein interaction (PPI) and KEGG networks. Consistent with this network overlap, ageing-related genes tend to occupy intermediate tiers in KEGG signalling cascades and exhibit strong coexpression with disease-associated genes as well as low tissue specificity, supporting that they play regulatory roles across multiple tissues. In contrast, genes associated with multiple ARDs are enriched for immune disorders and tend to have fewer ARD-associated neighbors in PPI networks. Accordingly, genes associated with multiple ARDs also tend to be located in terminal branches of KEGG pathways and show high tissue specificity coupled with weak coexpression with other ARD genes. Lastly, machine learning integration based on gene–disease network topology identified candidate ageing-related genes enriched for intracellular signal transduction and programmed cell death. Altogether, this work reveals two genetic architectures of multi-ARD influence: a cross-tissue regulatory mechanism enriched in ageing-related genes, and a tissue-specific, immune-driven mechanism among pleiotropic disease-related genes.

**Supplementary Information:**

The online version contains supplementary material available at 10.1007/s10522-026-10429-w.

## Introduction

Ageing-related diseases (ARDs) such as stroke, cancer, diabetes and dementia are commonly studied as separate entities, yet their incidence rises sharply with age, pointing to shared mechanisms rooted in the ageing process. Over the past decade, multiple processes (*e.g.*, telomere attrition, cellular senescence, DNA damage, epigenetic alterations, mitochondrial dysfunction and impaired autophagy) have been implicated not only in organismal ageing but also in the initiation and progression of age-associated pathologies (Horvath & Raj 2018; Gruber et al. 2021; Keshavarz et al. 2023; de Magalhães, 2024a). Complementary network-based analyses further reinforce this link by demonstrating that ageing- and disease-associated genes co-localise within shared molecular pathways and cross-tissue interaction layers (Fraser et al. 2022).

Earlier work has examined the relationship between ageing biology and disease risk from multiple perspectives, spanning hallmark-based frameworks to network-level analyses. Signalling pathways such as insulin–IGF, mTOR, and FOXO axes have been repeatedly implicated as mediators bridging organismal ageing with ARD risk, linking longevity control nodes to disease progression (Guo et al. 2022). Evolutionarily conserved ageing-related genes occupy central Protein–Protein Interaction (PPI) network positions that interface with disease-associated pathways, implying that mechanisms selected for longevity also shape age-related pathology (Teulière et al. 2023). Cross-species comparisons have further revealed evolutionary patterns in the overlap between longevity and disease-related genes (Fernandes et al. 2016). At the population level, a UK Biobank study classified 116 diseases by age of onset, showing that ARDs with similar incidence trajectories tend to share genetic architecture (Dönertaş et al. 2021). Yet the overlap between ageing- and ARD-associated genes remains limited, underscoring the need for broader integrative frameworks. In line with this, recent network-based expansions of genome-wide association studies (GWAS) associations have delineated multi-disease gene modules spanning processes such as ubiquitination and RNA processing, providing a scalable route to capture shared architecture beyond single-trait analyses (Barrio-Hernandez et al., 2023).

Pleiotropy, the ability of one gene to influence multiple traits, is a central concept in ARDs. Genes such as *APOE* exemplify how one locus can modulate risk across cardiovascular and neurodegenerative disorders (Ismail et al. 2024). However, pleiotropy alone does not necessarily reflect ageing biology, as the fact that a gene is associated with multiple ARDs does not guarantee that it acts through ageing mechanisms rather than disease-specific pathways (Austad, 2018). Moreover, many genes may not be directly associated with ARDs by GWAS but still influence them indirectly through their interactions with ARD-related genes in molecular networks (Barrio-Hernandez et al., 2023). We term these genes *indirect ARD interactors* (ARD-interactors). While potentially important for understanding coordinated disease processes, they have been largely overlooked and rarely compared to pleiotropic genes in a systematic way.

A key open question is whether Pleiotropic disease-related genes differ functionally from non-Pleiotropic genes that influence ARDs through network interactions. Prior studies indicate that Pleiotropic disease-associated genes do not converge on a single biological axis but instead form a functionally heterogeneous class distributed across multiple biological programmes (Barrio-Hernández et al. 2023). By contrast, ageing-related genes repeatedly concentrate within systemic regulatory processes—such as chromatin maintenance, stress adaptation, proteostasis and metabolic sensing — which modulate vulnerability across tissues rather than driving one disease at a time (Li et al. 2024). Yet these two classes have rarely been contrasted within a unified analytical framework.

The specificity of gene expression across tissues provides an additional layer to understand how ageing-related genes influence ARDs. Genes like *FOXO3* display tissue-dependent roles, with distinct activities across organs that shape different aspects of ageing (Morris et al. 2015; Kodani & Nakae 2020). The expression patterns of disease-associated genes alone cannot explain the tissue-specific nature of human diseases. However, genes coexpressed within the same tissue typically occupy the same interactome neighbourhood, while those from different tissues are located in separate network regions (Kitsak et al. 2016). Ageing-related genes are not randomly distributed across tissues but tend to cluster within specific biological systems (i.e., most notably the brain, skeletal muscle, and immune tissues) consistent with GTEx- and interactome-based analyses showing reproducible tissue-specific ageing signatures (Yang et al. 2015).

Here, we present, to our knowledge, the first systematic analysis jointly evaluating pleiotropy and indirect network connectivity to investigate the genetic relationships between ageing and 57 age-related diseases (ARDs) from the UK Biobank. To enable cross-disease comparisons, ARDs were grouped into higher-order functional clusters termed ARD-Clusters (ARCs), and genes associated with multiple ARCs were defined as highly *ARC-Pleiotropic* genes.

We integrated ageing-related genes from GenAge with ARD-associated genes mapped from previous GWAS and embedded them into protein–protein interaction (PPI), KEGG pathway, and coexpression networks. Using distance-based metrics—shortest- and average-path distances to diseases, disease-neighbour counts, and random walk with restart (RWR)—we quantified the proximity between ageing- and disease-associated genes. Permutation tests revealed that ageing-related genes, although rarely ARD-associated, lie significantly closer to multiple ARDs than expected by chance, primarily through indirect network links. By contrast, highly ARC-Pleiotropic genes, despite their broad GWAS associations, displayed fewer ARD-connected neighbours and weaker indirect connectivity. These groups also diverged in higher-order properties: ageing-related genes occupied intermediate layers of the KEGG hierarchy, showed strong coexpression with ARD-related genes, and were broadly expressed across tissues; pleiotropic genes clustered near terminal KEGG branches, exhibited high tissue specificity, and showed weak coexpression. Finally, machine learning integration based on these network-derived features identified candidate ageing-related genes enriched for intracellular signal transduction and programmed cell death. Together, our findings reveal two complementary architectures of multi-ARD influence: a cross-tissue regulatory mechanism enriched in ageing-related genes, and a tissue-specific, immune-driven mechanism among pleiotropic disease-related genes.

## Results

### Ageing vs high Pleiotropy in GWAS associations with diseases

We first examined genetic *Pleiotropy* across clusters of ARDs (ARCs) to determine whether genes influencing multiple ARDs also overlap with ageing biology, using 307 human ageing-related genes from GenAge ($GenAg{e}_{Hum}$) and 1147 human orthologs of ageing-related genes from model organisms in GenAge ($GenAg{e}_{Mod}$) as reference sets of ageing-related genes. To this end, we analysed 2,503 genes GWAS-associated with at least one of 57 UK Biobank ARDs whose incidence rises with age, grouping them into 8 ARCs (*e.g.* Cardiovascular, Musculoskeletal/Trauma, Endocrine/Diabetes, Gastrointestinal/Abdominal, Renal/Urology, Neurology/Eye/Psychiatry, Haematology/Dermatology, and Immunological/Systemic Disorders) to provide a structure for downstream analyses (see Methods “Retrieval of ARDs and Ageing-related Genes”, “SNP-to-Gene Mapping”, “Clustering of ARDs into ARCs”, and Supplementary Fig. 1).

*Pleiotropy* was quantified at both ARD- and ARC-levels using GWAS-derived mappings (see Methods “Identification of Genes with *ARC-Pleiotropy* and *ARC-Interactors*”). At the ARD level, we counted the number of distinct ARDs directly associated with each gene in GWAS. At the ARC level, we extended these mappings by aggregating all ARD associations within each ARC, so that a gene was considered associated with an ARC if it had at least one GWAS association with any ARD belonging to that ARC. Genes associated to 1–3 ARCs were considered low *ARC-Pleiotropy*, whereas genes associated with 4 or more ARCs were classified as high *ARC-Pleiotropy*, reflecting a broader potential impact on age-related pathology.

Under this framework, we found that most disease-associated genes were confined to one ARC (i.e., low *ARC-Pleiotropy*), while highly *ARC-Pleiotropic* genes were rare (128 out of 2,503 genes) and predominantly associated with the immunological systemic disorders ARC (Supplementary Figs. 2 and 3). These immune-related genes accounted for the majority of genes associated to four or more ARCs (Supplementary Fig. 4).

*GenAge*_*Hum*_-exclusive genes are strongly enriched in regulatory and transcriptional metabolic processes while *GenAge*_*mod*_-exclusive genes are mainly linked to cytoplasmic translation, and the intersection set centers on cellular response, signalling, and stress-related pathways (Supplementary Table 1). On the other hand, the high *ARC*-*Pleiotropy* genes were enriched in chromatin organization, epigenetic regulation, and immune-related functions such as mucosal defense, antibacterial response, and T cell signalling (Supplementary Table 2).

**Fig. 1:**
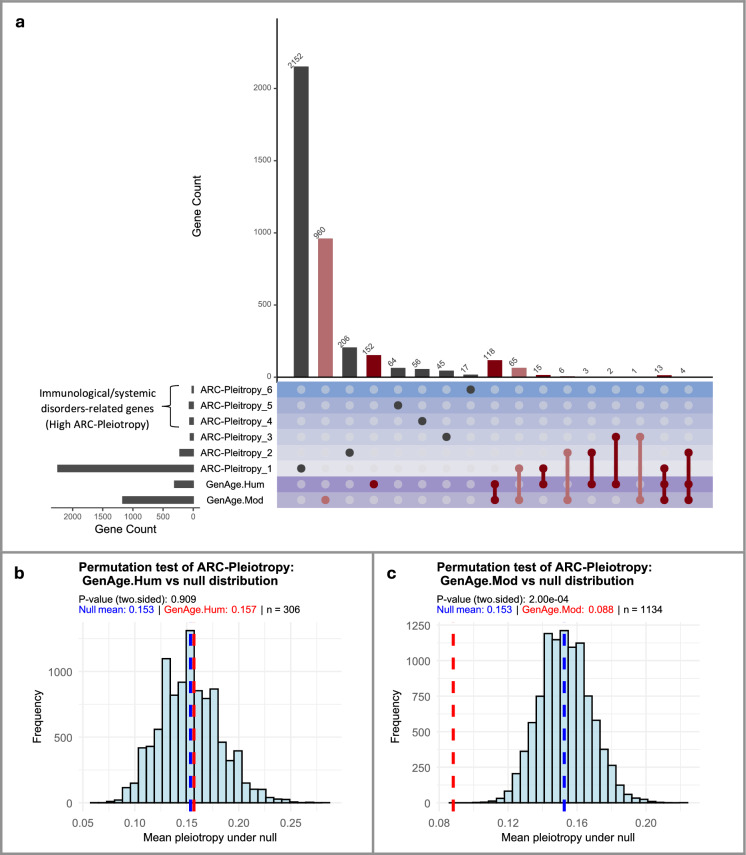
Direct association between ageing-related genes and ARCs. **a**. Overlap between *GenAge* genes and ARC-related genes according to their *ARC-Pleiotropy*, inferred from GWAS associations with individual ARDs and aggregated at the ARC level. Rows represent increasing *ARC-Pleiotropy* levels, i.e., the number of distinct ARCs to which a gene is linked through at least one significant GWAS association with an ARD belonging to that ARC. The numeric suffix in each row label (e.g., *ARC-Pleiotropy1*, *ARC-Pleiotropy2*, etc.) indicates the exact number of ARCs with which the gene is associated. ${GenAge}_{Hum}$ and ${GenAge}_{Mod}$ are highlighted in solid and light red, respectively, while the two purple rows mark the *GenAge* categories. Increasing blue intensities denote higher *ARC-Pleiotrop*y levels. Most GenAge genes did not overlap with any ARC-related gene. Among those that did, the largest overlap occurred at *ARC-Pleiotropy1*, with smaller overlaps at levels 2 and 3, and none beyond level 3. In total, 37 of 306 ${GenAge}_{Hum}$ genes (~ 12.2%) and 89 of 1,134 ${GenAge}_{Mod}$ genes (~ 7.9%) overlapped with ARC-related genes. **b–c**. Permutation tests comparing *ARC-Pleiotropy* of *GenAge* genes against null distributions obtained from 10,000 random samples of protein-coding genes. Histograms show the distribution of mean pleiotropy under the null model. Vertical dashed lines indicate the null mean (blue, 0.153) and the observed mean of the ageing-related gene sets (red). **b**. Human ageing-related genes (${GenAge}_{Hum}$, mean = 0.157; n = 306), showing no significant deviation (*p* = *0.909*). **c.** Model organism ageing-related genes (${GenAge}_{Mod}$, mean = 0.088; n = 1,134), showing a significant reduction relative to the null (*p* = *2.0e-4*). Reported p-values are two-tailed t-test

The direct overlap between ageing-related genes and ARC-associated genes was minimal: only 12.2% of *GenAge*_*Hum*_ genes (37 of 304 genes) and 7.7% of $GenAg{e}_{Mod}$ genes (89 of 1147) overlapped with any ARC gene, and none overlapped with the highly *ARC-Pleiotropic* immune-related set (Fig. 1). Among the overlapping genes, most were associated to a single ARC, with decreasing counts at two or three ARCs.

To evaluate whether this low ARC connectivity was lower than expected by chance, we computed mean *ARC-Pleiotropy*, defined as the expected number of ARCs connected to a gene set via at least one overlapping gene, using random gene sets drawn from the pool of ~ 20,000 protein-coding genes with group sizes matched to $GenAg{e}_{Hum}$ and $GenAg{e}_{Mod}$ (Fig. 1b, c). Permutation analysis showed that $GenAg{e}_{Mod}$ genes exhibited significantly lower *ARC-Pleiotropy* than expected by chance (*observed mean* = *0.08 vs null mean* = *0.153; p* = *2e-4*), whereas $GenAg{e}_{Hum}$ did not deviate from null expectation.

### Ageing vs. high ARC-Pleiotropy in genetic networks

Given the limited direct overlap between ageing-related genes and ARC-related genes — and the lack of overlap with highly *ARC-Pleiotropic* genes, we next examined how both ageing-related genes and highly *ARC-Pleiotropic* genes connect to ARDs through network interactions rather than direct GWAS associations. To this end, we integrated $PPI$, coexpression edges at a 95% threshold ($CO{X}_{95}$) and at a 90% threshold ($CO{X}_{90}$), together with $KEGG$ pathway connectivity, into four ARC-network layers ($PPI$, $CO{X}_{90}$, $CO{X}_{95}$ and $KEGG$), and mapped ageing- and ARD-associated genes onto them (see Methods “Genetic Networks”, Supplementary Tables 3 and 4 and Supplementary Figs. 5–12).

Having established the networks, we next examined whether genes could connect to ARDs through the network even when they were not GWAS-associated themselves. To formalise this, we defined indirect *ARC-Interactions* (*ARC-Interactions*) as cases in which a gene is related to an ARC via at least one network neighbour that is GWAS-associated with a disease in that ARC. This stands in contrast to *ARC-Pleiotropy*, which requires a direct GWAS association to a disease within the ARC (such differences and correlations are further explained in Supplementary Figs. 13–14). Under this definition, a gene that interacts with *n* distinct ARCs is denoted as having *n ARC interactions.*

### Network neighborhood-based associations of genes with ARC-related genes

**Fig. 2:**
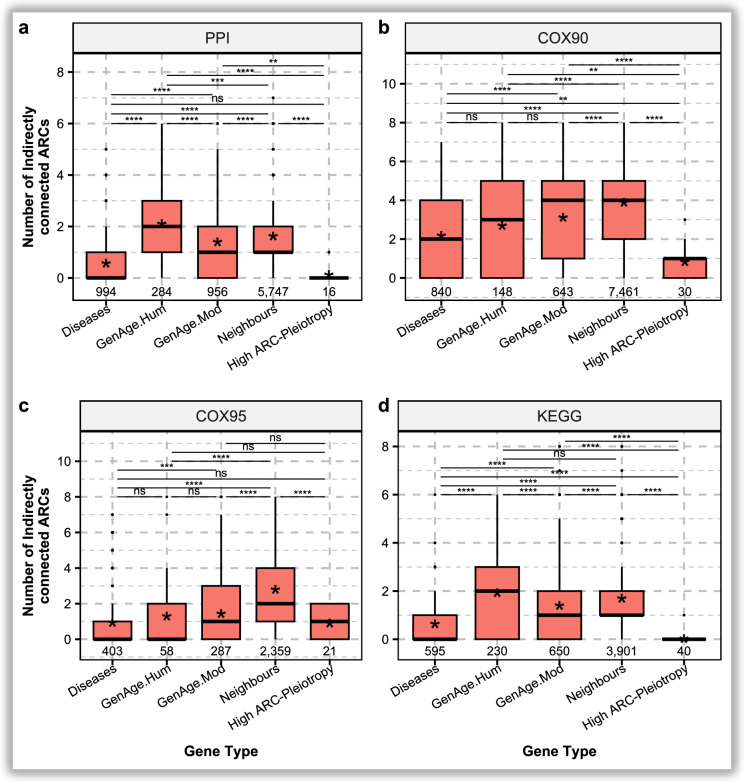
*ARC-Interaction* levels across the following sets of genes: *Diseases*, ${GenAge}_{Hum}$, ${GenAge}_{Mod}$, *Neighbours* of disease-related genes, and high ARC-Pleiotropy genes within each corresponding interaction network. Asterisks represent the mean values to help noticing differences in distributions with equal medians. Boxplots display the median (central line), interquartile range (box; 25th–75th percentiles), and whiskers extending to 1.5×IQR from the quartiles; points beyond this range are shown as outliers. Notably, human ageing-related genes are more extensively studied, which may introduce bias. This same bias is partially inherited by *Neighbours* as their connectivity to disease-associated genes may partly reflect the fact that more extensively studied genes tend to have a greater number of documented interactions. **a**. $PPI$ network. **b.** ${COX}_{95}$ network. **c.** ${COX}_{90}$ network. **d**. $KEGG$ network. Statistical differences (*ns p_adj* > 5e-2, * p_adj ≤ 5e-2, ** padj ≤ 1e-2, *** p_adj ≤ 1e-3, **** p_adj ≤ *1e-4*) were computed using two-sided Wilcoxon rank-sum test and adjusted for multiple tests using Bonferroni correction applied independently within each network, accounting for all pairwise comparisons among the five gene sets (10 tests per network). The Wilcoxon test was selected because *ARC-Interaction* values are discrete counts that follow a strongly right-skewed distribution, with most genes showing low interaction counts; this non-parametric approach is robust to non-normality and differences in sample size across gene sets

Genes were grouped into four categories for the *ARC-Interactor* analysis: Human ageing ${GenAge}_{Hum}$ (human ageing), ${GenAge}_{Mod}$ (Model ageing), $Disease$ (ARDs-associated), high ARC-Pleiotropy genes, and $Neighbours$ (genes adjacent to Disease-related genes in the network). Figure 2 shows the number of ARCs whose genes are reachable via first-order network neighbours in each group, representing the ARCs indirectly reached by genes in that group, and ranging from zero (no ARCs reached) to eight (all ARCs reached). We further tested the enrichment of each gene set using a permutation framework. For each network and gene set, the observed mean number of indirectly reached ARCs was compared against 10,000 size-matched random groups (see Methods “*ARC-Interactions* Analysis”, Table 1 and Supplementary Fig. 15 for statistical values of this analysis).

**Table 1:** Comparison of *ARC-Interactor* counts across networks and gene groups

Network	Group	Observed ARC-Interactions	Null Mean	Null SD	Difference (Observed - Null)	p.value	p_adj
$PPI$	$GenAg{e}_{Hum}$	2.11	0.79	0.07	1.32	1.0e-04	1.6e-03
	$GenAg{e}_{Mod}$	1.40	0.79	0.04	0.61	1.0e-04	1.6e-03
	*Diseases*	0.57	0.79	0.03	− 0.22	1.0e-04	1.6e-03
	*High ARC-Pleiotropic*	0.12	0.78	0.29	− 0.66	1.8e-02	2.9e-01
$KEGG$	$GenAg{e}_{Hum}$	1.93	1.05	0.08	0.88	1.0e-04	1.6e-03
	$GenAg{e}_{Mod}$	1.40	1.05	0.05	0.35	1.0e-04	1.6e-03
	*Diseases*	0.64	1.05	0.05	− 0.41	1.0e-04	1.6e-03
	*High ARC-Pleiotropic*	0.02	1.04	0.19	− 1.02	1.0e-04	1.6e-03
$CO{X}_{90}$	$GenAg{e}_{Hum}$	2.70	2.61	0.21	0.09	6.7e-01	1
	$GenAg{e}_{Mod}$	3.12	2.61	0.10	0.51	1.0e-04	1.6e-03
	*Diseases*	2.18	2.61	0.08	− 0.42	1.0e-04	1.6e-03
	*High ARC-Pleiotropic*	0.83	2.61	0.46	− 1.78	3.0e-04	4.8e-03
$CO{X}_{95}$	$GenAg{e}_{Hum}$	1.29	1.29	0.27	0.00	1	1
	$GenAg{e}_{Mod}$	1.44	1.29	0.12	0.15	2.1e-01	1
	*Diseases*	0.94	1.29	0.10	− 0.35	2.0e-04	3.2e-03
	*High ARC-Pleiotropic*	0.90	1.30	0.45	− 0.39	4.0e-01	1

Observed and expected (null) numbers of *ARC-Interactors* were compared for ageing-, disease-, and high *ARC-Peiotropy*-associated genes across four network layers: $PPI$, $KEGG$, ${COX}_{90}$, and ${COX}_{95}$. The observed value corresponds to the mean number of *ARC-Interactor* connections per gene, while the null represents the mean under 10,000 random permutations. Positive differences indicate higher-than-expected interactivity (enrichment), whereas negative values reflect reduced connectivity relative to random expectation. Adjusted *p*-values (padj) denote significance after Bonferroni correction, computed across all 16 tests corresponding to four gene groups evaluated across four interaction networks

Human ageing-related genes showed the strongest indirect connectivity across ARCs. In $PPI$ they reached 2.11 ARCs vs 0.79 null expected and in $KEGG$ 1.93 vs 1.05 null (both *p_adj* = *1.6e − 3*), nearly doubling random expectation. In coexpression networks their values were similar to random ($CO{X}_{90}$*: 2.70 vs 2.61 null; *$CO{X}_{95}$*: 1.29 vs 1.29 null, ns*). Overall, they exceeded Disease, highly *ARC-Pleiotropic* and Model-ageing-related genes in most contexts. When compared directly against Neighbour genes (Fig. 2a–d), which by definition have high indirect reach due to their adjacency to ARCs, Human ageing-related genes were connected to significantly more ARCs in PPI (2.11 vs ~ 1.62 neighbours; *p_adj* < *1e − 3*), while in KEGG they showed a higher mean without a significant difference (*1.93 vs* ~ *1.69* neighbours). In coexpression networks, this pattern reversed, with Human ageing-related genes connected to significantly fewer ARCs than Neighbours ($CO{X}_{90}$*: 2.70 vs* ~ *3.30* neighbours ;$CO{X}_{95}$*: 1.29 vs* ~ *1.80* neighbours; both p_adj < 1e − 4).

Model-organism ageing-related genes followed the same pattern as Human-ageing-related genes, but with smaller magnitudes. They exceeded random expectation in $PPI$ (*1.40 vs 0.79 null, p_adj* = *1.6e − 3*), in $KEGG$ (*1.93 vs 1.05 null, p_adj* = *1.6e − 3*), and most strongly in $CO{X}_{90}$ (*3.12 vs 2.61 null, p_adj* = *1.6e − 3*), with a more modest excess in $CO{X}_{95}$ (*1.44 vs 1.29 null, ns).* As with Human-ageing-related genes, they outscored Disease and Highly *ARC-Pleiotropic* groups across most networks. However, they were surpassed by Human-ageing-related genes in the interaction-based layers ($PPI$ and $KEGG$), and by *Neighbours* in both coexpression networks, where adjacency to ARD-related genes confers an intrinsic advantage in indirect ARC reach.

Highly *ARC-Pleiotropic* immunological genes showed the opposite architecture. Despite being directly associated with multiple ARDs by GWAS, they did not exceed random expectation in network reach: in $PPI$ they were non significantly below expectation (*0.12 vs 0.78 null, ns*), in $KEGG$ they were substantially below random (*0.02 vs 1.04 null, p_adj* = *1.6e-3*), and in coexpression their values remained below ($CO{X}_{90}$*: 0.83 vs 2.61 null, p_adj* = 4.8-e3) or aligned ($CO{X}_{95}$*: 0.90 vs 1.30 null, ns*) with random expectation. Across pairwise contrasts, they consistently showed lower values than the Ageing and Neighbour groups, and often displayed similar values to the Disease-related genes. Thus, although these genes are broadly high *ARC-Pleiotropic* in GWAS, this does not translate into broad ARC reach through molecular networks, indicating a specialised rather than integrative role.

Disease-related genes, despite being directly associated to ARDs by GWAS, consistently reached fewer ARCs than expected by chance across all networks: 0.57 *vs 0.79 null* in $PPI$ *(p_adj* = *1.6e − 3), 0.64 vs 1.05 null* in $KEGG$* (p_adj* = *1.6e − 3), 2.18 vs 2.61 null* in $CO{X}_{90}$* (p_adj* = *1.6e − 3), and 0.94 vs 1.29 null* in $CO{X}_{95}$ *(p_adj* = *3.2e − 3)*. In pairwise comparisons, their indirect associations were outnumbered by the ${GenAge}_{Mod}$ and *Neighbour* groups in every network, and matched high *ARC-Pleiotropic* genes in $PPI$ and ${COX}_{95}$. This indicates that, although disease-associated, these genes remain relatively isolated from other disease-related genes in their network neighborhood.

### Network shortest path distance and random walk with restart: ageing vs. high ARC-Pleiotropy

To reinforce these findings, we quantified two complementary properties: the shortest-path distance to ARC-related genes, which counts the number of edges separating each gene from them (See Methods “Shortest and average paths to diseases” and Supplementary Fig. 16), and the diffusion probability from ARC-related genes, estimated using the Random Walk with Restart (RWR) algorithm (Tables 2 and 3).

**Table 2:** Summary of *Shortest_Path_Distance*-based metrics evaluating the association of ageing-related, disease-associated, and highly *ARC-Pleiotropic* immunological genes with ARCs across four interaction networks ($PPI$, $KEGG$, $CO{X}_{90}$, $CO{X}_{95}$)

Network	Group	Observed shortest path distance to ARCs	Null Mean	Null SD	Difference (Observed - Null)	p.value	P_adj
$PPI$	${GenAge}_{Hum}$	1.72	2.16	0.04	− 0.44	1.0e-04	1.6e-03
	${GenAge}_{Mod}$	1.93	2.16	0.02	− 0.23	1.0e-04	1.6e-03
	$Diseases$	1.46	2.16	0.02	− 0.70	1.0e-04	1.6e-03
	*High ARC-Pleiotropic*	0.45	2.17	0.16	− 1.72	1.0e-04	1.6e-03
$KEGG$	${GenAge}_{Hum}$	1.89	2.29	0.06	− 0.39	1.0e-04	1.6e-03
	${GenAge}_{Mod}$	2.21	2.28	0.03	− 0.07	3.7e-02	6.0e-01
	$Diseases$	1.44	2.28	0.04	− 0.84	1.0e-04	1.6e-03
	*High ARC-Pleiotropic*	0.44	2.29	0.15	− 1.85	1.0e-04	1.6e-03
${COX}_{90}$	${GenAge}_{Hum}$	1.88	2.26	0.15	− 0.38	1.0e-02	1.7e-01
	${GenAge}_{Mod}$	1.71	2.25	0.07	− 0.54	1.0e-04	1.6e-03
	$Diseases$	1.38	2.25	0.06	− 0.87	1.0e-04	1.6e-03
	*High ARC-Pleiotropic*	0.52	2.28	0.35	− 1.76	7.0e-04	1.1e-02
${COX}_{95}$	${GenAge}_{Hum}$	4.26	4.72	0.70	− 0.46	5.1e-01	1
	${GenAge}_{Mod}$	3.89	4.65	0.29	− 0.75	1.2e-02	1.9e-01
	$Diseases$	2.38	4.64	0.24	− 2.27	1.0e-04	1.6e-03
	*High ARC-Pleiotropic*	0.53	4.86	1.28	− 4.32	9.3e-03	1.5e-01

For each group and network, reported values include observed means, null expectations from permutation tests, adjusted p-values, and observed–null differences. Adjusted p-values (p_adj) were computed using Bonferroni correction across all 16 tests, corresponding to four gene groups evaluated across four interaction networks

**Table 3:** Summary of RWR-based metrics evaluating the association of ageing-related, disease-associated, and highly *ARC-Pleiotropic* immunological genes across the four interaction networks ($PPI$, $KEGG$, $CO{X}_{90}$, $CO{X}_{95}$)

Network	Group	Observed RWR score from ARCs	Null Mean	Null SD	Difference (Observed - Null)	p.value	P_adj
$PPI$	${GenAge}_{Hum}$	8.9e-05	2.4e-05	7.8e-06	6.5e-05	1.0e-04	1.6e-03
	${GenAge}_{Mod}$	4.1e-05	2.4e-05	4.1e-06	1.7e-05	5.0e-04	8.0e-03
	$Diseases$	1.6e-05	2.4e-05	4.1e-06	− 8.1e-06	4.0e-02	6.4e-01
	*High ARC-Pleiotropic*	1.2e-06	2.3e-05	3.2e-05	− 2.2e-05	1.1e-01	1
$KEGG$	${GenAge}_{Hum}$	1.6e-04	4.6e-05	2.2e-05	1.1e-04	1.5e-03	2.4e-02
	${GenAge}_{Mod}$	1.1e-04	4.5e-05	1.2e-05	6.8e-05	1.0e-04	1.6e-03
	$Diseases$	3.0e-05	4.5e-05	1.3e-05	− 1.5e-05	1.8e-01	1
	*High ARC-Pleiotropic*	2.8e-05	4.4e-05	4.7e-05	− 1.6e-05	6.1e-01	1
${COX}_{90}$	${GenAge}_{Hum}$	2.2e-05	2.3e-05	5.8e-06	− 1.1e-06	8.3e-01	1
	${GenAge}_{Mod}$	2.6e-05	2.3e-05	2.7e-06	2.9e-06	2.8e-01	1
	$Diseases$	2.5e-05	2.3e-05	2.4e-06	2.1e-06	3.9e-01	1
	*High ARC-Pleiotropic*	1.2e-04	2.3e-05	1.3e-05	1.0e-04	4.0e-04	6.4e-03
${COX}_{95}$	${GenAge}_{Hum}$	5.0e-05	4.2e-05	2.5e-05	7.7e-06	7.1e-01	1
	${GenAge}_{Mod}$	3.4e-05	4.3e-05	1.1e-05	− 8.5e-06	4.4e-01	1
	$Diseases$	4.8e-05	4.3e-05	9.3e-06	5.5e-06	5.7e-01	1
	*High ARC-Pleiotropic*	2.9e-04	4.3e-05	4.2e-05	2.5e-04	4.9e-03	7.8e-02

For each group and network, reported values include observed means, null expectations from permutation tests, adjusted p-values, and observed–null differences. Adjusted p-values (p_adj) were computed using Bonferroni correction across all 16 tests, corresponding to four gene groups evaluated across four interaction networks

In RWR, a simulated “walker” repeatedly moves through the network starting from ARC-related genes, occasionally returning to them, and records how often each gene is reached during this process. The resulting RWR score represents the steady-state probability that a gene is visited during these random walks, a quantitative measure of how easily it can be reached from ARC-related genes through the network’s overall structure. Genes with higher scores are thus considered more accessible or influentially positioned, even if they are not directly connected (see Methods “Random Walk with Restart” and Supplementary Fig. 17).

### Shortest-path analyses

Shortest-path analyses showed that most gene groups tended to lie closer to ARC-associated genes than expected by chance, although the magnitude of this proximity varied across networks (Table 2). In interaction networks ($PPI$, $KEGG$), human and model ageing-related genes consistently showed shorter distances to ARCs ($PPI$*: 1.72–1.89 vs. 2.16 null; *$KEGG$*: 1.89–2.21 vs.* ~ *2.28 null, p_adj* = *1.6e-3*), except for $GenAg{e}_{Mod}$ in $KEGG$ (*2.21 vs. 2.28 null*, *ns*). Disease-associated genes were even closer (~ *1.45 vs. 2.16–2.28 null, p_adj* = *1.6e-3*), and high *ARC-pleiotropy* genes showed by far the smallest distances (~ *0.45 vs. 2.17–2.29 null, p_adj* = *1.6e-3*).

In ${COX}_{90}$, all gene sets except $GenAg{e}_{Hum}$ showed significantly shorter distances to ARCs (0*.52–1.71 vs.* ~ *2.25 null, p_adj* < *1.1e-*2), with high *ARC-pleiotropy* genes displaying markedly smaller distances than all other groups and the null model (0.52 vs. ~ 2.28 null, *p_adj* = *1.1e-2*). In ${COX}_{95}$, although this strong separation was largely attenuated, high *ARC-pleiotropy* genes still showed much shorter absolute distances than other gene sets and the null (*0.53 vs.* ~ *4.86 null, ns*), but this difference was no longer statistically significant, while only disease-associated genes remained significantly closer to ARCs (*2.38 vs. 4.64 null, p_adj* = *1.6e-3*). Full statistics are reported in Table 2 and Supplementary Fig. 18.

### RWR analysis

RWR analyses revealed a complementary contrast (Table 3). In interaction networks ($PPI$, $KEGG$), $GenAg{e}_{Hum}$ genes showed markedly higher diffusion accessibility from ARCs ($PPI$*: 8.9e-5 vs. 2.4e-5 null, p_adj* = *1.6e-3; *$KEGG$*: 1.6e-4 vs. 4.6e-5 null, p_adj* = *2.4e-2*), with $GenAg{e}_{Mod}$ showing similar increases ($PPI$*: 4.1e-5 vs. 2.4e-5 null, p_adj* = *8e-3; *$KEGG$*: 1.1e-4 vs. 4.5e-5 null, p_adj* = *1.6e-3*), indicating that ageing-related genes preferentially remain within ARC regions during diffusion. Disease-associated genes showed near-null or slightly depleted diffusion in both $PPI$ and $KEGG$. High *ARC-Pleiotropy* genes did not differ significantly from the null model in either PPI or KEGG. In PPI, although their RWR scores were markedly lower than the null expectation (1.2e − 6 vs 2.3e − 5 null, ns) and lower than those of all other gene sets (1.2e − 6 vs 1.6–8.9e − 5), these differences did not reach statistical significance after multiple-testing correction.

This pattern reversed in coexpression networks. In ${COX}_{90}$, high *ARC-pleiotropy* genes showed significantly stronger diffusion from ARCs than expected by chance (*1.2e-4 vs. 2.3e-5 null; p_adj* = *6.4e-3*), whereas ageing- and disease-related gene sets did not differ from the null model. In ${COX}_{95}$ no gene set showed a statistically significant deviation from the null, although high *ARC-pleiotropy* genes displayed higher absolute diffusion values (*2.9e-4 vs. 4.3e-5 null, ns*). Full statistics are provided in Table 3 and Supplementary Fig. 19.

### Coexpression within and across ARCs- and GenAge-related genes

**Fig. 3:**
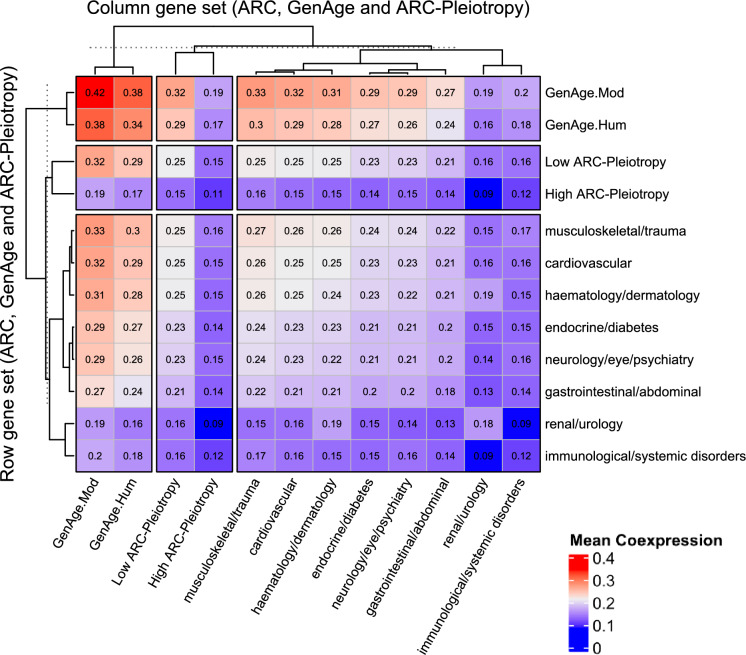
*Intra*- and *Inter-set coexpression* among ARCs, GenAge genes, and different levels of *ARC-Pleiotropy*. Rows and columns are grouped into three main community categories, each further divided into subcolumns representing the corresponding groups. Each cell indicates the average coexpression between the gene-sets of the given row and column. Values along the main diagonal represent *Intra-set coexpression*, as they depict the mean value of the corresponding gene-set with itself, whereas off-diagonal values capture *Inter-set coexpression*. Statistical significance of differences in *Intra-set coexpression* is presented in Supplementary Fig. 20

We examined how strongly genes coexpress within a given biological category and between sets (Fig. 3 for coexpression levels of sets and Supplementary Fig. 20 for statistical tests on differential inter-set coexpression between gene sets). Sets were defined in three ways: (1) by ARCs (*e.g.* Cardiovascular, Endocrine), (2) by ageing-related origin ($GenAg{e}_{Hum}$, $GenAg{e}_{Mod}$), and (3) by degree of *ARC-Pleiotropy* (*e.g.* high vs. low *ARC-Pleiotropy* genes). For each category, *intra-set coexpression* refers to the average absolute correlation between genes belonging to the *same* set (*e.g.* genes within the Cardiovascular ARC, or genes with high *ARC-Pleiotropy*). *Inter-set coexpression* refers to the average correlation between genes from *two different* sets (*e.g.* Cardiovascular vs. Endocrine, or $GenAg{e}_{Hum}$ vs. High *ARC-Pleiotropic* genes). As shown in Fig. 3, diagonal blocks in the heatmap correspond to *intra-set coexpression*, whereas off-diagonal blocks represent *inter-set coexpression* between sets. Full details are provided in Methods “Gene Coexpression Analysis”.

Upon stratification by *ARC-Pleiotropy*, genes with low to moderate *ARC-Pleiotropy* (1–3 ARCs) showed higher *intra*- and *inter-set coexpression* (typically ~ *0.16–0.32*) than those with high *ARC-Pleiotropy* (≥ 4 ARCs), whose coexpression values were consistently weaker (*0.09–0.19*). The highly *ARC-Pleiotropic* set exhibited the lowest inter-set coexpression with ARCs of all gene sets (≈*009–0.16*), matching the low *intra-set coexpression* of the immunological ARC (≈*0.09–0.17*), and these differences were robust to multiple-testing correction. In contrast, ageing-related genes remained the most strongly coexpressed with most gene sets (*0.16–0.42*) yet coexpressed poorly with the highly *ARC-Pleiotropic* set (≈*0.17–0.19*). This pattern paralleled the separation observed between ageing and immunological disorders gene set (≈*0.18–0.2*).

**Fig. 4:**
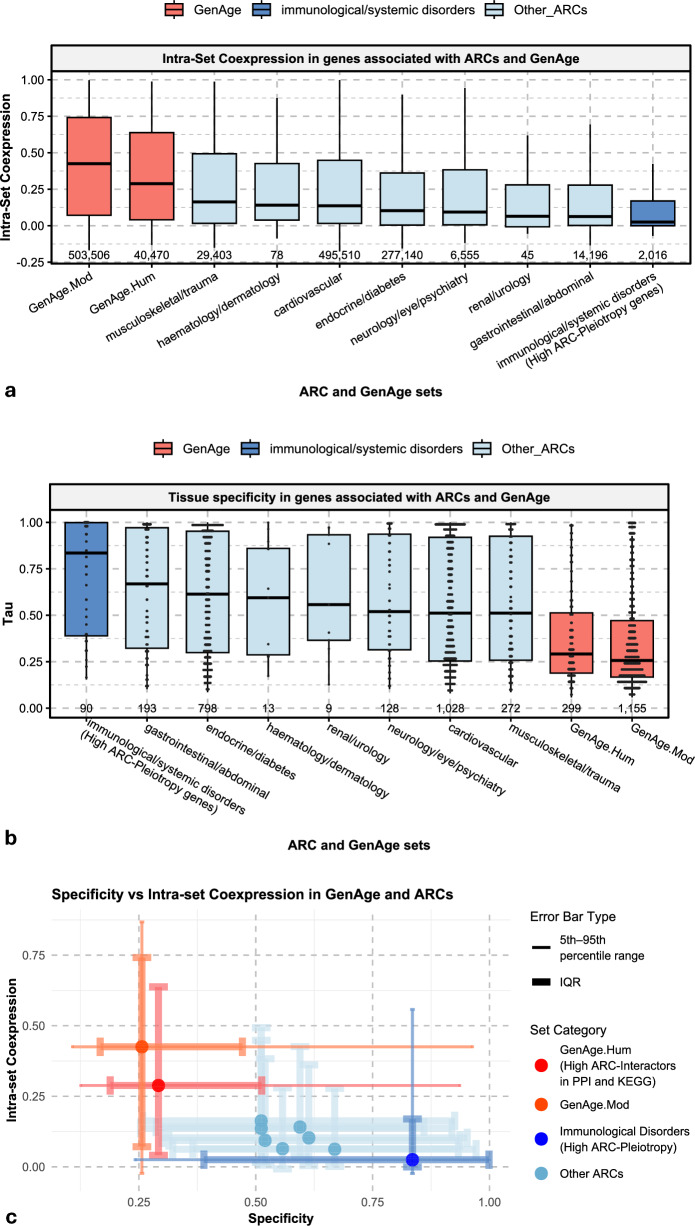
*Intra-set Coexpressio*n and Tissue Specificity (Tau) across ARCs and Ageing. **a**. *Intra-set Coexpression*. **b**. Tissue Specificity. **c**. Tissue Specificity vs *Intra-set Coexpression*. Boxplots display the median (central line), interquartile range (box; 25th–75th percentiles), and whiskers extending to 1.5×IQR from the quartiles; points beyond this range are shown as outliers. In panel (b), individual data points are overlaid. In panel (c), points represent median values, with thick error bars indicating the interquartile range (IQR; 25th–75th percentiles) and thin error bars representing the 5th–95th percentile range. Statistical tests for group differences are depicted in Supplementary Figs. 20 and 21. *GenAge* groups are always significantly different from the other groups, including Immunological systemic disorders. On the other hand, immunological systemic disorders are not always significantly different from other ARCs in terms of Specificity. However, it tends to have a lower mean in terms of *Intra-set coexpression*

Figure 4a further illustrates differences in *intra-set coexpression* between gene groups (see Supplementary Fig. 20 for statistics). Both $GenAg{e}_{Hum}$ and $GenAg{e}_{Mod}$ exhibited significantly stronger *intra-set coexpression* than ARCs (*p_adj* ≤ *1e − 4*), with $GenAg{e}_{Mod}$ showing the highest overall value (*0.42*) and $GenAg{e}_{Hum}$ slightly lower (*0.34*). These intra-set coexpressions were both substantially higher than any ARC- or *ARC-Pleiotropy-*associated gene set (*0.12–0.27; p_adj* ≤ *1e − 4* in most comparisons). Immunological/systemic disorder genes (i.e. ARC-Pleiotropic genes) showed the lowest *intra-set coexpression* overall (*0.12; p_adj* < *1e − 4 vs. most groups*). Together, these results indicate that ageing-related genes form stronger coexpression modules, whereas highly *ARC-Pleiotropic* disease-related genes are transcriptionally dispersed rather than co-regulated.

**Fig. 5:**
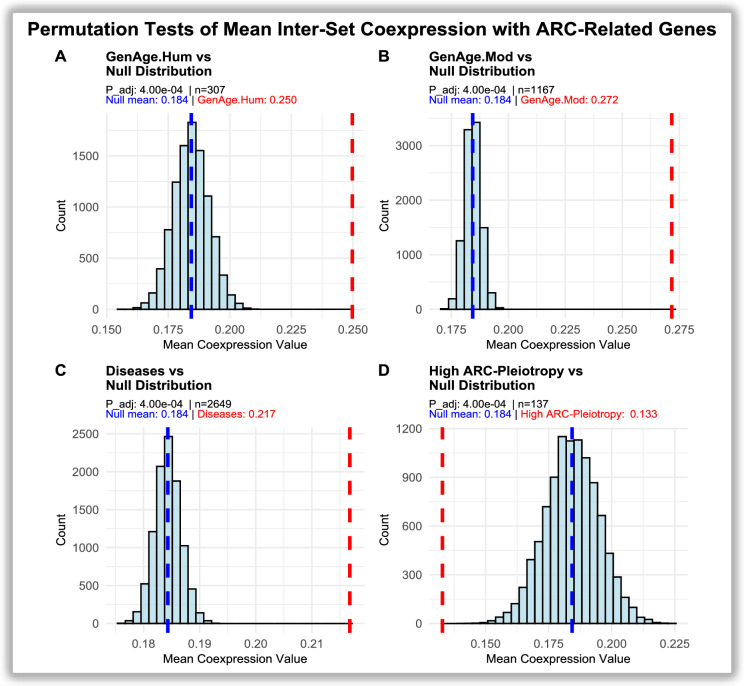
Histograms of null distributions of mean *inter-set coexpression* values with ARC-associated genes obtained from 10,000 permutations of protein-coding genes. Blue dashed lines indicate the mean of the null distribution, while red dashed lines mark the observed values for each gene set (**a**: $GenAg{e}_{Hum}$, **b**: $GenAg{e}_{Mod}$, **c**: Diseases, **d**: high *ARC-pleiotropy* immunological disorder genes). Adjusted p-values (p_adj) were computed using Bonferroni correction across the four gene sets (a–d)

Permutation analysis of *inter-set coexpression* confirmed these trends (Fig. 5). When testing each gene set against 10,000 size-matched randomisations across all eight ARCs, ${GenAge}_{Mod}$ showed the highest *inter-set coexpression* values (*0.272 vs 0.184 null; p_adj* = *4e-4*), followed by ${GenAge}_{Hum}$ (*0.250 vs 0.184 null; p_adj* = *4e-4*), and to a lesser extent *Disease-related genes* (*0.217 vs 0.184 null; p_adj* = *4e-4*). By contrast, the immune/high *ARC-Pleiotropy* group showed a significant depletion (*0.133 vs 0.184 null; p_adj* = *4e-4*), consistent with their low internal and *inter-set coexpression* values in Fig. 4. This confirms that ageing-related genes are systematically more coexpressed across ARCs, whereas high *ARC-Pleiotropic* genes remain transcriptionally isolated.

### Tissue specificity across ageing-related and ARC-associated genes

The degree to which a gene is expressed broadly versus confined to a narrow set of tissues provides an intrinsic readout of its biological scope. To complement the coexpression analyses reported above, we therefore examined tissue specificity across gene groups using the *Tau* coefficient derived from GTEx, where values close to 0 indicate ubiquitous expression and values near 1 indicate high tissue specificity (see Methods “Tissue Specificity Analysis”).

As shown in Fig. 4b and Supplementary Fig. 21, ageing-related genes displayed significantly lower tissue specificity (mean Tau ≈ 0.36–0.38; *p_adj* ≤ 1e-4) than disease-associated genes (*mean Tau ≈ 0.56–0.72*). Among ARCs, immunological genes exhibited the highest mean Tau values (≈ *0.72*), although these differences were not statistically significant compared to most gene sets excepting the Cardiovascular ARC and ageing-related genes.

**Fig. 6:**
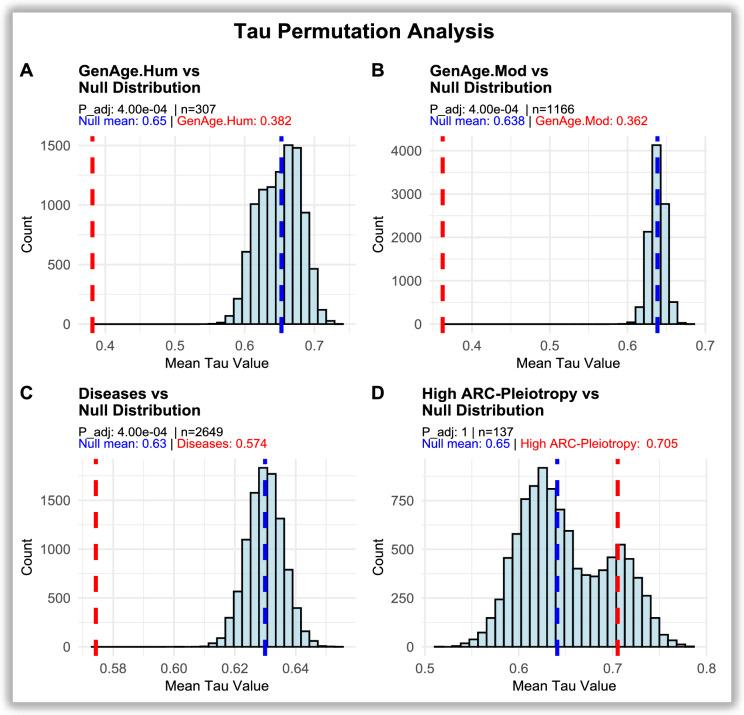
*Permutation tests of differences in mean tissue specificity (Tau).* Histograms represent null distributions of mean *Tau* values generated from 10,000 random gene sets. Red dashed lines indicate the observed mean *Tau* for each group, and blue lines denote the null mean. **a** $GenAg{e}_{Hum}$ (*mean* = *0.382 vs null* = *0.65, p_adj* = *4e-4*). **b** $GenAg{e}_{Mod}$ (*mean* = *0.362 vs null* = *0.638, p_adj* = *4e-4*). **c** Diseases (*mean* = *0.574 vs null* = *0.63, p_adj* = *4e-4*). **d** *High ARC-Pleiotropy* (*mean* = *0.705 vs null* = *0.65, p_adj* = *1*). Ageing-related genes (**a–b**) show broad, multi-tissue expression, while highly *ARC-Pleiotropic* disease-related genes (**d**) display greater tissue specificity but without significant deviation from random expectation. Adjusted p-values (p_adj) were computed using Bonferroni correction across the four gene sets (a–d)

Permutation analysis of tissue specificity was performed using *Tau* specificity values per gene, with permutations carried out across all genes with GTEx records to build null distributions matched by gene-set size (Fig. 6). Ageing-related genes showed significantly *lower* tissue specificity than expected by chance (${GenAge}_{Hum}$ = *0.382 vs 0.65 null;* ${GenAge}_{Mod}$ = *0.362 vs 0.638 null; both p_adj* = *4e-4*), consistent with broad expression across tissues. By contrast, high *ARC-Pleiotropic* genes exhibited elevated *Tau* values that did not differ from the null (*0.705 vs 0.65 null; p_adj* = *1*), indicating that their strong tissue specificity is of the magnitude expected under random sampling from GTEx genes. Disease-related genes showed a mild but significant reduction in specificity relative to the null (0.574 *vs 0.63 null; p_adj* = *4e-4*), consistent with intermediate specificity. These results confirm that ageing-related genes are broadly expressed across tissues, whereas highly *ARC-Pleiotropic* disease-related genes are tissue-specific to a degree that aligns with background transcriptome structure.

Consistent with ageing-related genes showing broad expression, *Tau* values in the $PPI$ network declined with increasing *ARC-Interactivity*, dropping from a median of 0.29 Tau in genes interacting with one ARC to 0.17 in those interacting with four or more (Supplementary Fig. 22). This pattern reflects the predominance of ageing-related, widely expressed genes among highly interactive nodes. In contrast, *Tau* increased with *ARC-Pleiotropy*, remaining near 0.52 for genes associated with one to three *ARC-Pleiotropy* but rising to 0.80 in those associated with four or more, indicating that highly pleiotropic genes are transcriptionally restricted rather than broadly active. In $KEGG$, Tau stayed nearly constant (~ 0.52) across ARC-Pleiotropies, with only a mild decline in the highest group (0.47), confirming the stability of tissue specificity in this network.

Aligned with these trends, GTEx expression distributions show that GenAge genes are broadly and uniformly expressed, whereas immunological genes are highly restricted to immune tissues such as blood and spleen (Supplementary Figs. 23–25).

### Tissue specificity vs coexpression within ARCs

The coexpression profiles in Fig. 4a showed that GenAge genes form the most internally coexpressed sets, whereas immunological disorder genes exhibit the weakest *intra-set coexpression* (Supplementary Fig. 20). When considering tissue specificity in Fig. 4b, GenAge genes have low *Tau* values and immunological genes have high *Tau*.

Plotting *intra-set coexpression* against tissue specificity (Fig. 4c) emphasised the polarity between groups. $GenAg{e}_{Hum}$ genes occupied the region of higher coexpression (≈0.34–0.42) and lower specificity (≈0.36–0.38), and $GenAg{e}_{Mod}$ accentuated this profile with the strongest coexpression and lowest specificity of all groups. In contrast, immunological systemic disorders—defined by high *ARC-Pleiotropy*—clustered in the opposite corner, combining the weakest coexpression (≈0.12) with the highest specificity (≈0.72).

The remaining ARCs — cardiovascular, endocrine/diabetes, gastrointestinal, musculoskeletal, renal/urology, haematology/dermatology, and neurological/psychiatric — occupied intermediate ranges in both *Tau* (≈*0.56–0.63*) and coexpression (≈*0.18–0.27*), without a consistent cross-group pattern. Thus, the strong opposition seen between ageing-related genes and immunological disorders is specific to those two categories and is not recapitulated by the other disease groups.

### Hierarchical positioning of ageing-, disease- and high ARC-Pleiotropy-associated genes within ${\mathrm{K}}{\mathrm{E}}{\mathrm{G}}{\mathrm{G}}$

We analyzed the KEGG pathway hierarchy to determine whether these gene groups preferentially occupy upstream regulatory tiers or downstream functional branches. Specifically, we quantified each gene’s relative distance from both the root (regulatory level) and terminal leaves (functional endpoints) of the KEGG hierarchy and compared these distances against null distributions obtained from 10,000 random gene sets of equal size (see Methods "$KEGG$ hierarchy analysis").

**Fig. 7:**
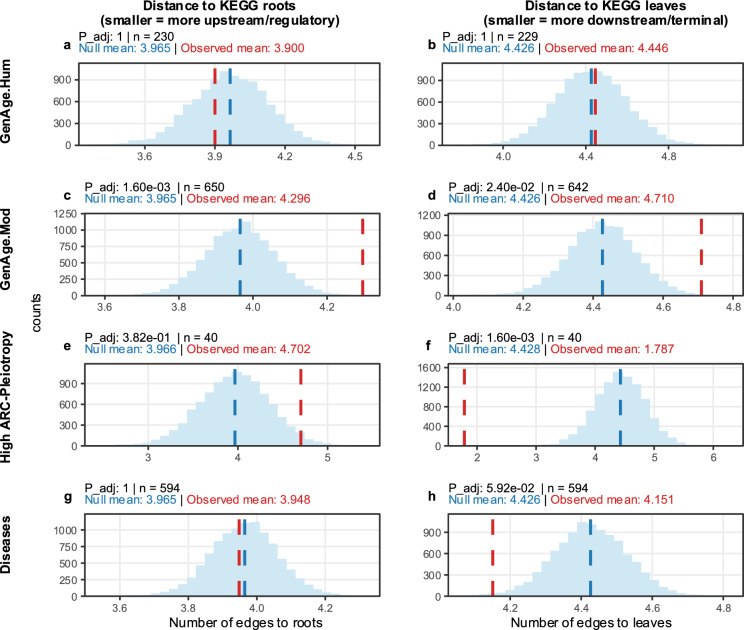
Permutation-based distributions of hierarchical distances to $KEGG$ roots and leaves. Each panel shows the permutation-derived null distribution (blue) of mean shortest-path distances within the $KEGG$ hierarchy, comparing the observed mean distance (red dashed line) against the null mean (blue dashed line). Distances were computed as the average number of edges separating each gene from all root nodes (left column) or from all leaf nodes (right column) in the directed acyclic structure of $KEGG$ pathways. Panels are organized by gene set (rows) and hierarchical reference (columns). From top to bottom: $GenAg{e}_{Hum}$, $GenAg{e}_{Mod}$, High *ARC-Pleiotropy*, and Diseases. From left to right, the plots correspond to distance to $KEGG$ roots and distance to $KEGG$ leaves. Smaller distances to leaves indicate a stronger concentration toward terminal, function-specific processes, while smaller distances to roots reflect closeness to upstream regulatory modules. The figure shows that highly *ARC-Pleiotropic* genes cluster near terminal leaves and far from roots, disease-related genes occupy intermediate downstream regions, and ${GenAge}_{Hum}$ genes maintain mid-hierarchy positions connecting regulatory and functional layers. In contrast, model-organism ageing-related genes exhibit a more evenly distributed profile across the hierarchy, with moderate distances to both roots and leaves. Adjusted p-values (p_adj) were computed using Bonferroni correction applied independently within each column (roots and leaves), accounting for the four gene sets evaluated per column

Human ageing-related genes** (**${GenAge}_{Hum}$**)** tended to occupy expected positions within the hierarchy, as their mean distances were virtually identical to those of random genes both from the roots (*3.90 vs. 3.965 null; ns*; Fig. 7a) and from the leaves (*4.446 vs. 4.426 null; ns*; Fig. 7b). This pattern indicates that human ageing-related genes are broadly distributed across the regulatory–functional spectrum, without a clear bias toward either upstream or downstream tiers.

Model-organism ageing-related genes ($${GenAge}_{Mod})$$, however, showed a significant shift toward more central positions within the KEGG cascade. Their mean distance to the roots was significantly larger than expected (*4.296 vs. 3.965 null; p_adj* = *1.6e-3*; Fig. 7c), while their distance to the leaves was also slightly greater than the null (*4.710 vs. 4.426 null; p_adj* = *2.4e-2*; Fig. 7d). This suggests that model ageing-related genes span multiple layers of the $$KEGG$$ hierarchy and preferentially occupy intermediate positions, rather than being confined to either upstream regulatory nodes or terminal process-specific branches.

In contrast, high *ARC-pleiotropy* genes, largely immunological in origin, displayed a strong hierarchical asymmetry. They were positioned farther from the regulatory roots than expected (*4.702 vs. 3.966 null; p* = *0.015*), although this difference did not remain significant after multiple-testing correction (*p_adj* = *3.82e − 1*; Fig. 7e). Conversely, these genes were located much closer than expected to the leaves (*1.787 vs. 4.428 null; p_adj* = *1.6e − 3*; Fig. 7f), revealing a pronounced concentration in the most downstream, process-specific regions of the KEGG hierarchy.

Disease-associated genes exhibited expected distances from the roots (3.965 vs. 3.948 null; ns; Fig. 7g) but showed a mild downstream bias in their distance to the leaves (4.151 vs. 4.426 null). Although this effect did not remain significant after multiple-testing correction (*p_adj* = *5.92e − 2*; Fig. 7h), it was nominally significant prior to correction (*p ≈ 7.3e − 3*), indicating that while disease-related genes remain partly anchored to mid-level regulatory layers, they tend to extend into functional endpoints.

Together, these results highlight that ageing-related genes, particularly those from model organisms, occupy intermediate to upstream regulatory tiers, bridging multiple pathway layers, whereas high *ARC-pleiotropy* immunological genes are strongly biased toward terminal branches, consistent with specialised processes. Supplementary Fig. 11 provides a graphical representation of this KEGG-based hierarchical gene placement.

### ML-based prediction of ageing-related genes

Having established how ageing-related genes connect to ARCs and ARDs, we next assessed whether these network-based features can be used to predict novel human ageing-related genes. Four connectivity metrics were evaluated (see Methods “ML-based Prediction of Ageing-Related Genes” and Supplementary Table 5, Supplementary Figs. 17 and 26): (i) shortest path length to ARCs/ARDs, (ii) mean path length to ARCs/ARDs, (iii) counts of neighbouring ARC/ARD-related genes per cluster, and (iv) the percentage of RWR visits when seeding random walks on ARC/ARD-related genes. Each metric was computed separately on the $PPI$, $KEGG$, $CO{X}_{90}$ and $CO{X}_{95}$ networks. Predictive performance was assessed at four levels: (a) for each individual metric–network combination, (b) per metric aggregated across networks, (c) per network aggregated across metrics, and (d) separately for ARC-based versus ARD-based definitions of the metrics. Finally, RWR was also applied to a *Multiplex* network integrating the four networks into distinct layers while preserving each layer’s topology and allowing inter-layer diffusion, thereby capturing complementary patterns of connectivity.

Balanced random-forest (BRF) predictions evaluated under cross-validation revealed that predictive power depended on the underlying network layer (see Supplementary Table 6 for details), with $COX_{95}$ and $COX_{90}$ yielding lower AUCs (≈0.56*–0.60* than KEGG and PPI (≈*0.81–0.82* for the highest metrics). Across metrics, diffusion-based measures consistently ranked among the strongest predictors, whereas disease *Neighbour* counts and *Proximity*-based variants tended to occupy intermediate positions, with ARC-based versions generally performing slightly below their ARD counterparts. The highest performance of all network configurations was achieved by the *Multiplex* setting, where RWR seeded on ARDs reached an AUC of ≈*0.88*, followed closely by the *average path length proximity to ARDs* aggregated across networks (≈*0.87*), indicating that diffusion-based metrics are particularly informative in this context.

### Candidate prediction and enrichment

BRF predictions yielded probability scores in [0–1] reflecting the estimated probability that a gene is associated with human ageing, as defined by ${GenAge}_{Hum}$, with 0.5 used as the decision threshold. Predictions from the *Multiplex* model were aggregated across the cross-validation folds, and non-GenAge genes classified as ageing-related were treated as the pool of candidate hits, from which we prioritised the highest-scoring subset. For downstream enrichment analyses, we selected the top 30 false positives (*i.e.* genes not annotated in GenAge but assigned the highest BRF scores). The top-ranking candidates included *SMAD2*, *SMAD4*, *CSNK2A1*, *HSP90AB1* and *MAPK1* (Supplementary Table 7).

Functional enrichment of these candidates, using the *Multiplex* gene universe as background, revealed a strong over-representation of intracellular signal transduction (GO:0035556, *p_adj* = *7.8e-14*), regulation of response to stimulus (GO:0048583, *p_adj* = *8.81e-14*), and programmed cell death (GO:0012501*, p_adj* = *5.06e-10*), among others (Supplementary Table 8).

## Discussion

Our study reveals two complementary modes by which genes can affect multiple ARDs. Ageing-related genes connect broadly to several ARCs through indirect interactors (ARC-Interactors), acting as network-based bridges, whereas highly ARC-Pleiotropic genes are directly associated with multiple ARCs—particularly immunological and systemic disorders—serving as shared nodes across clusters. This duality was consistent across PPI and KEGG networks: ageing-related genes occupied upstream positions in the KEGG hierarchy, while ARC-Pleiotropic genes concentrated near terminal, function-specific branches. Rather than treating Pleiotropy and Interactors as separate phenomena, our framework integrates them within a single analytical landscape that reflects the balance between global coordination and local specialization. Although disease interactions mediated by network neighbors usually outnumber direct associations for most genes, this pattern did not hold for aging-related genes: their direct overlap with ARD-associated genes was equal to (human) or lower than (model) random expectations, whereas their neighbour-mediated connectivity to ARCs was significantly higher than expected for size-matched random sets.

Our results show that highly pleiotropic genes are often tissue-specific and weakly integrated within molecular networks. Many appear isolated or show minimal coexpression, while those present in PPI or KEGG occupy peripheral or terminal positions, with only a few forming coherent coexpression clusters. Despite this limited integration, these genes are frequently associated with immune responses and chromatin organization—two functions inherently distributed across systems and cell types. This agrees with previous evidence that pleiotropic genes span diverse biological processes rather than forming a single functional class (Barrio-Hernández et al. 2023) and with regulatory analyses showing that shared eQTLs can produce pleiotropic effects without implying network centrality (Shikov et al. 2020). Similarly, the omnigenic model predicts that multi-trait influence can arise from peripheral rather than central nodes (Boyle et al. 2017), and evolutionary perspectives caution against assuming a unified mechanistic basis for pleiotropic ageing effects (Austad, 2018). Together, these views support that pleiotropy reflects dispersed, context-dependent mechanisms—particularly those linked to immune regulation and chromatin organization—rather than a single, network-centred ageing process (Paaby & Rockman 2013). These trends also agree with population-based studies showing that multimorbidity reflects shared genetic architecture across diseases, with ARC-Pleiotropic loci enriched in immune and metabolic pathways (Baltramonaityte et al. 2023a, b; Fu et al. 2024) and large-scale GWAS meta-analyses reporting similar convergence across ageing-related conditions (Lopera-Maya et al. 2022).

ML analyses confirmed that ARC-based network structure alone can predict ageing-related genes from their connectivity to ARD-associated genes. PPI and KEGG networks achieved the highest predictive scores for human ageing-related genes (GenAge-Hum), whereas the combined multiplex diffusion model outperformed all single layers, indicating that ageing-related information is distributed across complementary interaction contexts. This predictive capacity emerged without explicitly modelling ARC-Pleiotropy, suggesting that the spatial arrangement of ageing-related genes within ARCs carries intrinsic biological meaning. Top-ranked candidates were enriched for intracellular signalling, phosphorylation, and metabolic regulation pathways—particularly MAPK-mediated stress responses and *TGF-β/SMAD*-driven remodelling (Ren et al. 2023; Santos et al. 2021)—with *SMAD2*, *SMAD4*, *CSNK2A1*, *HSP90AB1* and *MAPK1* among the highest-scoring genes. These pathways have been repeatedly linked to systemic adaptation and cellular maintenance during ageing (Ren et al. 2023; Santos et al. 2021), reinforcing that ageing-related genes orchestrate stress and remodelling responses across tissues.

Several limitations warrant mention. Defining both direct and indirect associations solely from GWAS data is an inherent limitation of our work. As GWAS coverage expands, additional disease-related genes will inevitably reshape network context and broaden the operational meaning of pleiotropy—a conceptual fluidity long recognized in genetics (Paaby & Rockman 2013). Some indirect associations may nonetheless persist, particularly when a reference gene interacts with multiple neighbours and newly mapped GWAS genes for the same ARC occupy the same network neighbourhood. Although not universal, distinguishing between direct (pleiotropic) and indirect (network-mediated) associations remains a valuable interpretive framework for understanding gene–ageing relationships. On further limitations, SNP-to-gene assignment relied on proximity mapping (± 10 kb), which may miss long-range regulatory effects. Using eQTL, chromatin-contact or colocalization data could refine these relationships. GWAS-based ARC definitions depend on self-reported traits that may introduce noise, and ageing-gene catalogues such as *GenAge* are subject to curation bias. ML predictions also depend on the current completeness of networks; future GWAS and interaction data may refine specific gene-level outcomes. Given the limited and well-curated definition of ageing-related genes, their global trends are unlikely to change substantially as new data accumulate. In contrast, highly pleiotropic genes—although robust under our operational definition—could show different behaviours in alternative disease-mapping contexts, an avenue worth exploring in future analyses.

## Conclusions

Our results demonstrate that the broad disease impact of highly Pleiotropic genes does not require network centrality or broad expression. Rather than forming universal ageing-related cores, these genes often act within tissue-specific, weakly connected modules—a pattern consistent with previous reports that pleiotropic disease-related genes span diverse biological processes rather than collapsing onto a single functional axis.

Beyond these structural insights, our machine learning framework successfully predicted novel ageing-related gene candidates based on their connectivity to ARC- and ARD-associated modules. Many of these top-ranked genes belonged to conserved stress-response and signalling pathways—such as MAPK, TGF-β/SMAD, and phosphorylation cascades—reinforcing their role in systemic adaptation and maintenance during ageing.

Together, these results reveal a dual organization in the genetic architecture of ageing and multimorbidity: ageing-related genes act as cross-system integrators that maintain regulatory balance, whereas ARC-Pleiotropic genes operate as localized drivers of age-dependent disease vulnerability. Integrating these complementary perspectives provides a coherent framework for understanding how intrinsic ageing mechanisms and immune-mediated susceptibility jointly shape the landscape of human multimorbidity.

## Methods

### Retrieval of ARDs- and ageing-related genes

*Selection of ARDs*: GWAS summary statistics for non-cancer, self-reported diseases were obtained from the UK Biobank study of Dönertaş et al. (2021) (BioStudies ID: S-BSST407). That study derived age-of-onset profiles for 116 diseases and clustered them into four categories: (i) diseases with incidence increasing exponentially with age (n = 25); (ii) diseases increasing linearly with age (n = 51); (iii) diseases with no consistent age-of-onset pattern; and (iv) diseases emerging relatively early, around age 30. We focused on the 57 diseases with significant GWAS associations that showed linear or exponential age-related increases, hereafter referred to as ARDs.

*Retrieval of ageing-related genes:* Human ageing-related genes were obtained from the GenAge database (de Magalhães et al., 2024b) (Build 21 [28/08/2023]; retrieved on 10/09/25), which includes 307 curated genes (denoted as $GenAg{e}_{Hum}$). Ageing-related genes from model organisms (worm, fly, nematode and yeast) were retrieved from the GenAge (Model Organisms) database and mapped to human orthologs using the OMA Browser current release (Build 21 [28/08/2023]; retrieved on 10/09/25) (Altenhoff et al. 2015), yielding 1,147 human homologs (denoted as $GenAg{e}_{Mod}$) (downloaded 10/09/2025).

### SNP-to-gene mapping and association with ARDs/ARCs

The genomic ranges of protein-coding genes (GRCh38/hg38) were retrieved using the *biomaRt* package (Durinck et al. 2005), and significant SNPs were mapped to genes if they fell within the gene body or within ± 10 kb of its boundaries. This mapping was implemented with the *GenomicRanges* package (Lawrence et al. 2013). When a SNP overlapped with more than one gene, it was assigned to all candidate genes; conversely, if a gene contained SNPs associated with multiple diseases, it was associated to each of those diseases. This inclusive *Proximity*-based strategy, consistent with common practice in GWAS-to-gene mapping (Gazal et al. 2022), avoids discarding plausible gene–disease associations and accounts for potential *ARC-Pleiotropy*.

Applying this procedure to GWAS results for 57 ARDs, we identified 2588 genes significantly associated with at least one ARD. Each of these associations constitutes what we refer to as a *Gene–ARD association*.

### Clustering of ARDs into ARCs

The UK Biobank organizes self-reported diseases hierarchically into main diagnostic categories (e.g., cardiovascular, haematological), each containing specific subcategories representing related clinical traits (UK Biobank: Data-Field 20,002). This hierarchical structure was used as the basis for grouping the retrieved ARDs into broader ARDs Clusters (ARCs). For example, hypertension, heart attack, and arrhythmia were all classified as cardiovascular ARDs, forming the Cardiovascular ARC (Supplementary Fig. 1).

This approach leverages the intrinsic disease taxonomy of the UK Biobank to capture shared pathophysiological mechanisms within major organ systems while minimizing redundancy among highly correlated ARDs. Each ARC thus represents a higher-order disease domain summarizing a physiologically coherent set of ARDs.

Gene–ARC associations were defined by assigning each gene associated with at least one ARD within a given cluster as ARC-associated. Because a gene could be associated to ARDs belonging to multiple clusters (i.e., ARCs), this framework allowed quantification of cross ARD-cluster (i.e., cross ARC) genetic overlap and enabled subsequent analyses of *ARC-Pleiotropy*.

In total, the 57 ARDs analyzed in this study were grouped into 8 non-overlapping ARCs (Table 4): *Cardiovascular*, *Endocrine/Diabetes*, *Gastrointestinal/Abdominal*, *Musculoskeletal/Trauma*, *Neurology/Eye/Psychiatry*, *Immunological/Systemic Disorders*, *Haematology/Dermatology*, and *Respiratory*. For further information, see Supplementary Materials: “ARDs Clusters (ARCs)” and Supplementary Fig. 1.

**Table 4:** ARDs grouped into eight non-overlapping ARCs according to the UK Biobank disease classification

ARC	Number of ARDs	Number of genes
Cardiovascular	13	1406
Gastrointestinal/Abdominal	12	275
Musculoskeletal/Trauma	11	359
Neurology/Eye/Psychiatry	8	184
Endocrine/Diabetes	6	1074
Haematology/Dermatology	3	16
Renal/Urology	3	11
Immunological/Systemic Disorders	1	148

The table shows the number of ARDs included in each ARC

### ARC networks

We complemented the Gene–ARC relationships by integrating gene–gene connections from three databases: *BioGRID* for $PPI$, *GeneFriends* for coexpression, and $KEGG$ for pathways.

$PPI$ network: Human $PPI$ data were retrieved from *BioGRID* (Oughtred et al 2021) (physical interactions, version 4.4.248; retrieved 20/08/2025) and used to construct an unweighted network with the *igraph* package. The resulting network contained 11,837 nodes (genes) and 93,086 edges (interactions). We then mapped ARCs and ageing-related genes onto this network, retaining only those present in *BioGRID* to ensure that all genes had at least one edge (Supplementary Fig. 5).

$COX$ networks: Coexpression data were obtained from *GeneFriends* (version 5; retrieved 20/08/2025), which provides a square matrix of 44,947 genes with pairwise coexpression values (Raina et al. 2023). *Ensembl* identifiers were converted to *HGNC* symbols using *biomaRt*, yielding 32,915 mapped genes. Two binary adjacency matrices were generated by applying absolute correlation thresholds of 90% and 95%. For each threshold, gene pairs exceeding the cutoff were assigned an edge and assembled into two-column edge lists, which were converted into networks with *igraph*. These networks are referred to as $CO{X}_{90}$ (11,136 nodes, and 1,463,273 edges; Supplementary Fig. 7) and $CO{X}_{95}$ (5,106 nodes and 141,865 edges; Supplementary Fig. 9).

$KEGG$ network: Gene associations were retrieved from $KEGG$ pathways (Kanehisa 2019; Kanehisa et al. 2025; Kanehisa et al*.*, 2000) using the *KEGGlinks* library (White & Medvedovic 2016a, b). Individual pathway structures were parsed with *KEGGgraph* (Zhang et al. 2009) (retrieved 20/08/2025), generating interaction data frames for ~ 250 human pathways. After concatenating and removing duplicates, the resulting undirected network contained 6,320 nodes and 59,586 edges **(**Supplementary Fig. 11**)**.

In all cases, only ARCs and ageing-related genes overlapping with the respective network were retained for downstream analyses (for further information see Supplementary Materials: “Genetic Networks of Ageing and ARDs”, Supplementary Figs. 5–11, Supplementary Table 3).

### Identification of genes with ARC-Pleiotropy and ARC-interactions

We define *ARC-Pleiotropy* as the number of phenotypes with which a gene is directly associated through GWAS, and indirect interactions as the number of phenotypes a gene can influence through its network connections to GWAS-associated genes. Both measures can be computed at the level of individual diseases (*i.e.*, ARDs) or disease clusters (*i.e.*, ARCs), yielding four complementary categories: *ARD-Pleiotropy*, *ARC-Pleiotropy*, *ARD-Interactions*, and *ARC-Interactions* (Supplementary Fig. 13).

Importantly, ARD-level measures do not necessarily translate to ARC-level ones. For example, a gene connected to several ARDs within the same ARC displays *ARD-Pleiotropy* but not *ARC-Pleiotropy*, whereas a gene associated with fewer ARDs from distinct ARCs exhibits higher *ARC-Pleiotropy*. The same logic applies to indirect interactions. Moreover, genes can simultaneously be *ARC-Pleiotropic* and *ARC-Interactive*, reflecting both direct and indirect modes of connectivity across ARDs.

We illustrate these definitions with simple examples:

*ARD-Pleiotropy*: Number of individual ARDs with which a gene shows a significant GWAS association. *Example:* If gene $X$ is associated with *stroke* and *hypertension* in GWAS, its *ARD-Pleiotropy* = 2.

*ARC-Pleiotropy*: Number of distinct ARCs with which a gene is associated through GWAS. A gene is associated with an ARC if it has at least one GWAS association with an ARD belonging to that ARC. *Example:* If gene $X$ is associated with *stroke* and *hypertension* (both within the Cardiovascular ARC) and with *arthritis* (Musculoskeletal ARC), its *ARC-Pleiotropy* = 2.

*ARD-Interactions*: Number of ARDs indirectly connected to a gene through at least one neighbour in the network that is GWAS-associated with that ARD. Only genes present in the corresponding network are considered. *Example:* If gene $X$ has no direct GWAS hits but interacts in $PPI$ with gene $Y$ (GWAS-significant for *stroke*) and gene $Z$ (GWAS-significant for *diabetes*), then $X$ has *ARD-Interactions* = *2*.

*ARC-Interactions*: Number of ARCs indirectly connected to a gene through neighbours associated with at least one ARD in those ARCs. Only genes present in the corresponding network are considered. *Example:* Using the same case, if gene $Y$ belongs to the Cardiovascular ARC (*stroke*) and gene *Z* to the Endocrine ARC (*diabetes*), then gene $X$ has *ARC-Interactions* = 2.

Further explanations can be found in the Supplementary Materials, specifically in the section titled “ARC Interactors” and in Supplementary Fig. 13.

Moreover, we defined the following two classifications of ARC-Pleiotropy:

- *High ARC-Pleiotopy*: genes directly associated with 4 or more distinct ARCs.
- *Low ARC-Pleiotopy*: genes directly associated with 1–3 distinct ARCs.

### ARC-interactions analysis

Genes were classified into five gene sets: human ageing-related genes ($GenAg{e}_{Hum}$), model organisms ageing-related genes ($GenAg{e}_{Mod}$), *Diseases* (genes associated with any ARC through GWAS), *Neighbours* (genes interacting with at least one *ARC-associated* gene), and *High ARC-Pleiotropy* (genes associated with multiple ARCs).

For each of the four networks ($PPI$, $CO{X}_{90}$, $CO{X}_{95}$, and $KEGG$), we quantified for each gene the number of *ARC-Interactions*, defined as the number of distinct ARCs containing at least one gene directly interacting with it in the network. Statistical comparisons were performed pairwise between the five gene sets, based on the mean number of ARC-Interactions per gene. All genes assigned to a gene set were included in that set’s analysis, even if they also appeared in other sets. This approach, commonly used for overlapping gene sets, captures group-level trends without excluding shared members.

Because set sizes and data distributions differed, and since ARC-Interaction values are discrete counts rather than continuous variables, we used the Wilcoxon rank-sum test (Mann–Whitney U) for skewed data. The distribution of ARC-Interactions was strongly right-skewed, with most genes showing low interaction counts, making non-parametric testing more appropriate. Pairwise comparisons were adjusted for multiple testing using Bonferroni correction.

In addition, we performed permutation tests to assess whether the mean values observed for each group were higher or lower than expected by chance. For each category, we generated a null distribution by randomly sampling the same number of genes from the background of genes present in the respective network, repeating this procedure 10,000 times. The observed group mean was then compared to this null distribution, and empirical two-tailed *p*-values were calculated as the proportion of permuted means as extreme as, or more extreme than, the observed mean.

### Shortest and average paths to diseases

To quantify how ageing-related genes are positioned relative to ARC- and ARD-associated genes within molecular networks, we computed their genetic distance based on the minimum number of edges connecting them. For any two genes, the shortest distance (*Shortest Path Distance*) was defined as the smallest number of edges along the shortest path between the reference gene and a target gene. Under this definition, *shortest path distance* = *0* when the two genes are identical and becomes infinite when no path connects them. In the context of ARCs or ARDs, this metric represents the shortest path from a given reference gene to any gene associated with the target ARC or ARD.

Because some genes are disconnected in sparse networks (yielding infinite distances) and others coincide with the reference gene (*distance* = *0*), we employed a bounded *Proximity* transformation to normalize values and allow averaging across all pairwise relationships. We defined *Proximity* as:

$$Proximity=\frac{1}{Shortest.distance+1}$$

This formulation ensures that *Proximity* values remain within the [0, 1] interval—equal to 1 when two genes overlap (*distance* = *0*) and approaching 0 as distance increases toward infinity. Thus, disconnected gene pairs contribute a *Proximity* of 0, while self-connections contribute 1.

For each gene, two complementary *Proximity* metrics were computed relative to each ARC or ARD:

- *Shortest Path Proximity*: the maximum *Proximity* value, corresponding to the shortest path (*i.e.*, nearest connection) between the reference gene and any ARC/ARD-associated gene (Supplementary Fig. 16).
- *Average Path Proximity*: the mean of *Proximity* values between the reference gene and all genes associated with the ARC/ARD of interest, where disconnected pairs (infinite distance) contribute zero.

For each gene, *Proximity* values were first calculated independently for each of the ARCs, using either *Shortest Path Proximity* or *Average Path Proximity* depending on the metric. These *Proximity* values were then averaged across all ARCs to obtain a single representative measure of how closely each gene is positioned, on average, to age-related disease modules within the network.

To express results in intuitive distance units, this mean *Proximity* was then converted back into its equivalent distance using the inverse transformation:

$$Distance=\frac{1}{Proximity}-1$$

where *Proximity* corresponds to the mean *Shortest Path Proximity* or mean *Average* *Path Proximity* value. This approach ensures that disconnected genes (with infinite distances) contribute to a *Proximity* of 0, directly connected or overlapping genes contribute a *Proximity* of 1, and the final mean genetic distance to ARCs reflects the typical network separation between each gene and the overall architecture of ARCs.

Further explanations on *Proximity* can be found in the “Topological association of genes with ARCs (description) – subsection: Shortest path distance to Diseases (proximity analysis)” of Supplementary Materials and Supplementary Fig. 16.

### Random walk with restart

To complement shortest-path–based measures, we applied RWR on both individual networks (*Monoplex*) and a *Multiplex* network integrating all interaction layers, consistent with recent network-based frameworks (*e.g.* Koo & Pan 2024). RWR is a network propagation algorithm that estimates the relative influence of a set of “seed” nodes by simulating a walker that, at each step, either (i) moves to a neighbouring node with probability *(1–r)* or (ii) returns to the seed set with probability *r* (Baptista et al. 2022). After convergence, each gene receives a steady-state probability reflecting its *association* to the seed set, allowing all genes in the network to be ranked by functional association to the seeds.

*Networks*: RWR was applied independently to four networks: $PPI$ (*BioGRID*), coexpression at 90% and 95% thresholds (*GeneFriends*), and $KEGG$ pathways, as well as to a *Multiplex* network combining all four layers. In each case, the analysis was restricted to the genes present in the respective network. For the *Multiplex*, the combined set of genes across the four layers was used; when a gene was absent from a particular layer, it was included as an isolated gene to maintain consistency across layers.

*Seeds and runs*: We performed multiple independent RWR runs, each using a different seed set. Two types of seed sets were defined: (i) ARD-level, with 57 runs, each using the GWAS-associated genes of a single ARD, and (ii) ARC-level, with 8 runs, each using the genes associated with one ARC. This resulted in 65 runs per network. Since the procedure was applied to four *Monoplex* networks, this yielded 260 runs in total. The same procedure was repeated on the *Multiplex* network, providing another 65 runs. Altogether, the framework encompassed 325 independent RWR analyses.

*Parameters*: Following standard practice, the restart probability was set to *r* = 0.7. At each iteration, the walker has a 70% chance of returning to the seed set and a 30% chance of exploring neighbours. When returning, the probability of re-starting at any given seed gene is shared equally among all seeds (*e.g.*, if *r* = 0.7 and there are 10 seeds, each gene has a 7% chance of being chosen). This ensures that all seeds contribute equally, preventing bias toward a single gene.

Multiplex framework: In the *Multiplex* framework (Cozzo et al. 2018), the algorithm also requires an inter-layer parameter (τ). Once the walker decides to explore (the 30% case), τ determines how this exploration is distributed across the different layers ($PPI$, $CO{X}_{90}$, $CO{X}_{95}$, $KEGG$). A τ vector assigns one weight per layer, acting as relative preferences. For example, τ = [1,1,1,1] gives equal weight to all four layers, while τ = [2,1,1,1] would make transitions through the $PPI$ layer twice as likely as through the others. In simple terms, *r* decides “go back to the seeds or keep exploring?”, and if the walker keeps exploring, τ decides “through which type of network connections?”. If the walker reaches a gene in one layer and τ indicates moving to another layer where that gene does not originally exist, the algorithm represents it as an isolated gene in that layer. In such cases, the walker remains at that isolated gene until either *r* returns it to the seed set or τ allows it to jump to another layer where the same gene has valid connections.

*Implementation*: We used the R package *RandomWalkRestartMH* to construct *Multiplex* objects (Valdeolivas et al. 2018), compute normalized supra-adjacency matrices (block-matrix representations of intra- and inter-layer connections) and propagate scores. For each seed set (ARD or ARC), RWR was run with the corresponding genes, and scores were obtained for all genes in the network. Global random state was set to 42 to ensure reproducibility.

*Output*: This procedure generated an RWR score for every gene relative to each seed set, producing complete $gene \times ARD$ and $gene \times ARC$ association matrices for each network. To facilitate comparisons, scores were standardized as Z-scores within each seed set. From these matrices, we derived: (i) association tables reporting RWR scores and ranks of all genes, (ii) per-gene summaries of mean association across ARDs and ARCs, and (iii) top-ARD and top-ARC assignments identifying the highest score for each gene. These outputs were used in downstream analyses, including network comparisons and integration with ML classifiers.

Further explanations on *RWR* can be found in the “Topological association of genes with ARCs (description) – subsection: Random Walk with Restart” of Supplementary Materials and Supplementary Fig. 17.

### Gene coexpression analysis

We retrieved the human coexpression matrix from *GeneFriends* (Raina et al. 2023), which contains 44,947 genes (*Ensembl* identifiers). Using the *biomaRt* package (Durinck et al. 2005), we mapped *Ensembl* to HGNC symbols and retained genes associated with at least one ARC, $GenAg{e}_{Hum}$, or $GenAg{e}_{Mod}$ (3897 in total). After removing 18 duplicates with conflicting HGNC mappings, the final matrix contained 3879 genes. All analyses were performed on the absolute values of coexpression.

In this context, a *group* refers to any predefined gene set, such as an ARC (*e.g.*, cardiovascular), an *ARC-Pleiotropy* category, or the $GenAg{e}_{Hum}$ and $GenAg{e}_{Mod}$ sets.

*Intra-set coexpression*: For each group, we extracted the square submatrix whose rows and columns corresponded to its genes (*e.g.*, cardiovascular ARC-related genes with themselves). To avoid duplicates, we converted the lower triangle of each submatrix into a numeric array. The mean of this array was taken as the *intra-set coexpression* of the group. These group-level means were compared using two-sided t-tests with Bonferroni correction (Main text Figs. 3 and 4a, c; Supplementary Fig. 20).

*Inter-set coexpression*: For each pair of distinct groups (*e.g.*, cardiovascular ARC versus endocrine ARC), we extracted the corresponding submatrix of correlations. To ensure that every gene pair was counted once and only once, values were collected in three steps: (i) correlations among overlapping genes, restricted to the upper triangle to avoid duplicates; (ii) correlations between non-overlapping genes of group 1 with all genes of group 2; (iii) correlations between non-overlapping genes of group 2 with all genes of group 1.

This separation was necessary because groups could partially overlap, and without distinguishing these cases we would either lose valid correlations or count some pairs more than once. The resulting values from all three components were merged into a single array, and the mean of this array was taken as the *inter-set coexpression* between the two groups.

### Tissue specificity analysis

We obtained tissue specificity data from the *Tau Index of Gene Tissue Specificity* section of the HAGR database (de Magalhães et al. 2009, 2024; Palmer et al. 2021). The file contains 39,366 genes across 30 GTEx tissues (Lonsdale et al*.*, 2013), with expression levels in RPKM and a pre-computed *Tau* index. *Tau* ranges from 0 (uniform expression across tissues) to 1 (expression restricted to one or few tissues).

Differences in *Tau* distributions were assessed using two-sided Wilcoxon rank-sum tests, comparing both network-independent groups (ARCs, *ARC-Pleiotropy* categories, $GenAg{e}_{Hum}$, and $GenAg{e}_{Mod}$) and network-dependent groups (*ARC-Interaction* categories). This non-parametric approach was selected given the bimodal distribution of *Tau* values at low and high ranges. Where multiple pairwise comparisons were performed, *p*-values were corrected using the Bonferroni method. Genes without *Tau* annotation were excluded (< 1% of the dataset). In addition, expression values (RPKM) were inspected across individual tissues to ensure that observed *Tau* differences reflected genuine expression patterns rather than missing values or outliers (Main text Figs. 4b,c and 6; Supplementary Fig. 21).

### ${\mathrm{K}}{\mathrm{E}}{\mathrm{G}}{\mathrm{G}}$ hierarchy analysis

The $KEGG$ pathway structure was represented as a directed acyclic graph (DAG) using *igraph* (R). Edges were built from the $KEGG$ interactions table and simplified to remove self-loops and multi-edges. In-degree and out-degree were used to identify roots (in-degree = 0) and leaves (out-degree = 0) in the original directed graph.

For each gene (vertex), shortest-path distances to reachable leaves were computed on the original directed graph (mode = “out”). Distances to reachable roots were computed on the edge-reversed graph (so that roots in the original graph become reachable via out-paths). Only nodes connected through valid directional paths within the same branch were considered; disconnected targets were excluded (infinite distances removed). Per-gene summaries were obtained as the mean of finite distances, yielding *leaf_mean* and *root_mean*. Degree (in/out/all) and betweenness centrality were also computed on the original graph.

Gene sets were defined as follows: human ageing-related genes ($GenAg{e}_{Hum}$), model-organism ageing-related genes ($GenAg{e}_{Mod}$), *highly ARC-Pleiotropic* genes (immunological/systemic disorders subset), and disease-associated genes (union of ARD/ARC mappings). For each set and each metric (*leaf_mean*, *root_mean*), a permutation test (10,000 iterations; size-matched sampling without replacement from all $KEGG$-annotated genes) generated a null distribution of the mean. Two-sided p-values were obtained from tail counts and Bonferroni adjustment was applied across tests. Effect size was reported as the difference between observed and null means; z-scores were computed using the null standard deviation when finite.

Visualization of null distributions and observed means followed a standardized histogram layout (*ggplot2/ggtext*), and multi-panel grids were assembled with cowplot.

### Permutation framework

All permutation analyses were designed to evaluate whether the observed properties of ageing-related and *ARC-Pleiotropic* genes departed from random expectation. For each test, we constructed 10,000 size-matched random samples from the relevant background universe and recalculated the metric of interest, generating null distributions against which observed values were compared. To avoid *p*-values of zero when no permutation exceeded the observed statistic, we applied the standard correction of (r + 1)/(n + 1), where *r* is the number of permutations with values equal to or greater than the observation and *n* is the total number of permutations. Under this scheme, the minimum achievable *p*-value is 1/10,001 (~ 1e-4). Reported *p*-values thus represent conservative estimates, further corrected for multiple testing when necessary. All random seeds used in permutations were set to 42 to ensure reproducibility.

### ARC-Pleiotropy of ageing-related genes

To determine whether ageing-related genes display unusual levels of *ARC-Pleiotropy*, we compared the observed distribution of *ARC-Pleiotropy* in $GenAg{e}_{Hum}$ and $GenAg{e}_{Mod}$ to null distributions generated from 10,000 size-matched random samples of the full set of human protein-coding genes (20,063 genes, retrieved from *EnsDb.Hsapiens.v86*). This approach controlled for the fact that *ARC-Pleiotropy* is defined based on direct GWAS signals, not on network embedding.

### ARC-Interactor

To test whether different gene groups displayed broader indirect connectivity than expected, we calculated the number of *ARC-Interactions* (indirect interactions with ARCs-associated genes through network neighbours) for ageing-related genes ($GenAg{e}_{Hum}$, $GenAg{e}_{Mod}$), disease-associated genes, and highly *ARC-Pleiotropic* immunological genes. Null distributions were generated from 10,000 size-matched random samples drawn from the set of genes present in each network ($PPI$, $KEGG$, $CO{X}_{90}$, $CO{X}_{95}$). This ensured that comparisons were made against genes with equivalent embedding in each network layer.

### Proximity-based tests

For each gene group, we computed the minimum shortest path distance to ARC-associated modules across the four network layers. Observed mean distances were contrasted against null distributions obtained from 10,000 size-matched random samples restricted to the genes present in each network. This tested whether ageing-related genes tend to occupy positions closer to disease modules than expected under random topology.

#### RWR

We applied RWR from ARC-associated seeds to estimate global accessibility of each gene. The stationary probability of revisiting each gene during the diffusion process was used as a connectivity score. Null expectations were derived from 10,000 size-matched random samples drawn from the gene universe of each network layer, ensuring that deviations reflected topological enrichment beyond network-specific baselines.

### Gene coexpression

We assessed intra- and inter-group coexpression levels by calculating the mean pairwise correlation within and across ARCs, ageing-related sets, and *ARC-Pleiotropy* strata. Null expectations were generated by 10,000 size-matched random samples obtained by shuffling gene membership while preserving group sizes, sampling from the entire set of genes included in *GeneFriends* (the coexpression database). This tested whether the coherence or cross-coherence of specific groups exceeded that expected for random sets of genes with equivalent representation in the resource.

### Tissue specificity

We compared the distribution of tissue-specificity scores (τ values) of ageing-related and *ARC-Pleiotropic* genes against random expectations. Null distributions were generated from 10,000 size-matched random samples of the full set of genes with *Tau* values available in GTEx, ensuring that observed enrichments reflected deviations from the transcriptome-wide baseline of tissue specificity.

### KEGG

For each set and each metric (*leaf_mean*, *root_mean*), a permutation test (10,000 iterations; size-matched sampling without replacement from all $KEGG$-annotated genes) generated a null distribution of the mean. Two-sided p-values were obtained from tail counts and Bonferroni adjustment was applied across tests. Effect size was reported as the difference between observed and null means; z-scores were computed using the null standard deviation when finite.

### ML-based prediction of ageing-related genes

We implemented a supervised learning framework to predict ageing-related genes based on their topological relationships with ARD- and ARC-related genes within networks. Each gene was represented as a feature vector comprising four network-derived metrics that quantify gene–disease associations through different aspects of network topology: shortest-path proximity, mean path length, ARC/ARD neighbour counts, and RWR diffusion probabilities. These metrics, described in detail in the following section, capture complementary dimensions of network integration and were computed across the four network layers ($PPI$, $KEGG$, ${COX}_{90}$, and ${COX}_{95}$).

### Definition of connectivity measures

We used the *Proximity* and RWR formulations (defined in the Methods sections: “Shortest and average paths to diseases” and “Random Walk with Restart”, respectively), to define four complementary measures of connectivity to capture different network scales:

- Shortest path *Proximity*. The highest *Proximity* between a gene and any ARC- or ARD-associated target, emphasizing the most direct relationship.
- Average path *Proximity*. The mean *Proximity* to all ARC/ARD-associated genes, reflecting how broadly a gene is embedded within disease or ageing modules.
- Disease-associated neighbours. The number of immediate interactors of a gene that are annotated as ARC- or ARD-associated, capturing direct adjacency.
- RWR seeded from diseases. A diffusion process in which a virtual walker starting from disease-associated nodes randomly explores the network, periodically returning to the seed set. The resulting steady-state probabilities represent how strongly a gene can be reached from disease modules through both local and long-range connections.

We chose *Proximity* over the number of edges in the shortest or average path because raw path length suffers from two key limitations: it yields infinite values for genes not connected to any disease-associated node, and its scale varies across networks of different size and density, making cross-network comparisons inconsistent. In contrast, *Proximity*—defined as the inverse of path length plus one—provides a bounded, continuous measure of network closeness that remains comparable across layers.

Together, these metrics provide a multiscale representation of network connectivity, spanning from local genetic interactions (*Disease-associated Neighbours* and *Shortest-path Proximity*) to distributed influence across signalling and coexpression (*Average-path Proximity* and *RWR seeded from diseases*).

For further information, see Supplementary Figs. 16–17, 26 and Supplementary Table 5.

### Dataset construction

Connectivity scores were computed across the $PPI$, $CO{X}_{90}$, $CO{X}_{95}$ and $KEGG$ networks.

For each gene in every network, we generated two independent feature sets:

- **ARC-based features:** *Proximity* to each of the eight ARCs (8 features per gene).
- **ARD-based features:** *Proximity* to each of the 57 ARDs (57 features per gene).

ARC- and ARD-based datasets were analysed separately to compare cluster-level and disease-level patterns of connectivity. To capture complementary structure, we created (i) $Metric \times Network$ datasets combining all *Proximity* measures across networks, (ii) per-network and per-metric integrations aggregating information within or across layers, and (iii) a *Multiplex* RWR analysis uniting the $PPI$, $KEGG$, and coexpression layers into a multilayer diffusion model. This strategy yielded 47 datasets (Supplementary Table 6; Supplementary Fig. 26) that systematically represent local, path-based, and diffusion-based relationships.

Further details are presented in Supplementary Materials (“ML-based Prediction of Ageing-Related Genes – subsection: Dataset construction”).

### Machine learning implementation

We used Balanced Random Forests (BRF), an ensemble method designed for imbalanced datasets (Pedregosa et al. 2011; Lemaître et al. 2017), implemented through the *imbalanced-learn* and *scikit-learn Python* libraries (version ≥ 1.3). In our setting, ageing-related genes represented fewer than 5% of all genes. BRF models were trained with 500 trees, and hyperparameters were optimized using nested cross-validation (10 outer folds, 5 inner folds) with the *GridSearchCV* module from *scikit-learn*.

### Model evaluation

Predictive performance was evaluated using the area under the ROC curve (AUC), a threshold-independent metric robust to class imbalance. BRF models return a continuous probability score between 0 and 1 reflecting the likelihood that a gene is ageing-related. For binary classification, we adopted a default threshold of 0.5, such that genes scoring above this value were predicted as ageing-related (*i.e.*, associated with ${GenAge}_{Hum}$).

### Identification of potentially novel ageing-related genes

Novel ageing-related gene candidates were identified by ranking all non-${GenAge}_{Hum}$ genes based on their predicted ageing-relatedness probability scores from the BRF. The top 30 ranked genes, absent from current annotations, were prioritized as promising candidates for further analysis.

### Gene set enrichment analyses

Functional enrichment analyses were carried out with *clusterProfiler* (Xu et al. 2024), using *org.Hs.eg.db* for gene annotation and *GOSemSim* for redundancy reduction of GO terms based on semantic similarity (Huber et al. 2015; Carlson 2023). All analyses were restricted to Biological Process (GO:BP) terms, with a *p*-value cutoff of 0.05, a minimum gene set size of 15, and redundant categories collapsed at a similarity threshold of 0.8. Importantly, enrichments were performed against custom backgrounds tailored to each analysis.

For highly *ARC-Pleiotropic* immunological genes, the background was the full set of human protein-coding genes (20,063 from *EnsDb.Hsapiens.v86*), since using only disease-associated genes (n = 2,503) yielded no significant results.

For ageing-related genes, enrichments were conducted separately for $GenAg{e}_{Hum}$-exclusive, $GenAg{e}_{Mod}$-exclusive, and intersecting genes, always using the union of both databases as the background.

For novel candidates predicted by the *Multiplex* model, the background was defined as all genes present in the four ARC networks ($PPI$, $KEGG$, $CO{X}_{90}$, $CO{X}_{95}$), while for candidates predicted by single-network models, the background was restricted to the genes present in the corresponding network. In both cases of predicted genes, enrichment was performed on the top 30 false positives—genes not annotated in GenAge but predicted as ageing-related with the highest probability scores—so that results reflect the most confident candidates.

## Supplementary Information

Below is the link to the electronic supplementary material.

Supplementary file1 (PDF 7723 KB)

## Data Availability

The datasets generated during this study are publicly available through the Synapse data-sharing platform. All data can be accessed under the project *Pleiotropy and disease interactors* (Synapse ID: syn72037936; https://www.synapse.org/Synapse:syn72037936/files/), with organized subfolders in the “Files” section corresponding to retrieved source data and generated results.

## Code availability

All scripts used to generate the results reported in this study are publicly available athttps://github.com/maglab/Gustavo/tree/main/Code.

## Glossary

ARDs – Ageing-Related Diseases: Diseases whose incidence increases with age, identified through GWAS or epidemiological studies
ARD-Pleiotropy: Number of distinct age-related diseases (ARDs) with which a gene shows a direct GWAS association, representing its disease-level pleiotropy
ARCs – ARD-Clusters: Group of ARDs that share common physiological or pathological features. ARCs were defined based on the hierarchical organization of self-reported diseases in the UK Biobank, which groups related conditions into broader categories (e.g., cardiovascular, metabolic, neurological)
ARC-Pleiotropy: Number of distinct disease clusters (ARCs) to which a gene is linked through a GWAS association with at least one of their ARDs, reflecting its cross-system or multi-cluster genetic influence.
ARC-Interactor: A gene whose immediate network neighbour is a GWAS-associated gene of an ARD (and therefore of the ARC)
COX_90_/COX_95_Network – Coexpression Networks (90% / 95% thresholds): Gene coexpression networks built from correlation data, applying strict thresholds (90% or 95%) to define strong coexpression links.
GeneAge_Hum_Human Ageing-Related Genes: Human genes associated with ageing, curated in the GenAge database.
GeneAge_Mod_Model Organism Ageing-Related Genes: Human homologs of ageing-associated genes originally identified in model organisms.
High ARC-Pleiotopy: genes directly associated with 4 or more distinct ARCs.
Inter-set coexpression: The correlation of gene expression levels between different gene sets (e.g., cardiovascular genes with endocrine genes). High inter-set coexpression indicates that genes in different gene sets share similar expression patterns.
Intra-set coexpression: The correlation of gene expression levels within the same group (e.g., cardiovascular genes with each other). High intra-set coexpression indicates that genes in the same gene set share similar expression patterns.
KEGG – Kyoto Encyclopedia of Genes and Genomes: A database of biological pathways, molecular interactions, and systems, often used for enrichment and network analyses.
Low ARC-Pleiotopy: genes directly associated with 1-3 distinct ARCs
Neighbour Genes (Neighbours): Genes that interact with ARC-associated genes but are not themselves directly linked to ARCs.
*PPI* Network – Protein–Protein Interaction Network: Carbon nanotube designed to operate vertically to substrate surface. It possess high surface area and provide stationed electrical property.

## References

[CR1] Altenhoff AM, Škunca N, Glover N, Train CM, Sueki A, Piližota I, Gori K, Tomiczek B, Müller S, Redestig H, Gonnet GH, Dessimoz C (2015) The OMA orthology database in 2015: function predictions, better plant support, synteny view and other improvements. Nucleic Acids Res 43(Database issue):D240–D249. 10.1093/nar/gku1158 25399418 10.1093/nar/gku1158 PMC4383958

[CR2] Austad SN, Hoffman JM (2018) Is antagonistic *ARC-Pleiotropy* ubiquitous in aging biology? Evol, Med, Public Health 2018(1):287–294. 10.1093/emph/eoy033 30524730 10.1093/emph/eoy033 PMC6276058

[CR3] Baltramonaityte V, Pingault J-B, Cecil CAM, Choudhary P, Järvelin M-R, Penninx BWJH, Felix J, Sebert S, Milaneschi Y, Walton E (2023a) A multivariate genome-wide association study of psycho-cardiometabolic multimorbidity. PLoS Genet 19(6):e1010508. 10.1371/journal.pgen.1010508 37390107 10.1371/journal.pgen.1010508 PMC10343069

[CR4] Baptista A, Gonzalez A, Baudot A (2022) Universal multilayer network exploration by random walk with restart. Commun Phys 5:170. 10.1038/s42005-022-00937-9

[CR5] Barrio-Hernández I, Schwartzentruber J, Shrivastava A et al (2023) Network expansion of genetic associations defines a pleiotropy map of human cell biology. Nat Genet 55:389–398. 10.1038/s41588-023-01327-9 36823319 10.1038/s41588-023-01327-9 PMC10011132

[CR6] Boyle EA, Li YI, Pritchard JK (2017) An expanded view of complex traits: from polygenic to omnigenic. Cell 169(7):1177–1186. 10.1016/j.cell.2017.05.038 28622505 10.1016/j.cell.2017.05.038 PMC5536862

[CR7] Carlson M (2023) *org.Hs.eg.db: Genome wide annotation for Human*. R package (Bioconductor). 10.18129/B9.bioc.org.Hs.eg.db

[CR8] Cozzo E, de Arruda GF, Rodrigues FA, Moreno Y (2018) *Multiplex Networks: Basic Formalism and Structural Properties* (SpringerBriefs in Complexity). Springer Cham. 10.1007/978-3-319-92255-3

[CR9] Csárdi G, Nepusz T, Traag V, Horvát S, Zanini, F, Noom D, Müller K (2023) igraph: Network analysis and visualization in R (R package version 1.5.1) [Computer software]. *Zenodo*. 10.5281/zenodo.7682609

[CR10] de Magalhães JP (2024a) Distinguishing between driver and passenger mechanisms of aging. Nat Genet 56:204–211. 10.1038/s41588-023-01627-0 38242993 10.1038/s41588-023-01627-0

[CR11] de Magalhães JP, Budovsky A, Lehmann G, Costa J, Li Y, Fraifeld V, Church GM (2009) The Human Ageing Genomic Resources: online databases and tools for biogerontologists. Aging Cell 8(1):65–72. 10.1111/j.1474-9726.2008.00442.x 18986374 10.1111/j.1474-9726.2008.00442.x PMC2635494

[CR12] de Magalhães JP, Raza A, Tacutu R (2024b) Human Ageing Genomic Resources: updates on key databases in ageing research. Nucleic Acids Res 52(D1):D900–D908. 10.1093/nar/gkad927 37933854 10.1093/nar/gkad927 PMC10767973

[CR13] Dönertaş HM, Fabian DK, Valenzuela MF, Partridge L, Thornton JM (2021) Common genetic associations between age-related diseases. Nat Aging 1:4. 10.1038/s43587-021-00051-5 10.1038/s43587-021-00051-5 PMC7610725 33959723

[CR14] Durinck S, Moreau Y, Kasprzyk A, Davis S, De Moor B, Brazma A, Huber W (2005) *BioMart* and Bioconductor: a powerful link between biological databases and microarray data analysis. Bioinformatics 21(16):3439–3440. 10.1093/bioinformatics/bti525 16082012 10.1093/bioinformatics/bti525

[CR15] Fernandes M, Wan C, Tacutu R, Barardo D, Rajput A, Wang J, Thoppil H, Thornton D, Yang C, Freitas A, de Magalhães JP (2016) Systematic analysis of the gerontome reveals links between aging and age-related diseases. Hum Mol Genet 25(21):4804–4818. 10.1093/hmg/ddw307 28175300 10.1093/hmg/ddw307 PMC5418736

[CR16] Fraser HC, Kuan V, Johnen R, Zwierzyna M, Hingorani AD, Beyer A, Partridge L (2022) Biological mechanisms of aging predict age-related disease co-occurrence in patients. Aging Cell 21(4):e13524. 10.1111/acel.13524 35259281 10.1111/acel.13524 PMC9009120

[CR17] Fu T, Yang Y-Q, Tang C-H, He P, Lei S-F (2024) Genetic effects and causal association analyses of 14 common conditions/diseases in multimorbidity patterns. PLoS ONE 19(5):e0300740. 10.1371/journal.pone.0300740 38753827 10.1371/journal.pone.0300740 PMC11098521

[CR18] Gazal S, Weissbrod O, Hormozdiari F, Dey K, Nasser J, Jagadeesh K, Weiner D, Shi H, Fulco C, O’Connor L, Pasaniuc B, Engreitz JM, Price AL (2022) Combining SNP-to-gene linking strategies to identify disease genes and assess disease omnigenicity. Nat Genet 54(6):827–836. 10.1038/s41588-022-01087-y 35668300 10.1038/s41588-022-01087-y PMC9894581

[CR19] Gruber HJ, Semeraro MD, Renner W, Herrmann M (2021) Telomeres and age-related diseases. Biomedicines 9(10):1335. 10.3390/biomedicines9101335 34680452 10.3390/biomedicines9101335 PMC8533433

[CR20] Guo J, Huang X, Dou L, Yan M, Shen T, Tang W, Li J (2022) Aging and aging-related diseases: from molecular mechanisms to interventions and treatments. Signal Transduct Target Ther 7:Article 391. 10.1038/s41392-022-01219-7 36522308 10.1038/s41392-022-01251-0 PMC9755275

[CR21] Horvath S, Raj K (2018) DNA methylation-based biomarkers and the epigenetic clock theory of ageing. Nat Rev Genet 19(6):371–384. 10.1038/s41576-018-0004-3 29643443 10.1038/s41576-018-0004-3

[CR22] Huber W, Carey VJ, Gentleman R, Anders S, Carlson M, Carvalho BS, Bravo HC, Davis S, Gatto L, Girke T, Gottardo R, Hahne F, Hansen KD, Irizarry RA, Lawrence M, Love MI, MacDonald J, Obenchain V, Oleś AK, Pagès H, Reyes A, Shannon P, Smyth GK, Tenenbaum D, Waldron L, Morgan M (2015) Orchestrating high-throughput genomic analysis with Bioconductor. Nat Methods 12(2):115–121. 10.1038/nmeth.3252 25633503 10.1038/nmeth.3252 PMC4509590

[CR23] Ismail AB, Balcıoğlu Ö, Özcem B, Ergoren MÇ (2024) APOE gene variation’s impact on cardiovascular health: a case-control study. Biomedicines 12(3):695. 10.3390/biomedicines12030695 38540308 10.3390/biomedicines12030695 PMC10968441

[CR24] Kanehisa M (2019) Toward understanding the origin and evolution of cellular organisms. Protein Sci 28(11):1947–1951. 10.1002/pro.3715 31441146 10.1002/pro.3715 PMC6798127

[CR25] Kanehisa M, Goto S (2000) KEGG: Kyoto encyclopedia of genes and genomes. Nucleic Acids Res 28(1):27–30. 10.1093/nar/28.1.27 10592173 10.1093/nar/28.1.27 PMC102409

[CR26] Kanehisa M, Furumichi M, Sato Y, Matsuura Y, Ishiguro-Watanabe M (2025) KEGG: biological systems database as a model of the real world. Nucleic Acids Res 53(D1):D672–D677. 10.1093/nar/gkae909 39417505 10.1093/nar/gkae909 PMC11701520

[CR27] Keshavarz M, Xie K, Schaaf K, Bano D, Ehninger D (2023) Targeting the “hallmarks of aging” to slow aging and treat age-related disease: fact or fiction? Mol Psychiatry 28(1):242–255. 10.1038/s41380-022-01680-x 35840801 10.1038/s41380-022-01680-x PMC9812785

[CR28] Kitsak M, Sharma A, Menche J, Guney E, Ghiassian SD, Loscalzo J, Barabási A-L (2016) Tissue specificity of human disease module. Sci Rep 6:35241. 10.1038/srep35241 27748412 10.1038/srep35241 PMC5066219

[CR29] Kodani N, Nakae J (2020) Tissue-specific metabolic regulation of FOXO-binding protein: FOXO does not act alone. Cells 9(3):702. 10.3390/cells9030702 32182991 10.3390/cells9030702 PMC7140670

[CR30] Koo HJ, Pan W (2024) Are trait-associated genes clustered together in a gene network? Genet Epidemiol 48(5):203–213. 10.1002/gepi.22557. 10.1002/gepi.22557 38472164

[CR31] Lawrence M, Huber W, Pagès H, Aboyoun P, Carlson M, Gentleman R, Morgan MT, Carey VJ (2013) Software for computing and annotating genomic ranges. PLoS Comput Biol 9(8):e1003118. 10.1371/journal.pcbi.1003118 23950696 10.1371/journal.pcbi.1003118 PMC3738458

[CR32] Lemaître G, Nogueira F, Aridas CK (2017) Imbalanced-learn: a *Python* toolbox to tackle the curse of imbalanced datasets in machine learning. J Mach Learn Res 18(17):1–5

[CR33] Li Y, Tian X, Luo J et al (2024) Molecular mechanisms of aging and anti-aging strategies. Cell Comm Sign. 10.1186/s12964-024-01663-1 10.1186/s12964-024-01663-1 PMC11118732 38790068

[CR34] Lonsdale J, Thomas J, Salvatore M et al (2013) The Genotype-Tissue Expression (GTEx) project. Nat Genet 45:580–585. 10.1038/ng.2653 23715323 10.1038/ng.2653 PMC4010069

[CR35] Lopera-Maya EA, Kurilshikov A, van der Graaf A, Hu S, Andreu-Sánchez S, Chen L et al (2022) Effect of host genetics on the gut microbiome in 7,738 participants of the Dutch Microbiome Project. Nat Genet 54:143–151. 10.1038/s41588-021-00992-y 35115690 10.1038/s41588-021-00992-y

[CR36] Mahley RW (2016) Apolipoprotein E: from cardiovascular disease to neurodegenerative disorders. J Mol Med 94(7):739–746. 10.1007/s00109-016-1427-y 27277824 10.1007/s00109-016-1427-y PMC4921111

[CR37] Martínez de Paz A, Josefowicz SZ (2021) Signaling-to-chromatin pathways in the immune system. Immunol Rev 300(1):37–53. 10.1111/imr.12955 33644906 10.1111/imr.12955 PMC8548991

[CR38] Martins R, Lithgow GJ, Link W (2016) Long live FOXO: unraveling the role of FOXO proteins in aging and longevity. Aging Cell 15(2):196–207. 10.1111/acel.12427 26643314 10.1111/acel.12427 PMC4783344

[CR39] Morris BJ, Willcox DC, Donlon TA, Willcox BJ (2015) FOXO3: a major gene for human longevity—a mini-review. Gerontology 61(6):515–525. 10.1159/000375235 25832544 10.1159/000375235 PMC5403515

[CR40] Oughtred R, Rust J, Chang C, Breitkreutz B-J, Stark C, Willems A, Boucher L, Leung G, Kolas N, Zhang F, Dolma S, Coulombe-Huntington J, Chatr-Aryamontri A, Dolinski K, Tyers M (2021) The *BioGRID* database: a comprehensive biomedical resource of curated protein, genetic, and chemical interactions. Protein Sci 30(1):187–200. 10.1002/pro.3978 33070389 10.1002/pro.3978 PMC7737760

[CR41] Paaby AB, Rockman MV (2013) The many faces of pleiotropy. Trends Genet 29(2):66–73. 10.1016/j.tig.2012.10.010 23140989 10.1016/j.tig.2012.10.010 PMC3558540

[CR42] Palmer D, Fabris F, Doherty A, Freitas AA, de Magalhães JP (2021) Ageing transcriptome meta-analysis reveals similarities and differences between key mammalian tissues. Aging (Albany NY) 13(3):3313–3341. 10.18632/aging.202648 33611312 10.18632/aging.202648 PMC7906136

[CR43] Pedregosa F, Varoquaux G, Gramfort A, Michel V, Thirion B, Grisel O et al (2011) Scikit-learn: machine learning in Python. J Mach Learn Res 12:2825–2830

[CR44] Raina P, Guinea R, Chatsirisupachai K, Lopes I, Farooq Z, Guinea C et al (2023) GeneFriends: gene co-expression databases and tools for humans and model organisms. Nucleic Acids Res. 10.1093/nar/gkac1031 36454018 10.1093/nar/gkac1031 PMC9825523

[CR45] Ren L-L, Miao H, Wang Y-N, Liu F, Li P, Zhao Y-Y (2023) *TGF-β* as a master regulator of aging-associated tissue fibrosis. Aging Dis 14(5):1633–1650. 10.14336/AD.2023.0222 37196129 10.14336/AD.2023.0222 PMC10529747

[CR46] Santos MA, Franco FN, Caldeira CA, de Araújo GR, Vieira A, Chaves MM, Lara RC (2021) Antioxidant effect of resveratrol: change in MAPK cell signaling pathway during the aging process. Arch Gerontol Geriatr 92:104266. 10.1016/j.archger.2020.104266 33070070 10.1016/j.archger.2020.104266

[CR47] Saul D, Kosinsky RL (2021) Epigenetics of aging and aging-associated diseases. Int J Mol Sci 22(1):401. 10.3390/ijms22010401 33401659 10.3390/ijms22010401 PMC7794926

[CR48] Shikov AE, Skitchenko RK, Predeus AV, Barbitoff YA (2020) Phenome-wide functional dissection of pleiotropic effects highlights key molecular pathways in human complex traits. Sci Rep 10:1037. 10.1038/s41598-020-58040-4 31974475 10.1038/s41598-020-58040-4 PMC6978431

[CR49] Teulière J, Bernard C, Corel E, Lapointe F-J, Martens J, Lopez P, Bapteste E (2023) Network analyses unveil ageing-associated pathways evolutionarily conserved from fungi to animals. GeroScience 45(2):1059–1080. 10.1007/s11357-022-00704-2 36508078 10.1007/s11357-022-00704-2 PMC9886728

[CR50] Valdeolivas A, Tichit L, Navarro C, Perrin S, Odelin G, Levy N, Cau P, Remy E, Baudot A (2018) Random walk with restart on multiplex and heterogeneous biological networks. Bioinformatics 35(3):497–505. 10.1093/bioinformatics/bty637 10.1093/bioinformatics/bty637 30020411

[CR51] White S, Medvedovic M (2016a) KEGGlincs design and application: An R package for exploring relationships in biological pathways [version 1; not peer reviewed]. *F1000Research*. 10.7490/f1000research.1113436.1

[CR52] White S, Medvedovic M (2016b) *KEGGlincs:* Visualize all edges within a KEGG pathway and overlay LINCS data [option]. R package version 1.1.0. http://www.bioconductor.org/packages/KEGGlincs.

[CR53] Wolfson M, Budovsky A, Tacutu R, Fraifeld V (2009) The signaling hubs at the crossroad of longevity and age-related disease networks. Int J Biochem Cell Biol 41(3):516–520. 10.1016/j.biocel.2008.08.026 18793745 10.1016/j.biocel.2008.08.026

[CR54] Xu S, Hu E, Cai Y, Xie Z, Luo X, Zhan L, Tang W, Wang Q, Liu B, Wang R, Xie W, Wu T, Xie L, Yu G (2024) Using *clusterProfiler* to characterize multiomics data. Nat Protoc 19(11):3292–3320. 10.1038/s41596-024-01020-z 39019974 10.1038/s41596-024-01020-z

[CR55] Yang J, Huang T, Petraglia F, Long Q, Zhang B, Argmann C et al (2015) Synchronized age-related gene expression changes across multiple tissues in human and the link to complex diseases. Sci Rep 5:15145. 10.1038/srep15145 26477495 10.1038/srep15145 PMC4609956

[CR56] Yang J, Huang T, Song WM et al (2016) Discover the network mechanisms underlying the connections between aging and age-related diseases. Sci Rep 6:32566. 10.1038/srep32566 27582315 10.1038/srep32566 PMC5007654

[CR57] Yousefzadeh M, Henpita C, Vyas R, Soto-Palma C, Robbins P, Niedernhofer L (2021) DNA damage—how and why we age? Elife. 10.7554/eLife.62852 33512317 10.7554/eLife.62852 PMC7846274

[CR58] Yusufuddin M, Young N (2019) Aging and ischemic stroke. Aging (Albany NY) 11(9):2542–2544. 10.18632/aging.101931 31043575 10.18632/aging.101931 PMC6535078

[CR59] Zhang JD, Wiemann S (2009) KEGGgraph: a graph approach to KEGG PATHWAY in R and Bioconductor. Bioinformatics 25(11):1470–1471. 10.1093/bioinformatics/btp167 19307239 10.1093/bioinformatics/btp167 PMC2682514

