# Supplementary material for: Pleiotropy and disease interactors: the dual nature of genes linking ageing and ageing-related diseases
Source: Biogerontology. 2026 Apr 29;27(3):95. doi: 10.1007/s10522-026-10429-w (PMC13124933; doi:10.1007/s10522-026-10429-w)
Supplement: Supplementary file 1 — Supplementary file1 (PDF 7723 KB) [file 10522_2026_10429_MOESM1_ESM.pdf]

Supplementary Materials

The Dual Nature of Genes Linking Ageing and Ageing-related Diseases.

Ageing- and disease-related genes

ARDs Clusters (ARCs)

In our study, we worked with 57 age-related diseases (ARDs) previously identified by Dönertaş *et al*. (2021) from UK Biobank self-reported data. To facilitate interpretation and reduce redundancy between closely related conditions, these ARDs were organized into 8 non-overlapping ARCs. Each ARC groups diseases that share common physiological or clinical domains, providing a higher-level framework for analysis. This classification, shown in Supplementary Fig. 1, includes: 1) Hematological and dermatological disorders, 2) Immunological and systemic disorders, 3) Musculoskeletal and trauma-related diseases, 4) Neurological, psychiatric, and eye disorders, 5) Gastrointestinal and abdominal diseases, 6) Renal and urological diseases, 7) Endocrine and diabetes-related diseases, 8) Cardiovascular diseases.

Common Genes Between Ageing and ARCs

To characterize the functional composition of ageing-related gene sets, we performed Gene Ontology (GO) enrichment analyses for three groups: $GenAge_{Hum}$-exclusive genes (present

only in the human ageing database), $GenAge_{Mod}$-exclusive genes (identified only in model organisms), and their intersection (genes conserved across both sources). Supplementary Table 1 summarizes the complete enrichment results, while only the ten most significant GO terms per group are shown for visualization. $GenAge_{Hum}$-*exclusive* genes were most strongly enriched in regulation of primary metabolic process, regulation of RNA biosynthetic process, and DNA-templated transcription (*p_adj≤1.2e-20*). $GenAge_{Mod}$-*exclusive* genes, in contrast, showed enrichment for cytoplasmatic translation (*p_adj=8.75e-4*). The intersection set was predominantly enriched in regulation of response to stimulus (*p_adj=1.02e-17*) and intracellular singal transduction (*p=3.18e-16*)..

Supplementary Figs. 2–3 illustrate the shared genes between ARCs and the *GenAge* datasets. Supplementary Fig. 4 illustrates the distribution of ARC-Pleiotropy levels across ARCs. Most ARCs were dominated by genes with low pleiotropy (levels 1–3), indicating disease-specific genetic components, whereas immunological/systemic disorder genes exhibited the opposite trend, being largely concentrated in high *ARC-Pleiotropy* levels (4–6).

Supplementary Table 2 presents GO enrichment for high *ARC-pleiotrop*y genes, which were predominantly associated with immunological systemic disorders.

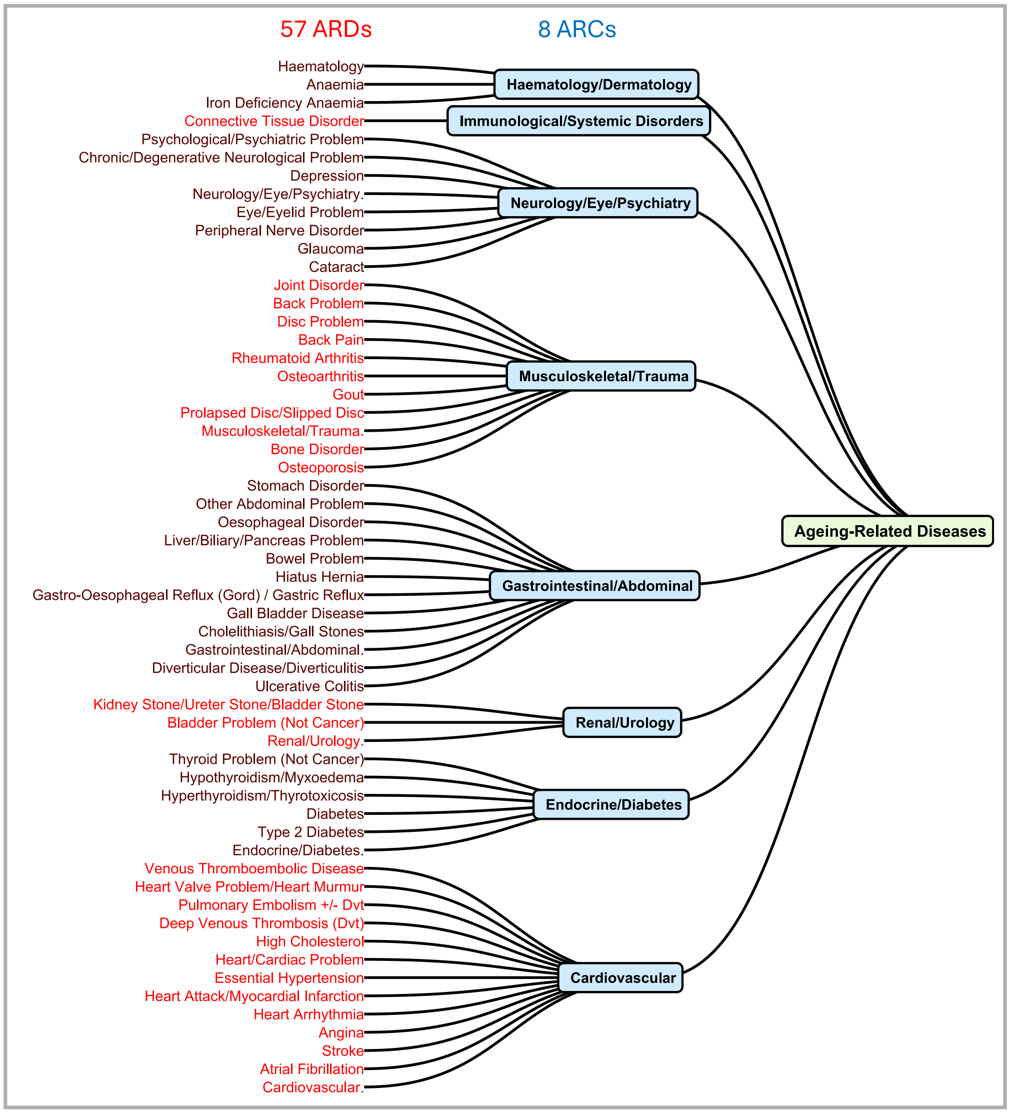

Supplementary Fig. 1: Classification of the 57 ARDs in 8 ARCs according to the UK Biobank's self-reported diseases grouping hierarchy.

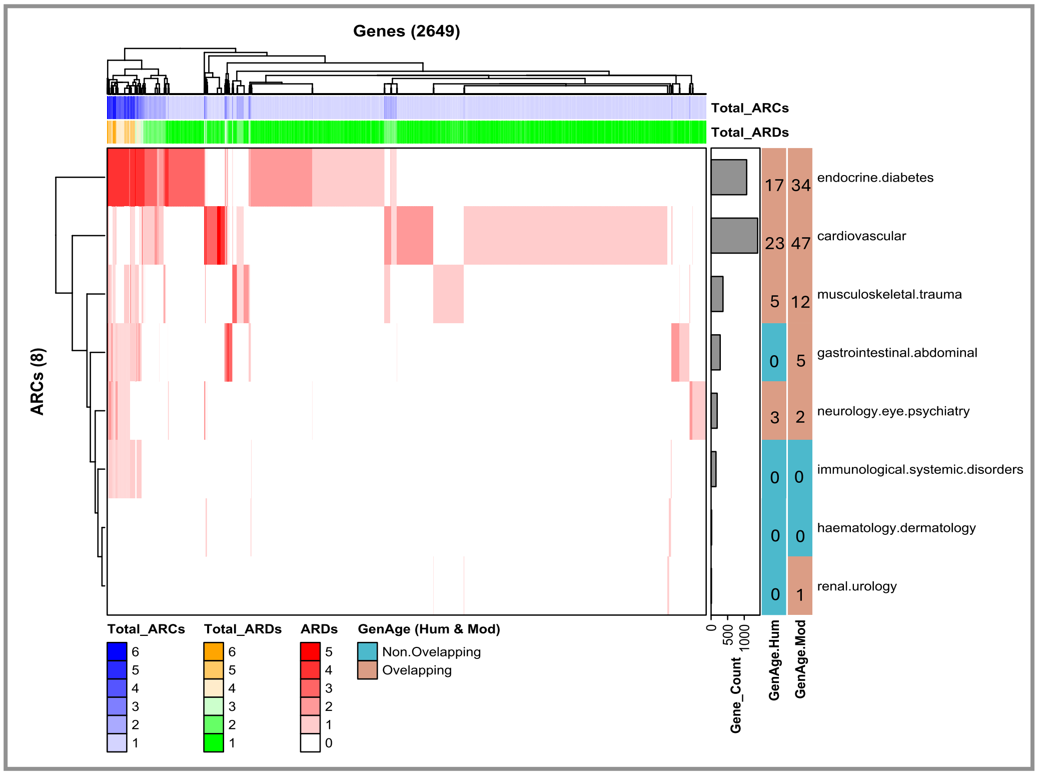

Supplementary Fig. 2: Genetic profiles of ARCs. Rows are ARCs *(i.e.*, a set of ARDs clustered by tissue) and columns are genes associated with them. A gradient from white to red is displayed, with white indicating that a gene does not interact with any diseases, and a more robust red colour representing a higher number of associated ARDs. Endocrine, cardiovascular, and gastrointestinal ARCs have a higher red colour, indicating a greater density of diseases affected by the same gene. On the right side of the graph, a histogram shows the number of genes per region, with cardiovascular having the most genes, followed by endocrine. Additionally, two extra bars indicate whether the genes within that ARC overlap with any of the two *GenAge*-associated groups or not, showing that gastrointestinal, immunological, haematological, and renal ARCs do not overlap with $GenAge_{Hum}$, whereas $GenAge_{Mod}$ overlapped with more ARC except immunological and haematological. At the top, several heatmaps and bars are displayed. The first bar represents the total number of ARDs for a particular gene, summing across all rows for the same column. The next bar indicates the total number of ARCs affected by the same gene, with a gradient from light blue to dark blue, with darker blue representing more ARCs.

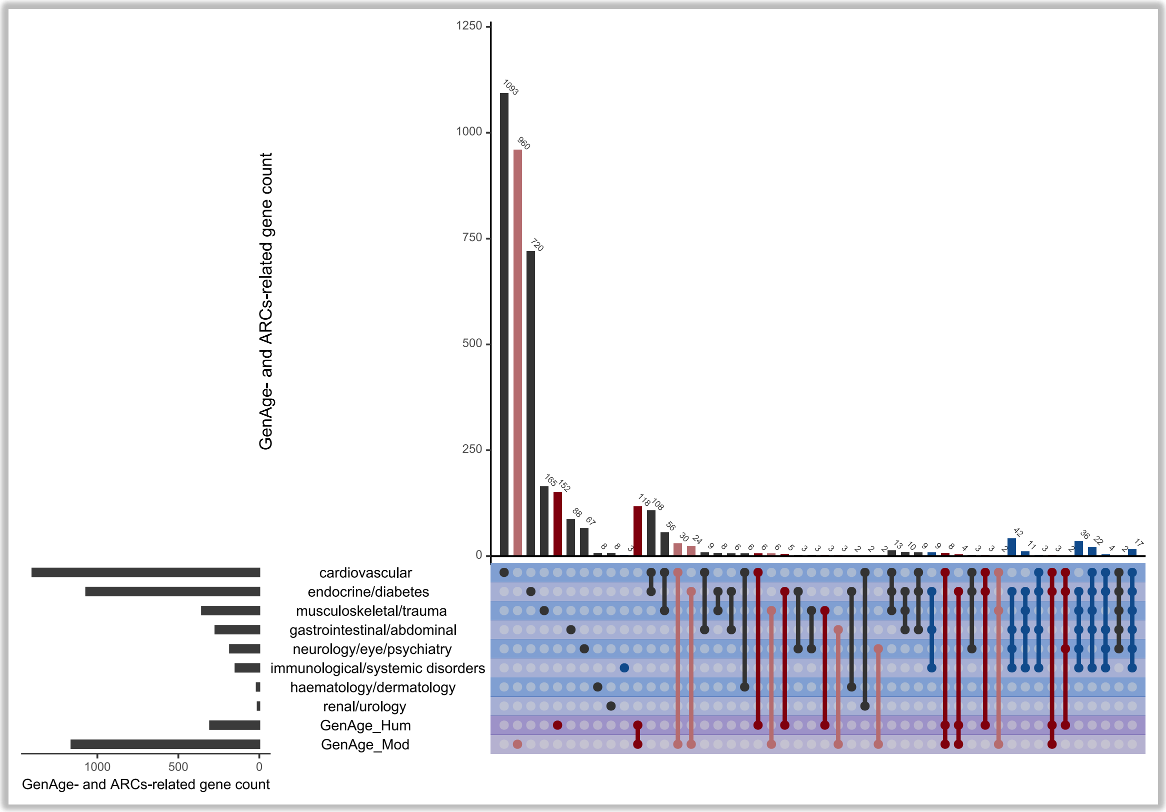

Supplementary Fig. 3: Overlap between genes associated with ageing and ARCs. A solid red colour was employed to denote genes linked to human ageing, while a lighter red indicates genes related to ageing in model organisms. An intense blue colour is utilised for lines corresponding to high *ARC-Pleiotropic* genes (immunological disorders genes), indicating the widely extended relationships between them and other ARCs. Dark-coloured lines represent the intersections between these ARCs-related genes when neither ageing- nor immune disease-related genes are involved. Purple rows represent the genes associated with any of the two categories of ageing-related genes. The rows in blue depict all the ARCs. The gene sets comprising only a single gene, though they may be associated with more than one ARC in some instances, were excluded for visual clarity. This exclusion does not importantly impact the relationships depicted in the graph, as these single-gene sets constitute a minority.

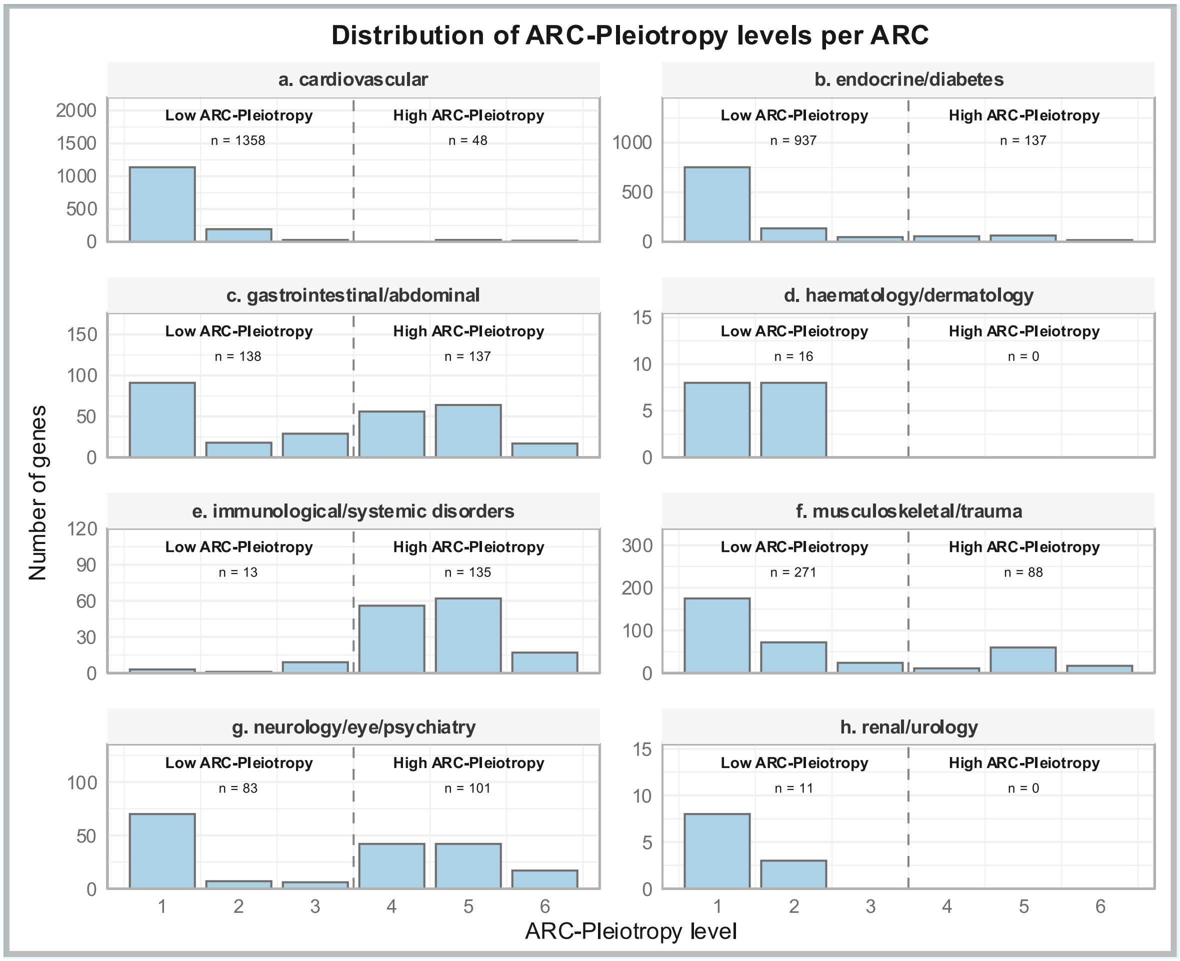

Supplementary Fig 4. Distribution of *ARC-Pleiotropy* levels across ARCs. Histograms show the number of genes associated with each ARC according to their *ARC-Pleiotropy* level (x-axis), defined as the number of distinct ARCs in which a gene shows a significant GWAS association through at least one age-related disease (ARD). Each subplot corresponds to one ARC (panels a–h), and bars represent counts of genes with *ARC-Pleiotropy* levels ranging from 1 to 6. A vertical dashed line separates genes with low *ARC-Pleiotropy* (levels 1–3) from those with high *ARC-Pleiotropy* (levels 4–6); the total number of genes in each half is indicated below the corresponding label. This division distinguishes genes restricted to one or few ARCs from those broadly shared across multiple ARC categories. Notably, immunological/systemic disorder genes show a predominance of high *ARC-Pleiotropy*, consistent with their wide involvement across ARCs and cross-system regulatory influence.

Supplementary Table 1: Top 10 biological process go terms associated with $GenAge_{Hum}$- and $GenAge_{Mod}$-exclusive genes as well as with the $GenAge_{Hum}$ - $GenAge_{Mod}$ intersection. Note how all these groups are mutually exclusive. The Term size is the number of genes associated with the GO term at the row. Query Size is the number of queried genes and intersection size is the number of queries genes associated with the GO term at hand.

***GenAge* Set**	**Term Name**	**Term id**	**Adjusted p_value**	**Term Size**	**Query Size**	**Intersection Size**
$GenAge_{Hum}$ Exclusive genes	regulation of primary metabolic process	GO:0080090	2.49e-23	535	167	131
	positive regulation of macromolecule metabolic process	GO:0010604	2.83e-23	391	167	111
	regulation of nucleobase-containing compound metabolic process	GO:0019219	3.51e-23	355	167	105
	regulation of macromolecule metabolic process	GO:0060255	3.51e-23	559	167	133
	regulation of RNA biosynthetic process	GO:2001141	3.29e-22	279	167	91
	nucleic acid metabolic process	GO:0090304	1.28e-21	452	167	117
	positive regulation of metabolic process	GO:0009893	2.76e-21	442	167	115
	regulation of cellular biosynthetic process	GO:0031326	3.21e-21	458	167	117
	cell differentiation	GO:0030154	8.91e-21	434	167	113
	DNA-templated transcription	GO:0006351	1.20e-20	288	167	90
$GenAge_{Mod}$ Exclusive genes	cytoplasmic translation	GO:0002181	8.75e-04	66	1031	65
Intersection between $GenAge_{Hum}$ and $GenAge_{Mod}$	regulation of response to stimulus	GO:0048583	1.02e-17	472	135	99
	cellular response to stimulus	GO:0051716	1.02e-17	715	135	121
	response to abiotic stimulus	GO:0009628	1.09e-16	204	135	62
	regulation of intracellular signal transduction	GO:1902531	1.09e-16	285	135	74
	positive regulation of metabolic process	GO:0009893	2.46e-16	442	135	93
	intracellular signal transduction	GO:0035556	3.18e-16	401	135	88
	cellular response to stress	GO:0033554	7.44e-16	341	135	80
	positive regulation of cellular metabolic process	GO:0031325	2.44e-15	372	135	83
	positive regulation of macromolecule metabolic process	GO:0010604	3.57e-15	391	135	85
	regulation of cell communication	GO:0010646	5.32e-15	437	135	90

Supplementary Table 2: Biological process GO terms associated with high *ARC-Pleiotropy* genes (immunological systemic disorders genes).

Term Name	Term id	Adjusted p_value	Term Size	Query Size	Intersection Size
nucleosome assembly	GO:0006334	1.39e-40	120	126	28
nucleosome organization	GO:0034728	8.42e-39	140	126	28
protein-DNA complex assembly	GO:0065004	7.08e-32	242	126	28
protein-DNA complex organization	GO:0071824	5.31e-31	262	126	28
chromatin remodeling	GO:0006338	1.11e-26	927	126	37
protein localization to CENP-A containing chromatin	GO:0061644	1.90e-18	18	126	10
protein localization to chromosome, centromeric region	GO:0071459	3.53e-14	41	126	10
epigenetic regulation of gene expression	GO:0040029	8.55e-14	295	126	17
negative regulation of megakaryocyte differentiation	GO:0045653	2.57e-13	20	126	8
protein-containing complex assembly	GO:0065003	2.02e-11	1701	126	30
megakaryocyte differentiation	GO:0030219	7.65e-09	67	126	8
innate immune response in mucosa	GO:0002227	3.61e-08	28	126	6
mucosal immune response	GO:0002385	3.21e-07	40	126	6
heterochromatin formation	GO:0031507	4.04e-07	161	126	9
negative regulation of gene expression, epigenetic	GO:0045814	1.70e-06	192	126	9
antibacterial humoral response	GO:0019731	1.73e-05	79	126	6
cellular component assembly	GO:0022607	4.07e-05	3205	126	30
T cell receptor signaling pathway	GO:0050852	4.72e-05	149	126	7
cellular component biogenesis	GO:0044085	2.07e-04	3481	126	30

Genetic Networks of Ageing and Ageing-related Diseases

Supplementary Table 3 summarizes the number of genes in each ARC network, the total gene–gene interactions, and the number of gene–ARD and gene–ARC associations. Supplementary Figs. 5, 7, 9, and 11 show the corresponding PPI, COX_{90}, COX_{95}, and $KEGG$ networks.

Supplementary Figs. 6, 8, 10, and 12 present heatmaps of network *shortest path distances* between genes and ARCs. Columns represent ARCs, while rows represent genes classified into four categories: *Disease-associated* (*i.e.*, ARC-related), $GenAge_{Hum}$, $GenAge_{Mod}$, and *Neighbours* (genes adjacent to ARC-associated genes in the network). Each heatmap cell encodes the shortest distance from a gene to the closest gene in the given ARC. Dark red indicates *Distance=0* (direct GWAS association), lighter red indicates *Distance=1* (neighbouring an ARC-associated gene, *i.e.*, *ARC-Interaction*), and white to blue shades denote longer distances. Distances greater than 1 were not considered *ARC-Interactions*, as more remote links may dilute biological relevance. Dark "*Inf*" values mark genes with no connection to the ARC.

Two annotation bars accompany each heatmap: the first indicates the number of ARCs a gene connects to at *Distance=1* (*i.e.*, its number of *ARC-Interactions*), and the second shows the mean distance of that gene to all ARCs.

PPI Network

Supplementary Fig. 5 displays the PPI network. Most genes cluster into a single large component, with only a small group of seven genes forming a separate cluster. Within this structure, $GenAge_{Hum}$-related genes occupy a central position with minimal dispersion,

whereas $GenAge_{Mod}$genes are also centrally located but more widely spread. Disease-associated genes are dispersed even further, spanning a larger portion of the network. Genes shared between $GenAge_{Hum}$ and $GenAge_{Mod}$ lie within the zone dominated by $GenAge_{Hum}$ genes but without forming a concentrated cluster. Interestingly, immunological disorder–associated genes, despite their extensive ARC connections, are positioned peripherally, often in intermediate locations rather than the core, with a small subset clustering at the upper right of the network.

Supplementary Fig. 6 presents network *shortest path distances* of genes in PPI relative to ARCs. Most genes are within two steps (*Distance* ≤ *2*) of multiple ARCs, except for immunological, renal, and haematological disorders, which tend to be the most distant (*Distance* ≥ *3*). Disease-associated genes cluster primarily with cardiovascular and endocrine disorders, with fewer links to other ARCs. Beyond their direct associations (*Distance=0*), these genes typically require two intermediate nodes to reach additional ARCs such as musculoskeletal, neurological, or gastrointestinal, indicating limited *ARC-Interactions* in a PPI context. A subset of disease-related genes remains confined to their own ARC, with no path to others (*Distance*=∞).

$GenAge_{Hum}$-associated genes show a stronger pattern of *ARC-Interactions* (*Distance=1*) than other groups: nearly half connect indirectly with two to six ARCs, especially cardiovascular and endocrine, and show relatively shorter distances to renal/urology. In contrast, $GenAge_{Mod}$ and Neighbour genes interact indirectly with only one or two ARCs, rarely exceeding three. The annotation bars for *ARC-Interactions* and mean distance visually emphasize the broader reach of $GenAge_{Hum}$ genes, which is statistically tested in the main text ("Disease Interactors") and Supplementary Section ("Proximity in Disease and Ageing Groups").

COX_{90} Network

Supplementary Fig. 7 shows the COX_{90} network, which is fragmented into several isolated clusters. The main component contains three to four large subclusters that host most ageing-related genes (both human and model-derived) and the majority of disease-associated genes, except those linked to immunological disorders or showing high *ARC-pleiotropy*. Within this component, $GenAge_{Hum}$ genes occupy central but dispersed positions, while $GenAge_{Mod}$ genes form a more compact cluster nearby. Disease-associated genes are broadly distributed, with many located close to $GenAge_{Mod}$. A smaller fraction of ageing- and disease-related genes, mainly from model organisms, appears in secondary subclusters. The second-largest cluster contains a dense module of immunological and highly *ARC-Pleiotropic* genes, forming a strongly coexpressed but isolated group. Additional minor clusters contain only a few ageing- or disease-related genes. Genes shared between $GenAge_{Hum}$ and $GenAge_{Mod}$ are present within the main component but are less discernible due to local crowding. The overall cluster-based organization means that genes in different components cannot be reached from one another, resulting in infinite network distances.

Supplementary Fig. 8 illustrates *network shortest path distances* in COX_{90}. Compared with PPI, disease-associated genes display more frequent *ARC-Interactions*, reflected by widespread light-red signals across multiple ARCs. These indirect associations extend to most systems but are weaker for renal, haematological, and immunological ARCs. Differences between $GenAge_{Hum}$, $GenAge_{Mod}$, disease-related, and neighbour genes are subtle, although *Neighbouring* genes show slightly broader *ARC-Interaction* patterns. This is partly reflected in the *ARC-Interactions* and mean-distance annotation bars. Even for distant ARCs such as renal,

haematological, and immunological, shortest paths were often two steps, indicating closer associations than in PPI or $KEGG$. Nonetheless, some *Disease*-related genes, and to a lesser extent ageing and neighbour genes, remain isolated in separate clusters, leaving them at infinite distance from other ARCs.

COX_{95} Network

Supplementary Fig. 9 shows the COX_{95} network, which is fragmented into fewer but smaller clusters compared to COX_{90}. The main cluster encompasses most $GenAge_{Hum}$, $GenAge_{Mod}$, and disease-associated genes, except those related to immunological disorders or displaying high *ARC-pleiotropy*. Within this cluster, $GenAge_{Hum}$-related genes are sparse and scattered, in contrast to the more evenly distributed $GenAge_{Mod}$ genes. Disease-associated genes, by comparison, are more centrally positioned. A secondary cluster is divided into two modules connected by a few bridging genes, including some $GenAge_{Mod}$. Additional small clusters contain few disease-associated genes, with one cluster dominated by highly *ARC-Pleiotropic* genes (*e.g.*, immunologic systemic disorder-related genes). This latter cluster lacks both low *ARC-Pleiotropy* genes and any *GenAge* genes.

Supplementary Fig. 10 depicts *shortest path distances* in the COX_{95} network. Compared to COX_{90}, *GenAge* genes show fewer *ARC-Interactions*, with only ~10% connecting indirectly to multiple ARCs. This network also contains the smallest number of *GenAge-associated* genes among all networks. Most $GenAge_{Hum}$- and $GenAge_{Mod}$-related genes remain distant, with only a minority linking to more than one ARC at *Distance=1*. Neighbour genes typically connect at *Distance 1–2*, while disease-associated genes also remain within one or two steps of their own ARC but less often reach others. Some ARCs (particularly renal, haematological, and

immunological) are largely disconnected in this COX_{95} network. Nevertheless, 10–30% of each gene group still establishes at least some indirect connections, indicating that a small subset bridges across otherwise isolated clusters.

$KEGG$ Network

Supplementary Fig. 11 shows the $KEGG$ signalling network, which resembles PPI in being dominated by a single large cluster that integrates all human $KEGG$ pathways. A few minor clusters exist, usually containing background genes with only one or two disease- or $GenAge_{Mod}$-related genes. Within the main cluster, $GenAge_{Hum}$ and $GenAge_{Mod}$ genes are broadly dispersed but remain relatively central, avoiding the periphery of the network. Disease-associated genes follow a similar distribution. In contrast, immunological disorder–related genes cluster at the network's edges, grouped within specific cascades. These clusters predominantly contain high *ARC-pleiotropy* genes but also include some *GenAge* and low *ARC-Pleiotropy* disease-related genes nearby. Genes shared between $GenAge_{Hum}$ and $GenAge_{Mod}$ also map to the main cluster, scattered yet centrally located, underscoring their overlapping but distinct contributions.

Supplementary Fig. 12 depicts gene–ARC *shortest path distances* in $KEGG$. Disease-associated genes connect directly (*Distance=0*) to their primary ARC but are typically two or more steps away from other ARCs. $GenAge_{Hum}$-related genes show most *ARC-Interactions* with cardiovascular and endocrine disorders, with smaller proportions linking to gastrointestinal and musculoskeletal ARCs. $GenAge_{Mod}$ genes display an intermediate profile between $GenAge_{Hum}$ and neighbour genes, with indirect links concentrated in cardiovascular and endocrine ARCs. Neighbour genes usually interact with only one or two ARCs, again mostly

cardiovascular and endocrine. As in PPI, immunological, renal, and haematological ARCs remain the least connected, reflecting their relative isolation in the signalling network.

Topological Properties of the Networks

Supplementary Table 4 summarizes network centrality and connectivity measures across gene groups.

Degree and betweenness centrality were consistently higher for $GenAge_{Hum}$ genes in PPI and $KEGG$, reflecting their central positions, although this likely reflects research bias toward well-studied genes. $GenAge_{Mod}$ genes also showed elevated degree values but to a lesser extent; in COX networks they even surpassed $GenAge_{Hum}$ in degree, though with lower betweenness. By contrast, disease-related genes occupied intermediate positions, with lower centrality than both *GenAge* groups but higher than genes not associated with ageing or disease. Immunological disorder genes, despite their high *ARC-pleiotropy*, displayed the lowest degree and betweenness across all networks.

In closeness centrality, GenAge-associated genes tended to hold lower values relative to the remaining groups, while immunological disorder and background genes were slightly higher. Across networks, COX_{95} yielded the highest closeness, while PPI and $KEGG$ were lowest.

The *clustering coefficient* varied strongly by network: COX_{90} showed the highest clustering (68-74%), followed by COX_{95} (61-68%), PPI (14-26%), and finally $KEGG$ (3-24%). Across groups, background genes had the strongest clustering, $GenAge_{Hum}$ genes the lowest, and $GenAge_{Mod}$ slightly higher. Immunological disorder-related genes showed similar clustering to other groups in PPI and COX, but reached the lowest values in $KEGG$.

The *percentage of disease-related neighbours* was ~9% for $GenAge_{Mod}$ and slightly higher for $GenAge_{Hum}$, comparable to other and disease-related genes in most networks. Immunological disorder genes typically showed medium to low number of disease neighbours: ~15% in PPI, only 1% in $KEGG$, but high values in COX networks (47% in COX_{90}, and 67% in COX_{95}). As shown in Supplementary Figs. 7 and 9, this apparent connectivity mainly reflected clustering among immunological genes themselves, rather than broad links to other disease groups.

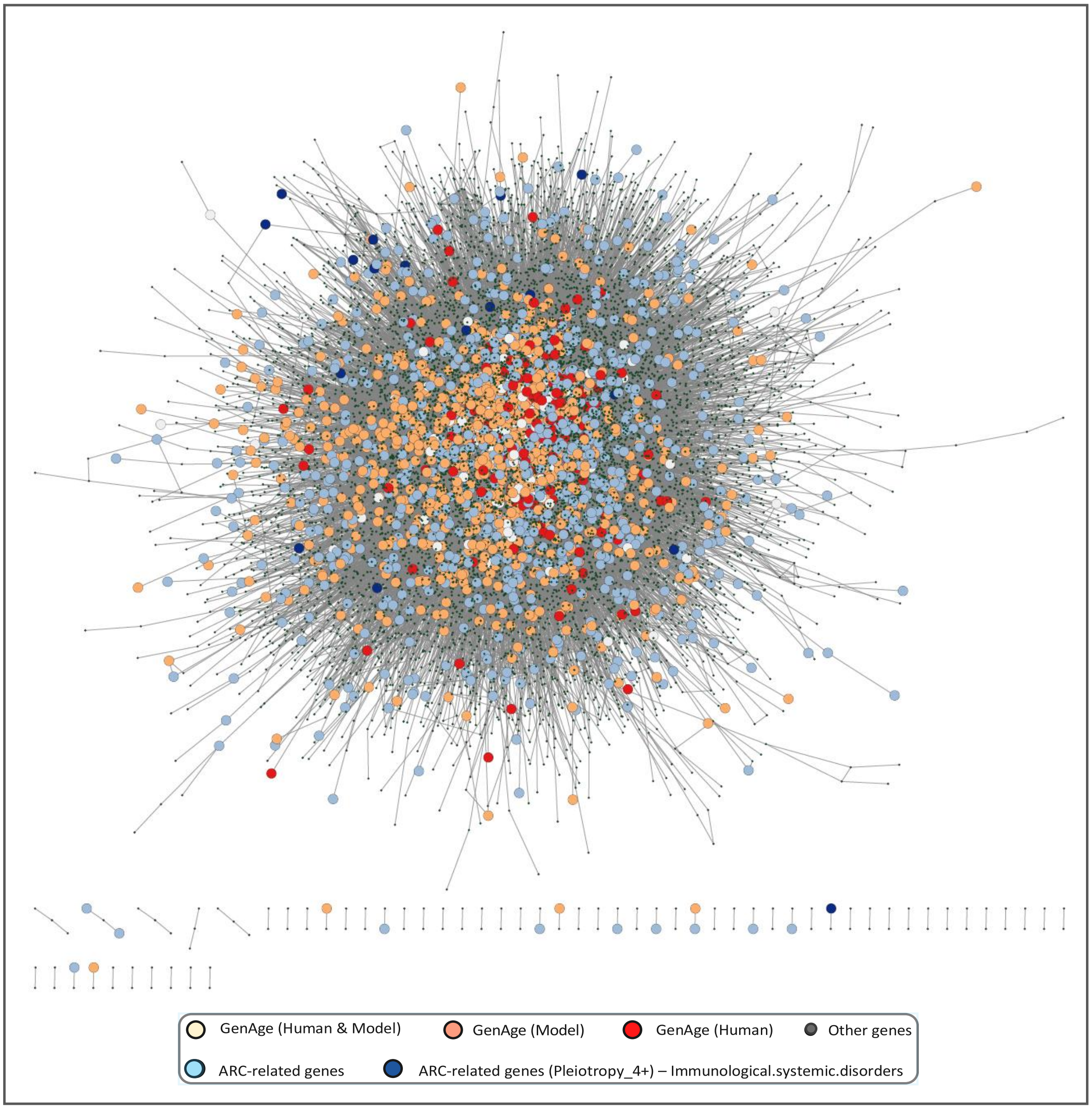

Supplementary Fig. 5: PPI network. Genes are color-coded: *Disease* (light blue), $GenAge_{Hum}$ (red), $GenAge_{Mod}$ (orange), $GenAge_{Hum}$ & $GenAge_{Mod}$ (light yellow), and Immunological Sytemic Disorders genes (strong blue). Background genes have a dark grey shade and smaller nodes. Only ageing- and *Disease*-related genes that belong to the PPI network are displayed.

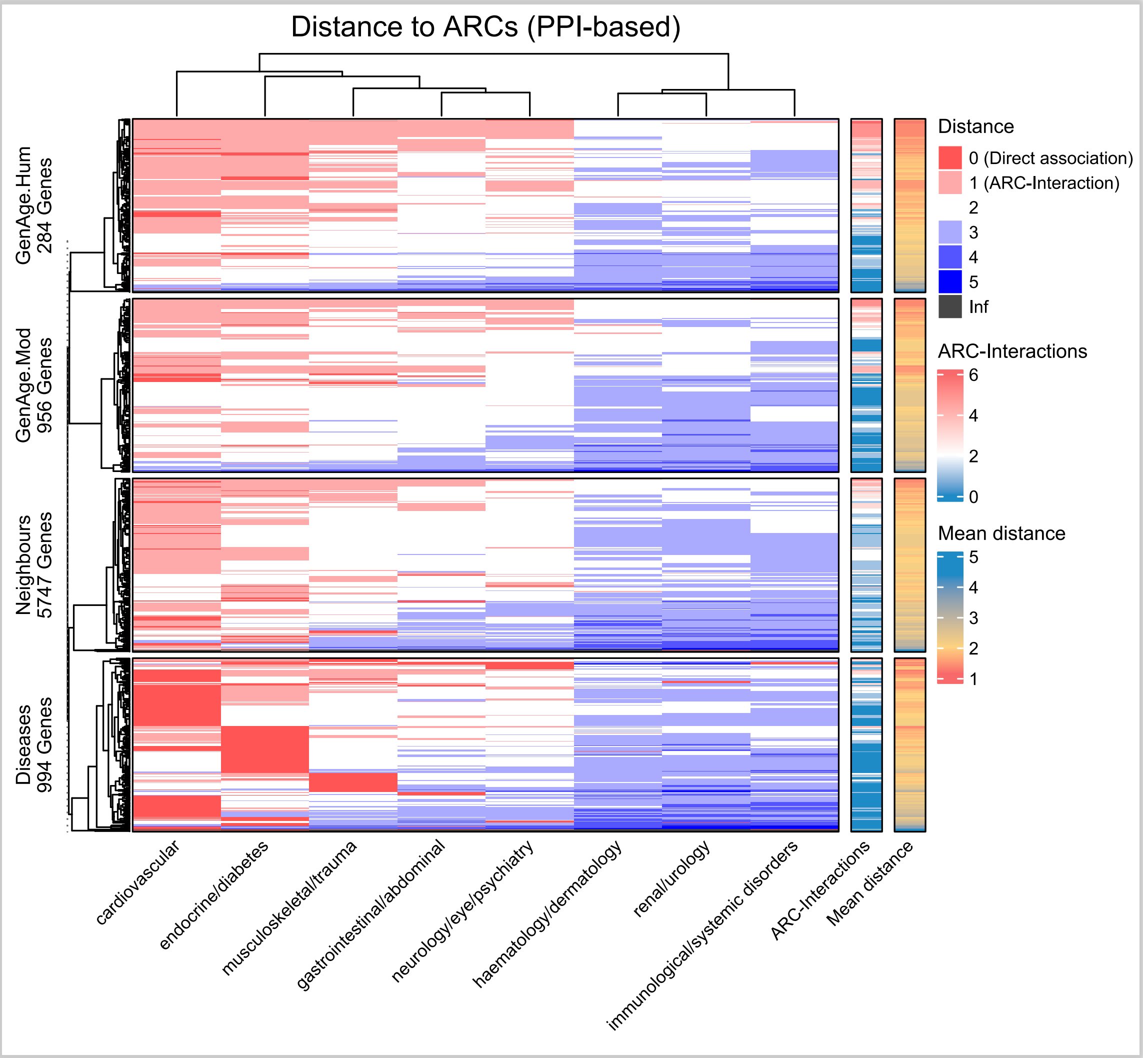

Supplementary Fig. 6: Genetic *shortest path distances* at the *PPI* network. Heatmap representation of genetic distances between a genes and ARCs, as determined by their position within the *PPI* gene interaction network. Full explanation of this and the remaining similar figures is provided in the main text at the beginning of this section (second paragraph of section - Genetic Networks).

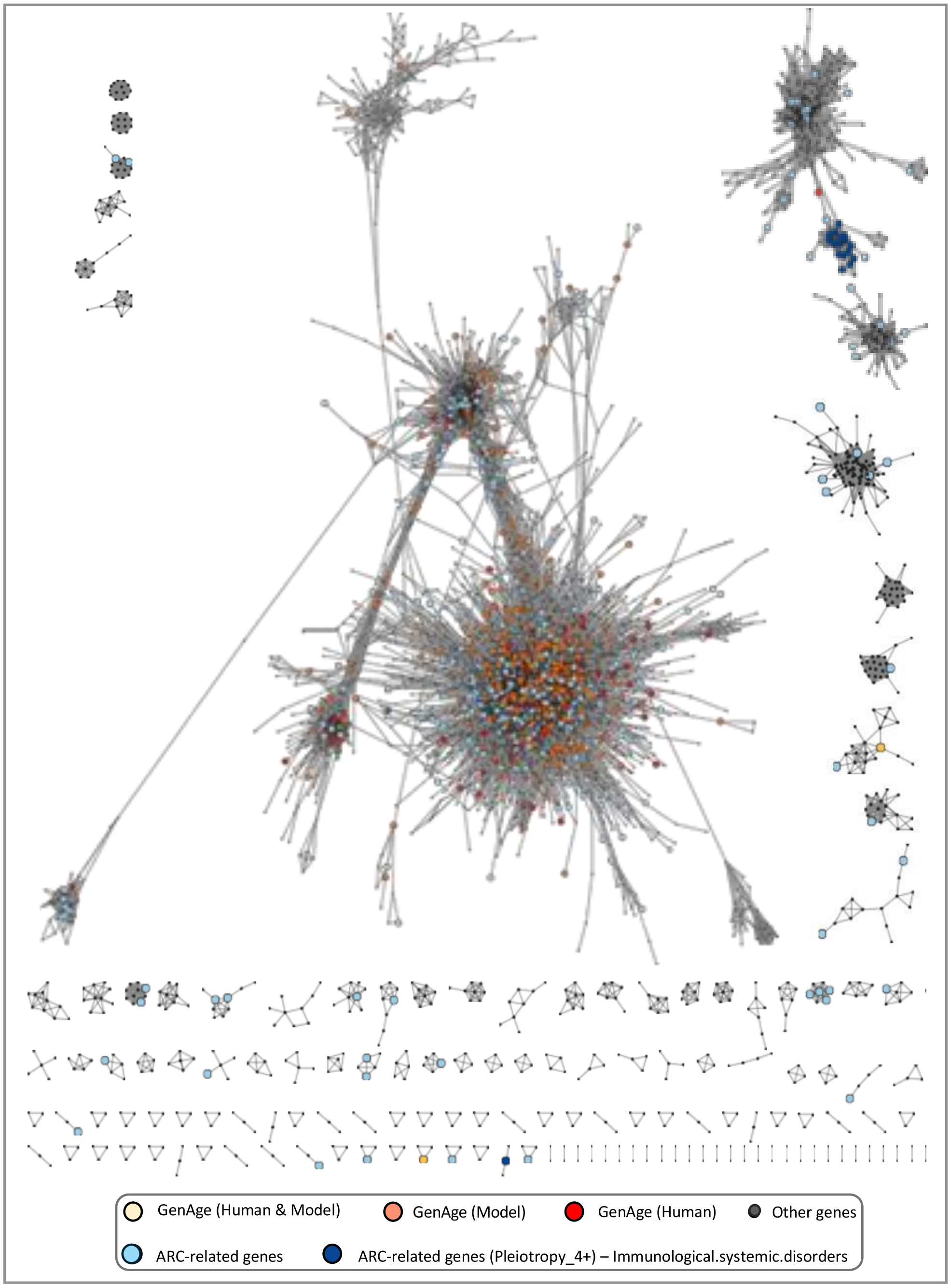

Supplementary Fig. 7: COX_{90} Network. Genes are color-coded: *Disease* (light blue), $GenAge_{Hum}$ (red), $GenAge_{Mod}$ (orange), $GenAge_{Hum}$ & $GenAge_{Mod}$ (light yellow), and Immunological Sytemic Disorders genes (strong blue). Background genes have a dark grey shade and smaller nodes. Only ageing- and *Disease*-related genes that belong to this coexpression network are displayed.

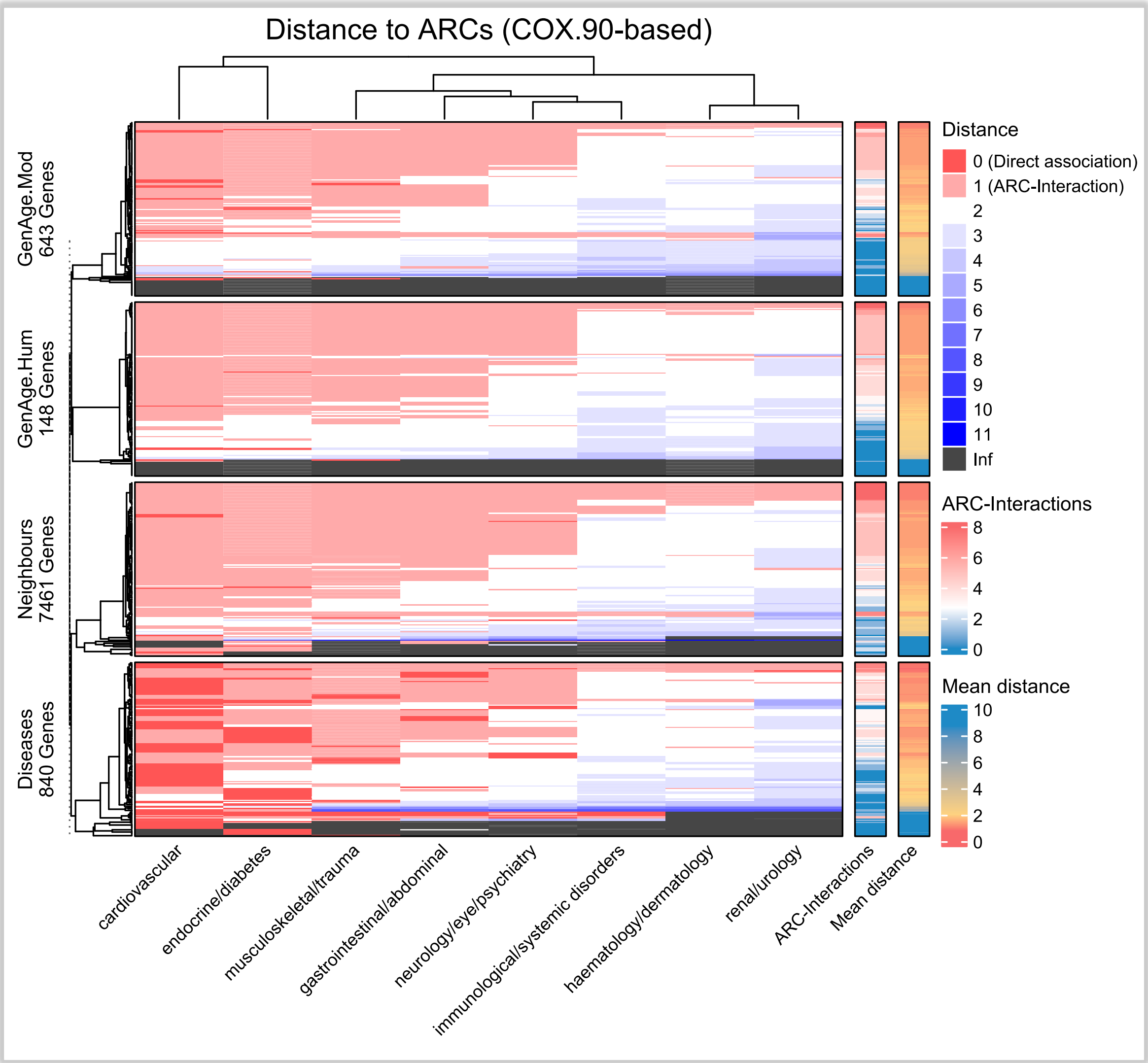

Supplementary Fig. 8: Genetic *shortest path distances* at the COX_{90} network. Heatmap representation of genetic distances between a genes and ARCs, as determined by their position within the the COX_{90} gene interaction network. Full explanation of this and the remaining similar figures is provided in the main text at the bebanging of this section (second paragraph of section - Genetic Networks).

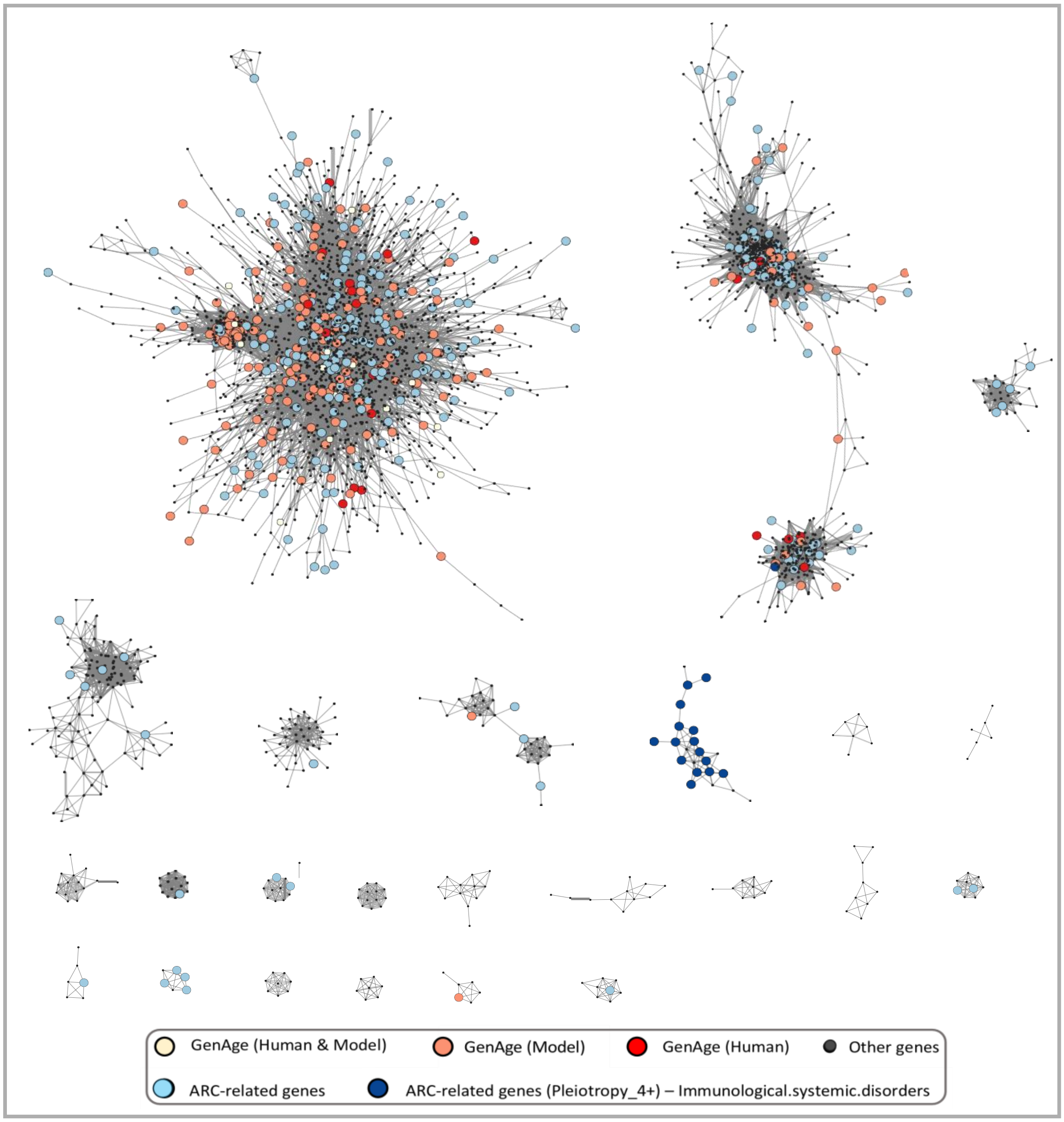

Supplementary Fig. 9: COX_{95} Network. Genes are color-coded: *Disease* (light blue), $GenAge_{Hum}$ (red), $GenAge_{Mod}$ (orange), $GenAge_{Hum}$ & $GenAge_{Mod}$ (light yellow), and Immunological Sytemic Disorders genes (strong blue). Background genes have a dark grey shade and smaller nodes. Only ageing- and *Disease*-related genes that belong to this coexpression network are displayed. Clusters of genes with less than five genes were excluded for ease of visualization.

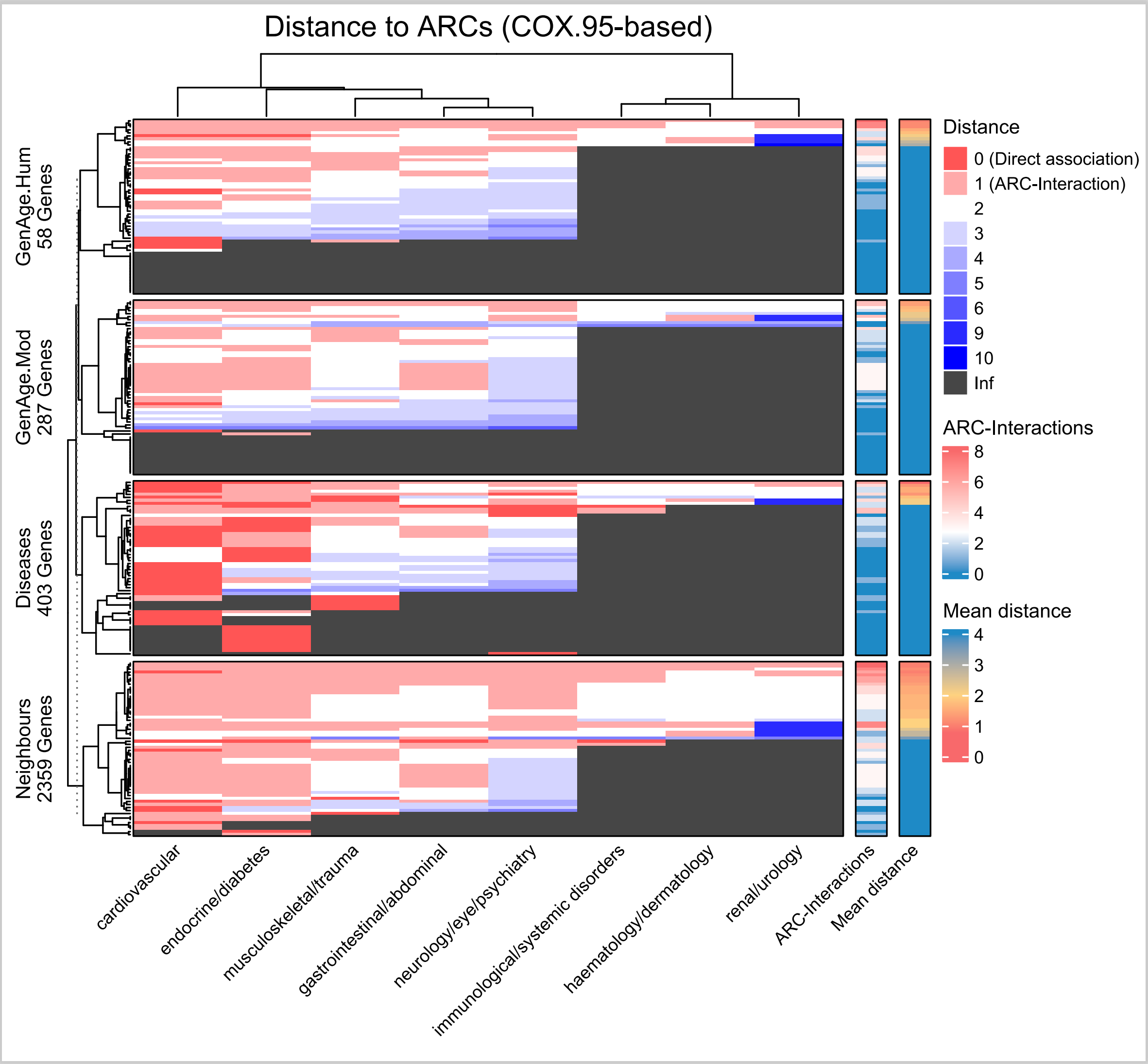

Supplementary Fig. 10: Genetic *shortest path distances* at the COX_{95} network. Heatmap representation of genetic distances between a genes and ARCs, as determined by their position within the COX_{95} network. Full explanation of this and the remaining similar figures is provided in the main text at the bebanging of this section (second paragraph of section - Genetic Networks).

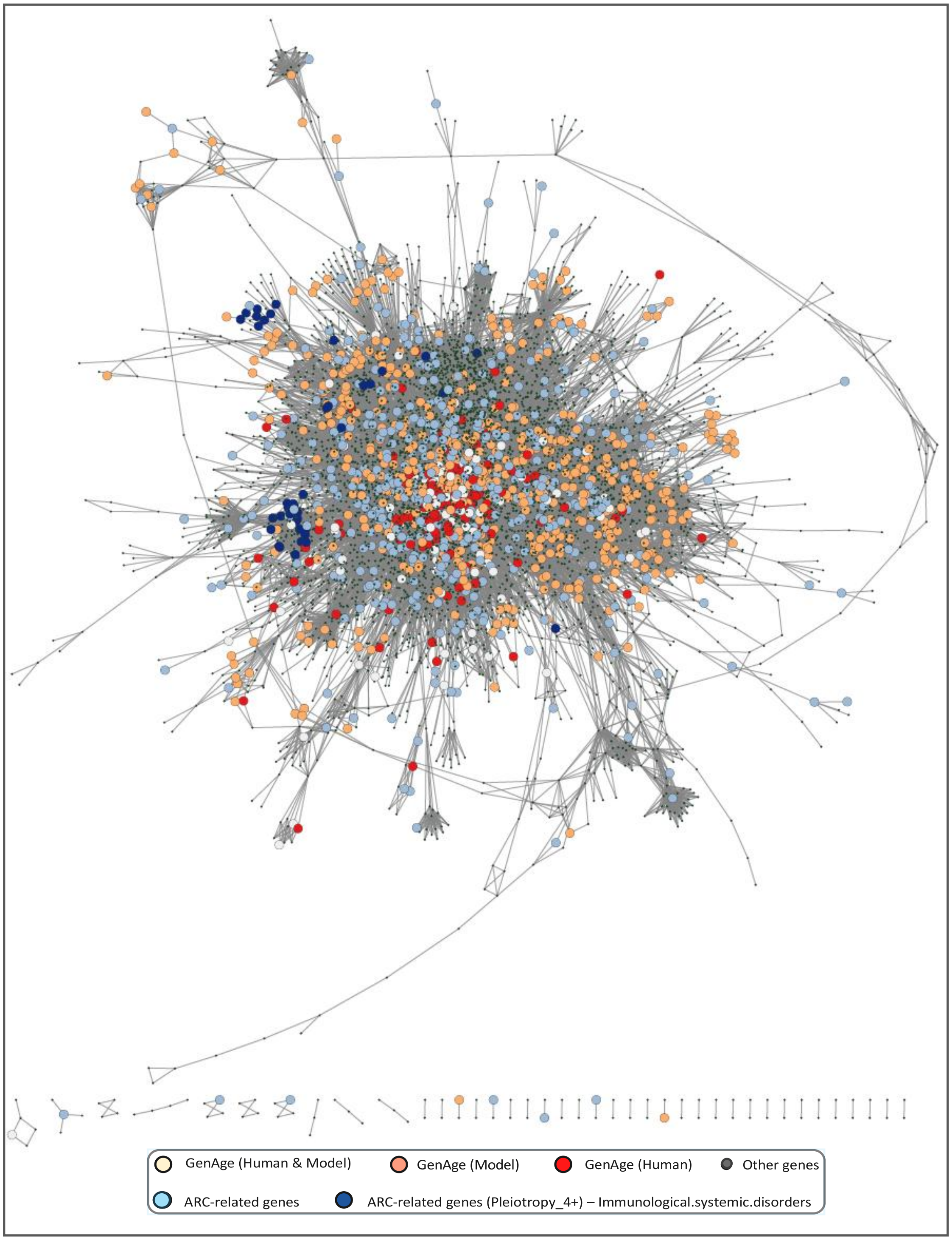

Supplementary Fig. 11: $KEGG$ Network. Genes are color-coded: *Disease* (light blue), $GenAge_{Hum}$ (red), $GenAge_{Mod}$ (orange), $GenAge_{Hum}$ & $GenAge_{Mod}$ (light yellow), and Immunological Sytemic Disorders genes (strong blue). Background genes have a dark grey shade and smaller nodes. Only ageing- and *Disease*-related genes that belong to this coexpression network are displayed.

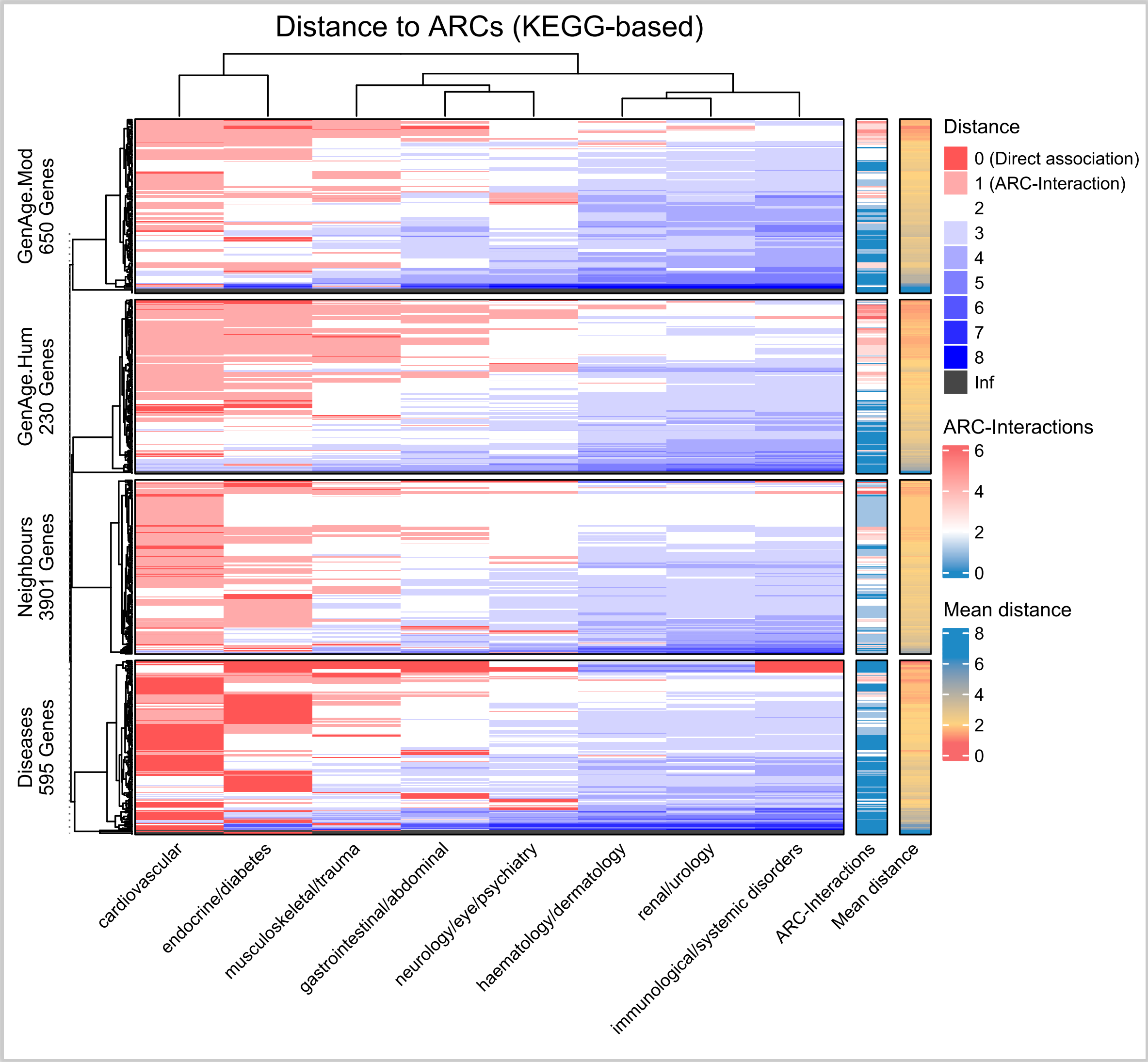

Supplementary Fig. 12: Genetic *shortest path distances* at the $KEGG$ network. Heatmap representation of genetic distances between a genes and ARCs, as determined by their position within the $KEGG$ network. A full explanation of this and the remaining similar figures is provided in the main text at the beginning of this section (second paragraph of section - ARC Genetic Networks).

Supplementary Table 3. Measures of genes edges at the PPI, $KEGG$, COX_{90}, and COX_{95} networks.

Network	Disease -related genes	Gene-Gene Network genes	Disease genes ∪ Gene-Gene Network	Disease genes ∩ Gene-Gene Network genes	Gene-Gene edges	ARDs Connected to Gene-Gene Network (Out of 57)	ARCs Connected to Gene-Gene Network (Out of 8)
PPI	2649	11,837	13,492	994	93,086	51	8
COX_{90}	2649	11,136	11,945	840	1,463,273	48	8
COX_{95}	2649	5,106	7,352	403	141,865	48	8
$KEGG$	2649	6,320	8,374	595	59,856	49	8

Supplementary Table 4: Topological properties of gene groups across the ARC-related networks. The sets of genes are associated with $GenAge_{Hum}$, $GenAge_{Mod}$, Diseases (ARC-related genes) and high ARC-Pleiotropy. The group Others represent random genes outside any of these categories. For each group of genes, the mean value of Degree, Betweenness and Closeness Centralities are displayed. Similarly, the mean value of Clustering Coefficient and the mean Percentage of the Reference gene's neighbours associated with at least one Disease are presented.

Network	Genes	Degree Centrality	Betweenness Centrality	Closeness Centrality	Clustering Coefficient	Percentage of Neighbour Genes related with *Diseases*
PPI	Diseases	16	12726	1%	24%	8%
	$GenAge_{Hum}$	72	131980	1%	14%	9%
	$GenAge_{Mod}$	37	48235	1%	24%	9%
	High ARC-Pleiotropy	4	4617	11%	22%	15%
	Others	13	10830	1%	26%	8%
COX_{90}	Diseases	282	14340	10%	72%	14%
	$GenAge_{Hum}$	293	33539	8%	68%	8%
	$GenAge_{Mod}$	348	13385	5%	69%	8%
	High ARC-Pleiotropy	48	729	14%	71%	47%
	Others	255	10704	14%	74%	7%
COX_{95}	Diseases	52	1703	24%	63%	25%
	$GenAge_{Hum}$	45	1410	24%	61%	9%
	$GenAge_{Mod}$	62	2166	18%	61%	8%
	High ARC-Pleiotropy	21	25	16%	63%	67%
	Others	55	1314	31%	68%	6%
$KEGG$	Diseases	21	9518	1%	22%	8%
	$GenAge_{Hum}$	48	60105	0%	17%	10%
	$GenAge_{Mod}$	33	28050	1%	25%	9%
	High ARC-Pleiotropy	8	1567	0%	3%	1%
	Others	16	7069	1%	24%	9%

ARC interactors

Explanation

Supplementary Fig. 13 illustrates the concepts of *ARC-Pleiotropy* and *ARC-Interactions*.

ARC-Pleiotropy is defined as the ARCs (clusters of ARDs) with which a gene is directly associated through GWAS, meaning it has at least one significant gene–phenotype association with a ARD belonging to each ARC. A gene is thus considered linked to an ARC if it shows a GWAS association with one or more diseases included in that ARC. For example, if a gene is associated with hypertension and stroke (both within the Cardiovascular ARC) and with arthritis (within the Musculoskeletal ARC), its *ARC-Pleiotropy=2*.

In contrast, *ARC-Interactors* are genes that are indirectly connected to one or more ARCs through their network neighbours (*i.e.*, in PPI, $KEGG$, COX_{90} or COX_{95}) that *are* GWAS-associated with diseases belonging to those ARCs. Thus, a gene has *ARC-Interactions=n* when it connects, through first-order interactions, to genes associated with diseases in *n* different ARCs.

Methodologically, *ARC-Pleiotropy* quantifies direct GWAS-based influence on diseases within clusters, whereas *ARC-Interactivity* captures indirect influence inferred from the topological connectivity of the genetic networks

Correlation between *ARC-Pleiotropy* and *ARC-Interactions*

Supplementary Fig. 14 depicts the joint distribution of *ARC-Pleiotropy* and *ARC-Interactions* across networks. The first column of all panels highlights genes with no direct ARC associations (*ARC-Pleiotropy=0*) but with varying degrees of *ARC-Interactions*. This group contained the

majority of genes across all networks. In PPI and $KEGG$, however, the number of genes systematically declined as *ARC-Interaction* levels increased at *ARC-Pleiotropy=0*, whereas the COX networks deviated from this trend by maintaining a relatively consistent gene density across several *ARC-Interaction* levels. In fact, COX_{90} exhibited its highest density of genes at *ARC-Pleiotropy=0* with *ARC-Interactor*=5, while the other networks peaked at lower *ARC-Interactor* levels (1–2).

Across networks, a general trend was observed, where the number of genes decreased with increasing ARC interactions, regardless of ARC pleiotropy. This decrease was even more pronounced as ARC pleiotropy increased, regardless of the level of ARC interactions. The COX networks maintained a broader spectrum of *ARC-Interactions* within the lower *ARC-Pleiotropy* range (0–2), often reaching six or more interaction levels. By contrast, PPI and $KEGG$ networks showed an early reduction in *ARC-Interactions*, especially beyond *ARC-Pleiotropy* ≥ 3, where genes rarely exceeded one indirect connection. This trend was most stringent in $KEGG$, where genes with *ARC-Pleiotropy=6* typically lacked ARC-Interactions altogether. In contrast, the COX networks allowed genes with high *ARC-Pleiotropy* (5–6) to retain relatively elevated *ARC-Interactions* (6–7).

Overall, the correlation between *ARC-Pleiotropy* and *ARC-Interactions* was consistently negative across networks. $KEGG$ (–0.26) and PPI (–0.27) showed the strongest inverse relationships, while COX_{95} (–0.12) and COX_{90} (–0.11) displayed weaker but still negative correlations. Thus, genes with low *ARC-Pleiotropy* were more likely to exhibit higher levels of *ARC-Interactions*, especially in PPI and $KEGG$, while this trade-off was less pronounced in the COX networks.

Permutation tests for *ARC-Interactors*

We next evaluated whether ageing-related genes, disease-associated genes, and high *ARC-pleiotropy* genes (immunological disorders) exhibit non-random numbers of *ARC-interactors* across our four networks: PPI, COX_{90}, COX_{95}, and $KEGG$. In each case, observed values were compared against null distributions generated from 10,000 size-matched random samples of all genes present in the respective network (Supplementary Fig. 15 and Table 1 of the main text).

In both PPI and $KEGG$ (Supplementary Fig. 15 a-h), $GenAge_{Hum}$ showed the highest numbers of *ARC-interactors (2.11 vs. 0.79 null in* PPI*; 1.93 vs. 1.05 null in* $KEGG$*; both p_adj=1.6e-3*), indicating the strongest integration within ARC-related molecular interactions. $GenAge_{Mod}$ also exceeded random expectations but to a lesser extent (*1.4 vs. 0.79 null in* PPI*; 1.4 vs. 1.05 null in* $KEGG$*; both p_adj=1.6e-3*). Disease-associated genes had the lowest number of *ARC-Interactors* (*0.57 vs 0.79 null in* PPI*; 0.64 vs 1.05 null in* $KEGG$*; both p_adj=1.6e-3*), while high *ARC-pleiotropy* immunological genes showed slight or no depletion (*0.12 vs 0.78 null in* PPI*, ns; 0.03 vs 1.04 null in* $KEGG$*, ns*).

In *coexpression networks* (Supplementary Fig. 15 i-p), *ARC-interactor* counts were generally lower than PPI and $KEGG$ but followed consistent trends. In the COX_{95} network, ageing-related genes showed no significant difference with random expectation ($GenAge_{Hum}$*=1.29 vs. 1.29 null* ns; $GenAge_{Mod}$*=1.44 vs. 1.29 null, ns*), disease-related genes were depleted (*0.94 vs. 1.29 null, p_adj=3.2e-3*), and high *ARC-Pleiotropy* genes were not significantly different from random (0.9 vs. 1.3 null, ns). In the COX_{90} network, connectivity increased across all groups: $GenAge_{Mod}$ genes reached the highest values overall (*3.12 vs. 2.61 null, p_adj=1.6e-3*), followed by $GenAge_{Hum}$ which lacked significant difference with the null (*2.7 vs. 2.61 null, ns*).

Disease-related genes remained with a significantly lower value or ARC-Interactors (*2.18 vs. 2.61 null, p_adj=1.6e-3*), and high *ARC-Pleiotropy* genes were further below random (*0.83 vs. 2.61 null, p=4.8e-3*).

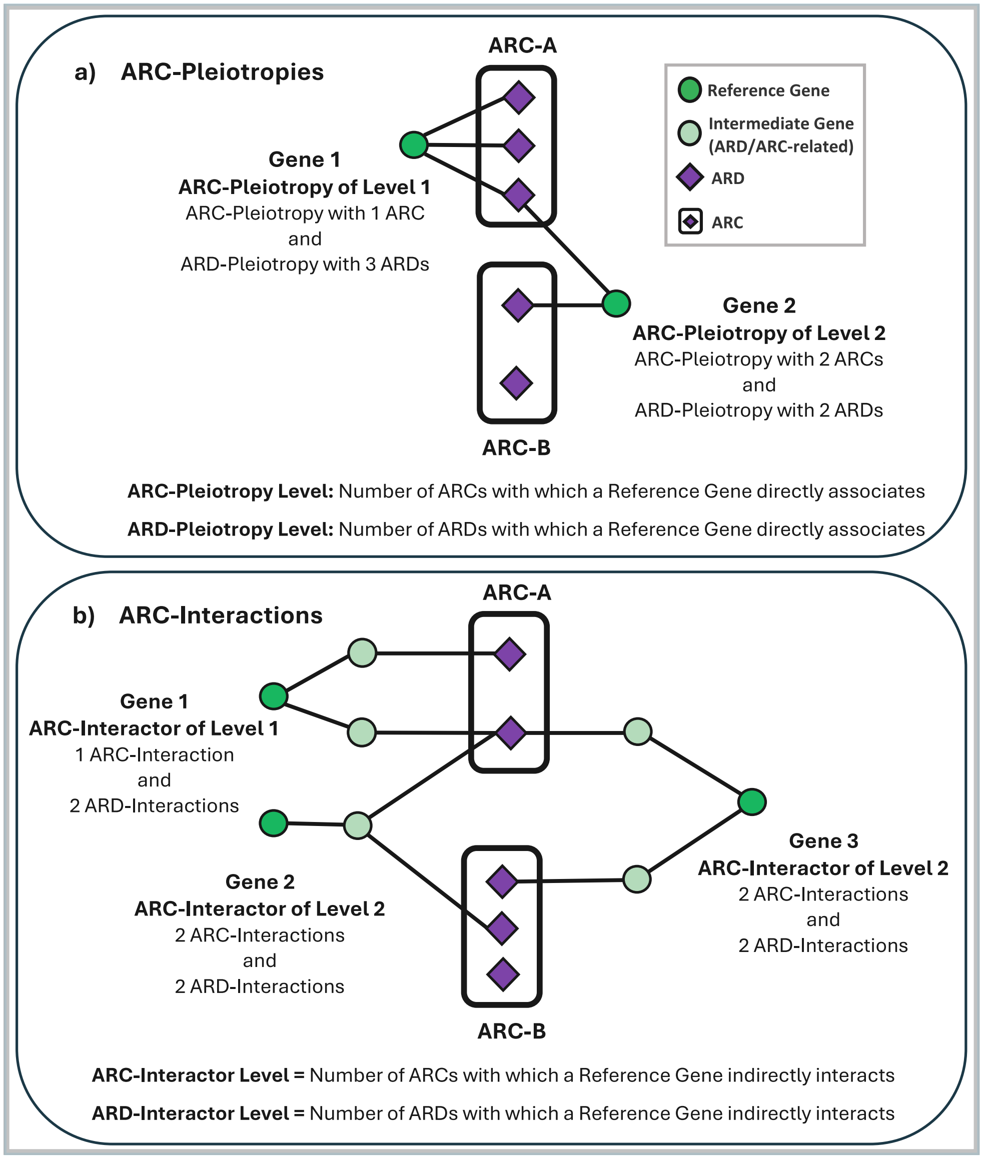

Supplementary Fig. 13: *ARC-Pleiotropy* and *ARC-Interactors*. A gene can influence multiple ARDs or ARCs through direct association or indirect interaction. The darker green circle represents the reference gene, lighter green circles indicate intermediate genes and purple diamonds symbolize ARDs. **a.** *ARC-Pleiotropy*. The reference gene is directly associated with different ARDs, which, in turn, associates the genes with the ARCs to which the ARDs belong. *ARC-Pleiotropy* is categorized as *ARD-Pleiotropy* or *ARC-Pleiotropy,* and a level value is assigned depending on the number of associated ARDs or ARCs, respectively. Gene 1 indicates that a Gene associated with multiple ARDs does not necessarily associate with multiple ARCs. Gene 2 shows an association with 2 ARCs through an association with 2 ARDs. **b.** *ARC-Interactor*. The reference gene indirectly interacts with different ARDs, which indirectly associates the genes with the ARCs to which the ARDs belong. The indirect interaction is mediated through Gene-Gene interaction with an ARD/ARC-related gene. Interactors are categorized as ARD-*Interactor* or ARC-*Interactor,* and a level value is assigned depending on the number of indirectly associated ARDs or ARCs, respectively. Gene 1 indirectly interacts with 1 ARC despite being associated with 2 ARDs. Gene 2 interacts with an *ARC-Pleiotropic* Gene of Level 2, leading to indirect interactions with 2 ARDs and ARCs. Gene 3 indirectly interacts with 2 ARDs in two different ARCs. Node designs were inspired by (Weighill *et al.* 2019).

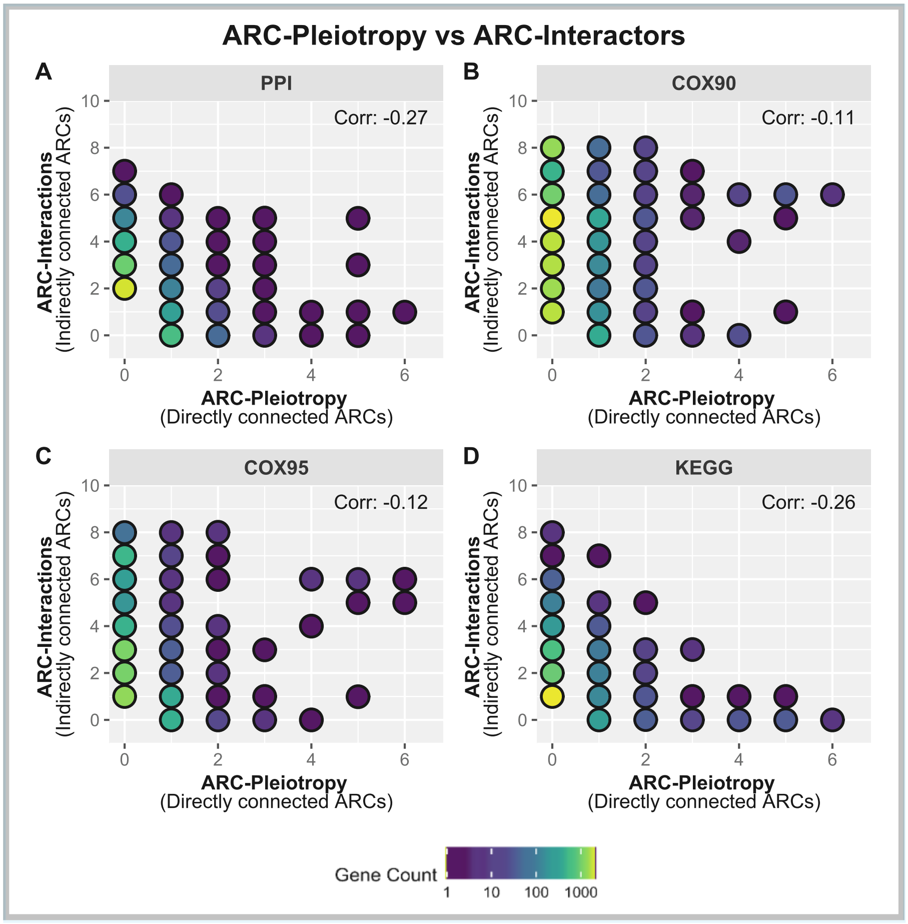

Supplementary Fig. 14: Correlation between *ARC-Pleiotropy* and *ARC-Interactors*. Direct and Indirect connections to ARCs (*i.e.*, *ARC-Pleiotropies* and *ARC-Interactions*, respectively) across all genes on the six different networks used in this study. Each point in this chart, denoted by a circle, represents a unique pairing of *ARC-Pleiotropy* and *ARC-Interactions* values, with the circle's colour indicating the number of genes associated to each point. Correlation scores are written on each panel. **a**. PPI network. **b.** ARC network. **c.** COX_{90} network. **d.** $KEGG$.

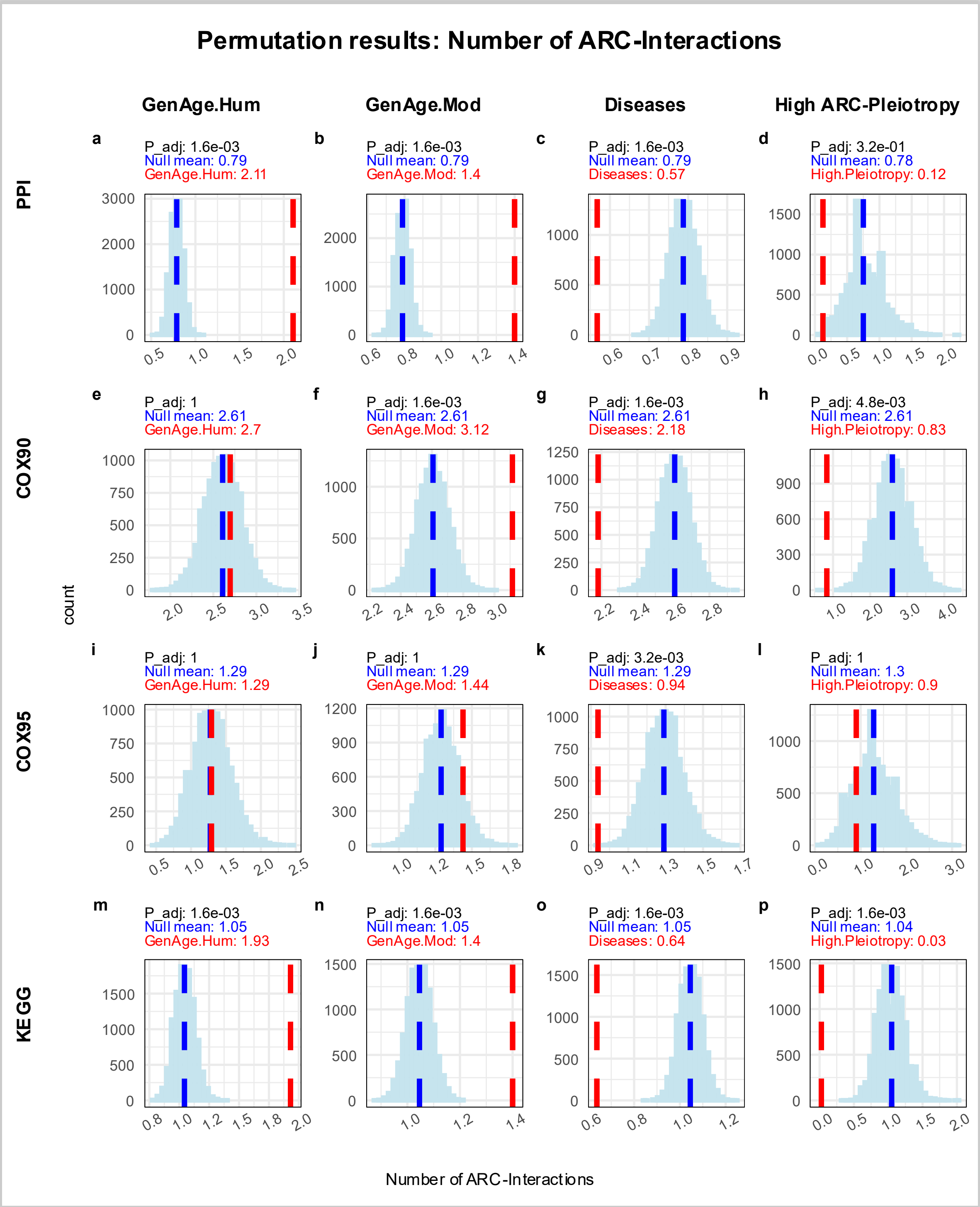

Supplementary Fig. 15: Histograms of null distributions of mean numbers of *ARC-interactors* obtained from 10,000 permutations of all genes present in each network. Blue dashed lines indicate the null mean, while red dashed lines mark the observed values for each gene set. Results are shown for (**a–d**) PPI, (**e–h**) COX_{90}, (**i–l**) COX_{95}, and (**m–p**) $KEGG$ networks, across $GenAge_{Hum}$, $GenAge_{Mod}$, disease-associated, and high *ARC-Pleiotropy* immunological disorder genes.

Topological association of genes with ARCs (description)

Given that our analysis of *ARC-interactors* already revealed that $GenAge_{Hum}$ tend to interact more strongly to ARCs in PPI and $KEGG$ networks, whereas high *ARC-Pleiotropy* genes showed weaker-than-expected interacticity under random expectation, we next asked whether other quantitative measures could capture these differences more systematically. To this end, we developed two complementary frameworks: *Shortest path distance* between reference genes and ARC/ARD-associated modules, and *Random Walk with Restart (RWR)*, which integrates local and global topology to estimate the probability of reaching disease modules. By applying permutation tests, we evaluated whether these metrics reveal non-random patterns in the relationship between ageing-related genes, highly *ARC-Pleiotropic* genes, and ARDs.

Shortest path distance to Diseases (*Proximity* Analysis)

To quantify how ageing-related genes are positioned relative to ARC- and ARD-associated genes within molecular networks, we computed their genetic distance based on the minimum number of edges connecting them. For any two genes, the *shortest_path_distance* was defined as the smallest number of edges along the shortest path between the reference gene and a target gene (Supplementary Fig. 16). Under this definition, *Shortest_path_distance*=0 when the two genes are identical and becomes infinite when no path connects them. In the context of ARCs or ARDs, this metric represents the shortest path from a given reference gene to any gene associated with the target ARC or ARD.

Because some genes are disconnected in sparse networks (yielding infinite distances) and others coincide with the reference gene (distance=0), we employed a bounded *Proximity*

transformation to normalize values and allow averaging across all pairwise relationships. We defined *Proximity* as:

$$Proximity = \frac{1}{Shortest.distance + 1}$$

This formulation ensures that *Proximity* values remain within the [0, 1] interval—equal to 1 when two genes overlap (distance=0) and approaching 0 as distance increases toward infinity. Thus, disconnected gene pairs contribute a *Proximity* of 0, while self-connections contribute 1.

For each gene, two complementary *Proximity* metrics were computed relative to each ARC or ARD:

- ***Shortest_Path_Proximity***: the maximum *Proximity* value, corresponding to the shortest path (*i.e.*, nearest connection) between the reference gene and any ARC/ARD-associated gene (Supplementary Fig. 16).
- ***Average_Path_Proximity***: the mean of *Proximity* values between the reference gene and all genes associated with the ARC/ARD of interest, where disconnected pairs (infinite distance) contribute zero (Supplementary Fig. 16).

For each gene, *Proximity* values were first calculated independently for each of the ARCs, using either *Shortest_Path_Proximity* or *Average_Path_Proximity* depending on the metric. These *Proximity* values were then averaged across all ARCs to obtain a single representative measure of how closely each gene is positioned, on average, to ARDs clusters within the network.

To express results in intuitive distance units, this mean *Proximity* was then converted back into its equivalent distance using the inverse transformation:

$$Distance = \frac{1}{Proximity + 1}$$

where *Proximity* corresponds to the mean *Shortest_Path_Proximity* or mean *Average_Path_Proximity* value. This approach ensures that disconnected genes (with infinite distances) contribute a *Proximity* of 0, directly connected or overlapping genes contribute a *Proximity* of 1, and the final mean genetic distance to ARCs reflects the typical network separation between each gene and the overall architecture of ARCs.

Random Walk with Restart

RWR is a network diffusion algorithm commonly used in systems biology to capture global connectivity patterns. It simulates a random walker that moves across the network by following edges, but at each step there is a fixed probability of returning to the starting node (the "restart"). After many iterations, this process converges to a stationary distribution that reflects how strongly each node is connected to the starting set. Unlike shortest-path metrics, which emphasize the single most direct connection, RWR integrates both local associations and long-range topology, making it well-suited for identifying functional associations between genes and disease modules.

In conceptual terms, the RWR score can be understood as a measure of global accessibility (Supplementary Fig. 17). If a gene attains a high value, it means that when random walks are initiated from multiple ARCs, the walker repeatedly returns to that gene, reflecting its privileged position of connectivity, surrounded by neighbors that are also disease-related. Conversely, a low score indicates that although the gene may lie close to a specific module, its reach is limited and it is seldom visited when the diffusion process explores the entire network. In other words, a high RWR score implies that the gene functions as a global point of convergence in

the network architecture, whereas a low RWR score characterizes it as a peripheral or locally confined node.

We implemented RWR on both individual networks (*Monoplex*) and their combination (*Multiplex* network) integrating all four layers (PPI, COX_{90}, COX_{95}, $KEGG$ pathways). In each case, the analysis was restricted to the genes present in the corresponding network. For the *Multiplex*, the union of genes across all four layers was used; genes absent from a particular layer were represented as isolated genes to maintain consistency across layers.

RWR was performed using the R package *RandomWalkRestartMH* (Valdeolivas et al., 2018). *Multiplex* objects were constructed and normalized supra-adjacency matrices were computed, providing the transition probabilities used for propagation. The restart probability was fixed at r=0.7, meaning that at each iteration the walker had a 70% chance of returning to the seed set and a 30% chance of moving to neighbouring genes. In *Multiplex* analyses, inter-layer transitions were controlled by the parameter τ, which was defined as τ=[1,1,1,1] to assign equal weight to all four layers.

Independent runs were performed for each seed set. Two types of seed sets were defined: (i) ARD-level, with 57 runs, each using the GWAS-associated genes of one ARD, and (ii) ARC-level, with 8 runs, each using the genes associated with one ARC. This resulted in 65 runs per network, repeated across the four *Monoplex* networks (260 runs) and once for the *Multiplex* (65 runs), for a total of 325 independent RWR analyses.

For each run, an RWR score was obtained for every gene in the network relative to the corresponding seed set. Scores were standardized as Z-scores within each seed set. From these, we generated (i) association tables reporting RWR scores and ranks for all genes, (ii) per-gene summaries of mean association across ARDs and ARCs, and (iii) top-ARD and top-ARC

assignments identifying the ARD or ARC with the maximum score for each gene. These outputs were subsequently used in downstream analyses, including comparisons across networks and integration with Machine Learning (ML) classifiers.

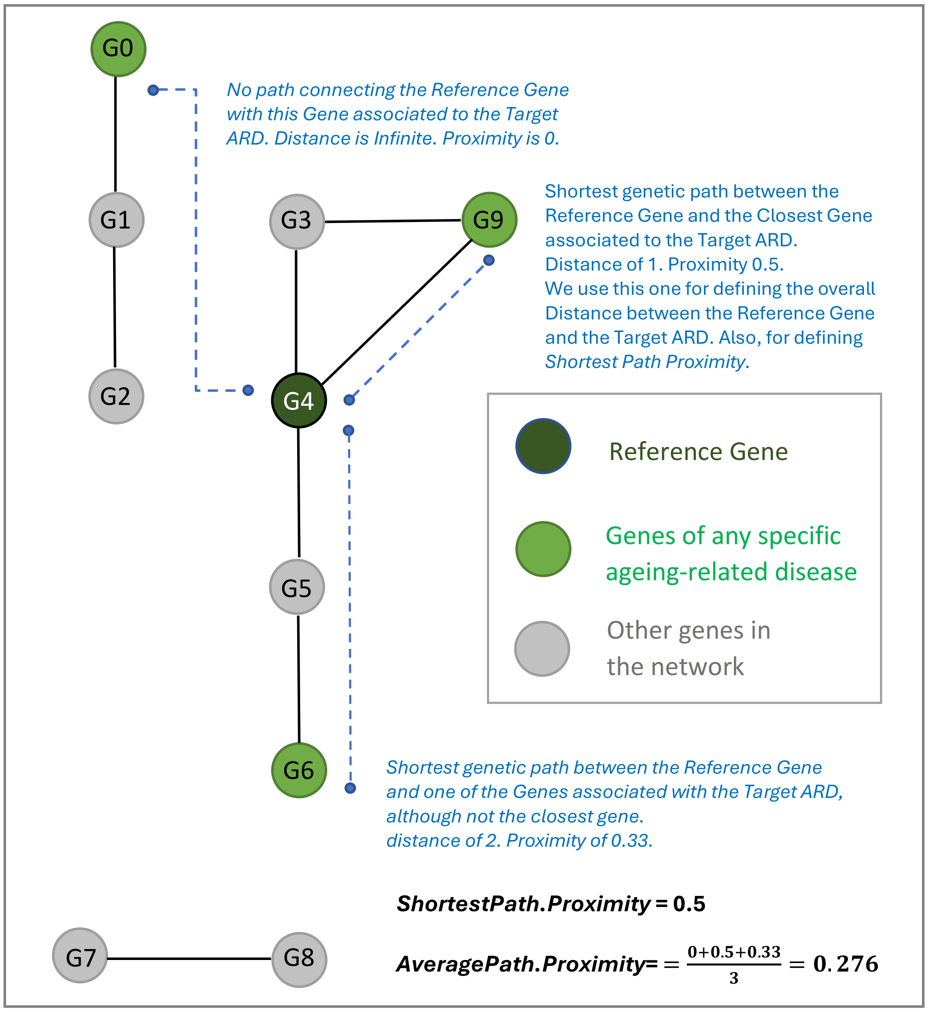

Supplementary Fig. 16: Representation of *Shortest_Path_Proximity* and *Average_Path_Proximity*. A hypothetical $PPI/COX/KEGG$ network with genes G0, G6 and G9 associated with one ARD or ARC is presented. Distance to the ARD is measured from gene G4. The shortest genetic path between the Reference Gene and the Closest gene associated with the Target ARD defines the Distance. As for this hypothetical network, the distance from the Reference Gene G4 to the Target ARD's genes is Infinite for G0, 2 for G6 and 1 for G9. Of these, the shortest distance of 1 between the Reference Gene and G9 represents the Distance between the reference gene and the ARD. This procedure is repeated for each gene in the network and all ARDs. Different ARDs will have different associated genes. The *Shortest_Path_Proximity* is defined based on the shortest Distance between the Reference Gene and the Target ARC or ARD. The *Average_Path_Proximity* is computed based on the mean *Proximity* to all genes associated with the Target ARC or ARD.

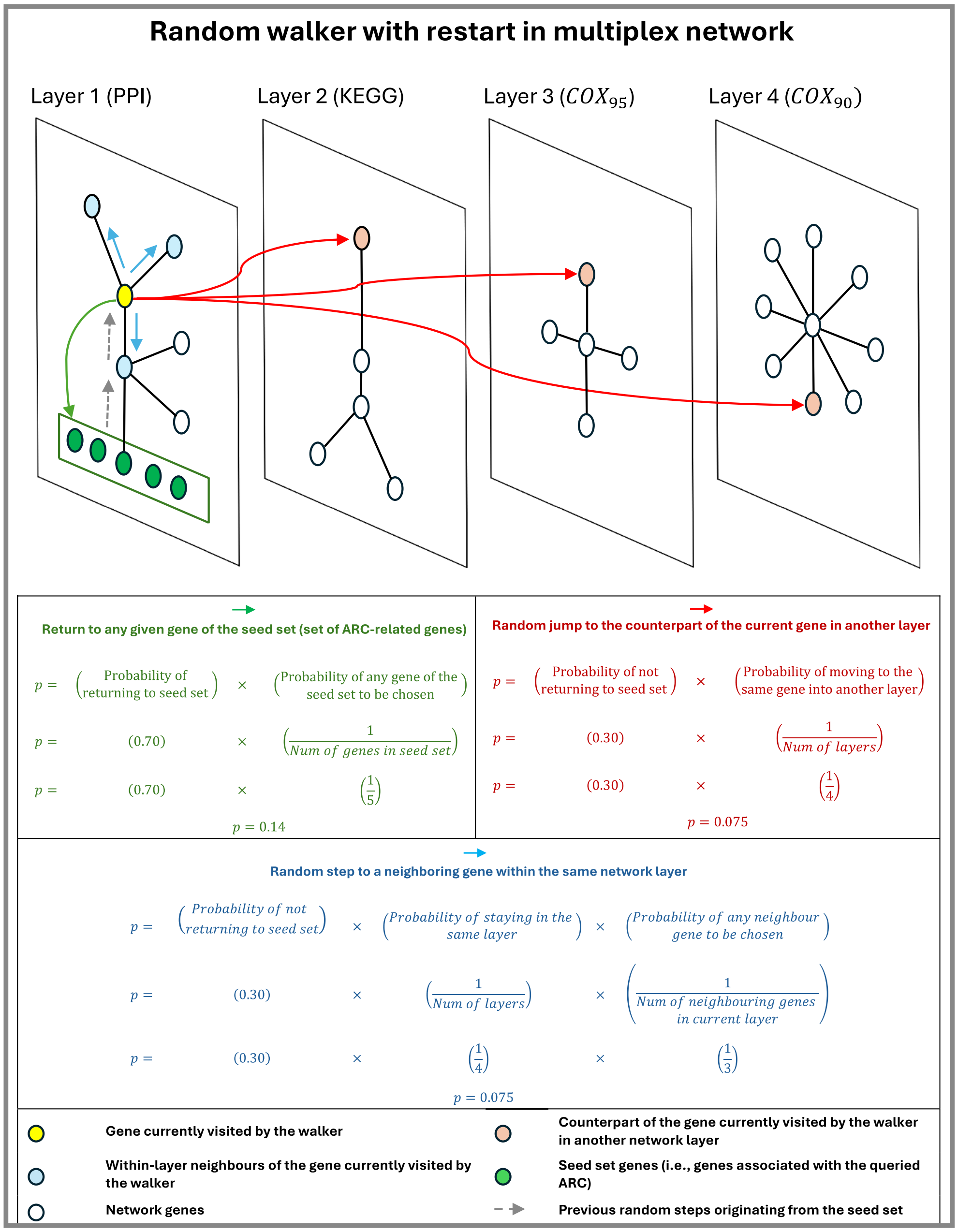

Supplementary Fig. 17: RWR on a *Multiplex* gene network. The schematic illustrates how RWR operates across a *Multiplex* network integrating the four biological layers PPI, $KEGG$, COX_{95} and COX_{90}. Each layer represents a distinct mode of functional gene connectivity, while genes common to multiple layers are vertically linked across layers. Nodes are color-coded to indicate seed genes, the currently visited gene, its within-layer neighbours, and its counterparts across layers. During each iteration, a random walker starting from the seed set, composed of genes associated with a given ARC, has a 70% probability (r=0.7) of returning to any seed gene, and a 30% probability (1–r=0.3) of continuing the exploration. If the walker continues exploring, this 30% probability is evenly distributed across the four network layers (τ=[1, 1, 1, 1]). Within this exploratory phase, the walker can either (i) move to a neighbouring gene within the same layer or (ii) jump to the same gene represented in another layer, if such a counterpart exists. These stochastic choices determine how information flows through both intra-layer and inter-layer connections. The example probabilities shown (0.14 for seed return, 0.025 for intra-layer steps, and 0.075 for inter-layer jumps) illustrate how the walker dynamically balances local exploration—propagating through functionally related genes within a layer—and global diffusion—spreading information across complementary biological layers. After multiple iterations, the system reaches a steady state, where each gene attains a probability value proportional to the fraction of visits received over the total number of steps, representing the long-term likelihood of finding the walker at that gene. This steady-state probability reflects the gene's functional *Proximity* to the seed set, enabling genome-wide ranking of genes associated with ageing and multimorbidity. In the *Monoplex* case, the process is identical except that all random steps are restricted to a single network layer, without inter-layer transitions.

Topological association of genes with ARCs (results)

Permutation tests for Shortest-path distance to ARCs

We next evaluated whether ageing-related genes, disease-associated genes, and high *ARC-Pleiotropy* genes (immunological disorders) exhibit non-random shortest-path distances to ARC-associated genes across our four networks: PPI, COX_{90}, COX_{95}, and $KEGG$. In each case, observed distances were compared against null distributions generated from 10,000 size-matched random samples of all genes present in the respective network (Supplementary Fig. 18 and Table 2 of the main text).

In the PPI network, both $GenAge_{Hum}$*- and* $GenAge_{Mod}$-related genes were separated from ARC-associated genes by significantly fewer edges than expected under random conditions ($GenAge_{Hum}$*=1.72 vs. null=2.16, p_adj=1.6e-3;* $GenAge_{Mod}$*=1.93 vs. null=2.16, p_adj =1.6e-3*). Disease-associated genes were even closer, with a mean of 1.46 edges (*vs. 2.16 null, p_adj =1.6e-3*). The shortest distances overall were observed for high *ARC-Pleiotropy* genes, which were on average separated by only 0.45 edges (*vs. 2.17 null, p_adj =1.6e-3*).

A similar pattern appeared in the $KEGG$ pathways, where human ageing-related genes again showed shorter distances to ARCs than expected ($GenAge_{Hum}$*=1.89 vs. null=2.29, p_adj =1.6e-3), whereas ageing-related genes in model organisms were close to random* $GenAge_{Mod}$ *= 2.21 vs. null=2.28, ns*). Disease-related genes were closer to ARCs than random(*1.44 vs. 2.28 null, p_adj <1.6e-3*), and high *ARC-Pleiotropy* genes remained the most proximal group to ARCs (*≈0.44 vs. 2.29 null, p_adj =1.6e-3*).

The coexpression networks revealed a more nuanced pattern. In the COX_{90} network, $GenAge_{Hum}$ genes were slightly closer to ARCs than expected by chance, but this difference did not remain significant after multiple testing correction (*1.88 vs. 2.26 null, p_adj = 0.16*). In contrast, $GenAge_{Mod}$ genes showed a modest but significant reduction in shortest path distance to ARCs (*1.71 vs. 2.25 null, p_adj = 1.6e−03*). Disease-associated genes were consistently closer to ARCs (*1.38 vs. 2.25 null, p_adj = 1.6e−03*), while high ARC-Pleiotropy genes exhibited the strongest proximity of all groups (*0.52 vs. 2.28 null, p_adj = 1.12e−02*).

In the COX_{95} network, distances to ARCs were generally larger. Neither $GenAge_{Hum}$ nor $GenAge_{Mod}$ genes differed significantly from random expectation (*4.26 vs. 4.72 null, p_adj = 1.0; and 3.89 vs. 4.65 null, p_adj = 0.19*, respectively). Disease-associated genes again showed significantly shorter paths to ARCs (*2.38 vs. 4.64 null, p_adj = 1.6e−03*), whereas high ARC-pleiotropy genes displayed the smallest distances overall (*0.53 vs. 4.86 null*), although this effect did not reach statistical significance after correction (*p_adj = 0.15*).

Permutation tests for RWR-based association with ARCs

We next assessed whether ageing-related genes, disease-associated genes, and high *ARC-Pleiotropy* genes (immunological disorders) exhibit non-random association to ARC-associated genes when measured by RWR. We evaluated this across our four networks: PPI, COX_{90} COX_{95} and $KEGG$ pathways. Observed scores were compared against null distributions derived from 10,000 size-matched random samples of all genes present in the respective network (Supplementary Fig. 19 and Table 3 of the main text).

In the PPI network, ageing-related genes clearly stood out. $GenAge_{Hum}$ had RWR scores nearly four times higher than random expectation (8.*92e-5 vs. null=2.38e-5, p_adj=1.6e-3*),

indicating a strong tendency to remain within ARC-related regions during the random walk. $GenAge_{Mod}$ genes also showed markedly elevated scores (*4.13e-5 vs. 2.39e-5 null, p_adj = 8.0e-3*). In contrast, disease-associated genes showed slightly lower yet not significant RWR connectivity than expected (*1.57e-5 vs. 2.39e-5 null, ns*), and high *ARC-Pleiotropy* genes had the lowest RWR scores overall, but were not significantly different from the null (*1.21e-6 vs. 2.34e-5 null, ns*).

In the $KEGG$ network, ageing-related genes again showed higher-than-expected RWR scores. $GenAge_{Hum}$ genes had mean values of *1.59e-4* compared to *4.56e-5* under the null (*p_adj = 2.4e-2*), and $GenAge_{Mod}$ genes showed a similar increase (*1.13e-4 vs. 4.53e-5 null, p_adj = 1.6e-3*). Disease-related genes, however, were indistinguishable from random (*3.05e-5 vs. 4.54e-5 null, ns*), as were high *ARC-Pleiotropy* genes (*2.93e-5 vs. 4.45e-5 null, ns*).

The coexpression networks showed more heterogeneous outcomes. In the COX_{90}, network, neither $GenAge_{Hum}$ nor $GenAge_{Mod}$ genes differed from random expectation in their RWR scores (*2.15e−5 vs. 2.26e−05 null, p_adj = 1.0; and 2.56e−05 vs. 2.26e−5 null, p_adj = 1.0*, respectively), and disease-associated genes also matched the null (*2.47e−05 vs. 2.27e−5 null, p_adj = 1.0*). In contrast, high *ARC-pleiotropy* genes showed a marked increase in network localization, with RWR scores nearly fivefold higher than expected by chance (*1.23e−04 vs. 2.28e−05 null, p_adj = 6.4e−3*).

A similar pattern was observed in the COX_{95}, network. $GenAge_{Hum}$, $GenAge_{Mod}$, and disease-associated genes again did not differ from random expectation (*p_adj = 1.0* for all comparisons). High *ARC-pleiotropy* genes, however, retained substantially elevated RWR scores relative to the null (*2.93e−4 vs. 4.3e−5 null*), although this effect was only nominally significant after correction (*p_adj = 7.8e−2*).

These results reveal a dual pattern: ageing-related genes (especially $GenAge_{Hum}$) integrate strongly with ARCs in interaction networks (PPI, $KEGG$), while some highly *ARC-Pleiotropic* genes rather integrate in *coexpression networks*, occupying hub-like positions with much higher RWR scores for COX_{90} and COX_{95}.

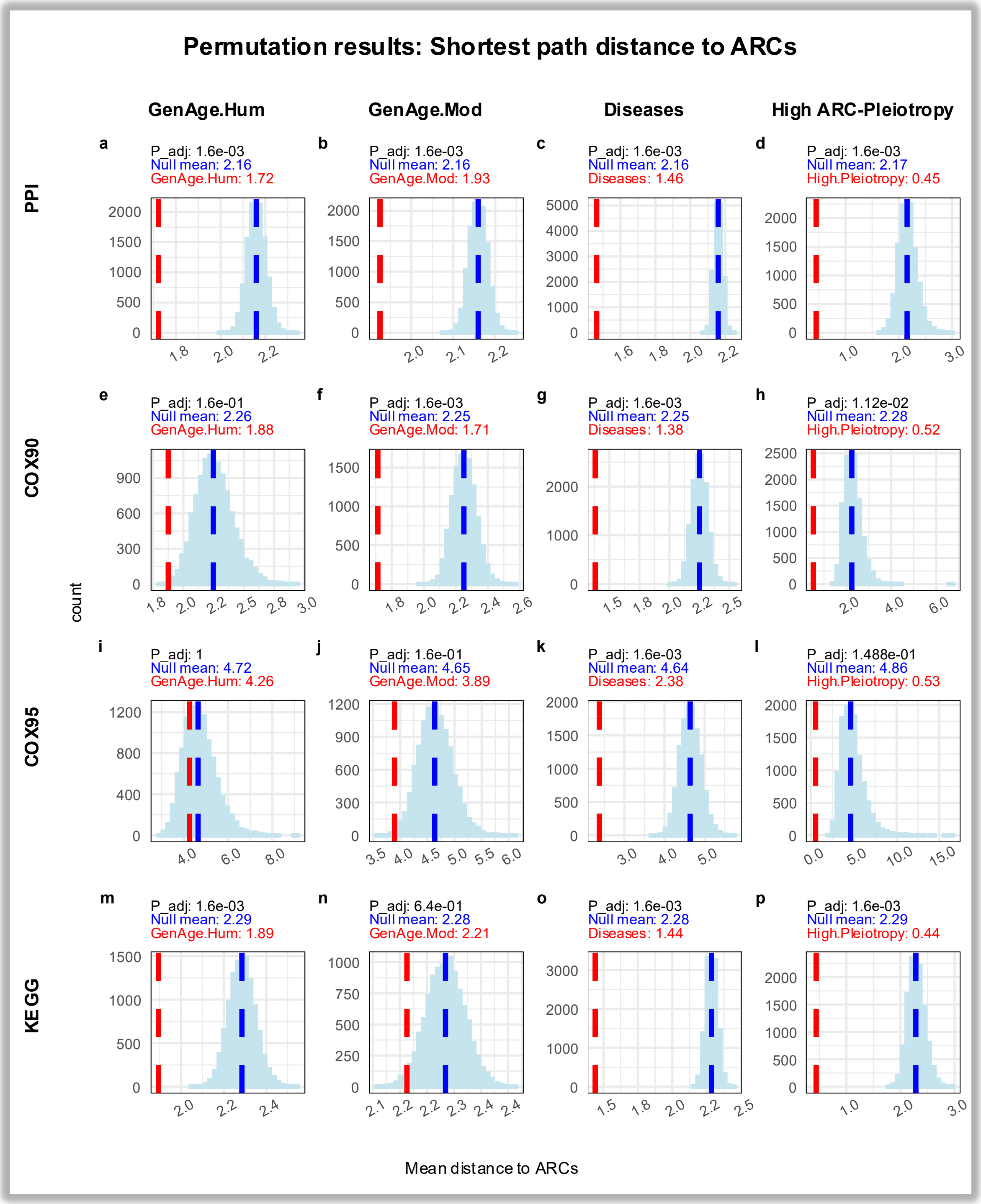

Supplementary Fig. 18. Permutation-based distributions of mean shortest-path distance to ARC-related genes across networks. Permutation-derived null distribution (blue) of the mean shortest-path distance between genes from a given set (columns) and the genes defining each ARC within a specific network (rows). Distances correspond to the minimum number of edges required for a gene to reach the nearest ARC-associated gene within the same network. Blue dashed lines mark the null mean, while red dashed lines indicate the observed mean. Panels are organized by network (rows) and gene set (columns). From top to bottom: PPI, COX_{90}, COX_{95}, and $KEGG$ networks. From left to right: $GenAge_{Hum}$, $GenAge_{Mod}$, Diseases, and *High ARC-Pleiotropy*. Smaller observed distances reflect stronger topological *Shortest_Path_Proximity* to ARC-related genes, suggesting closer functional relationships.

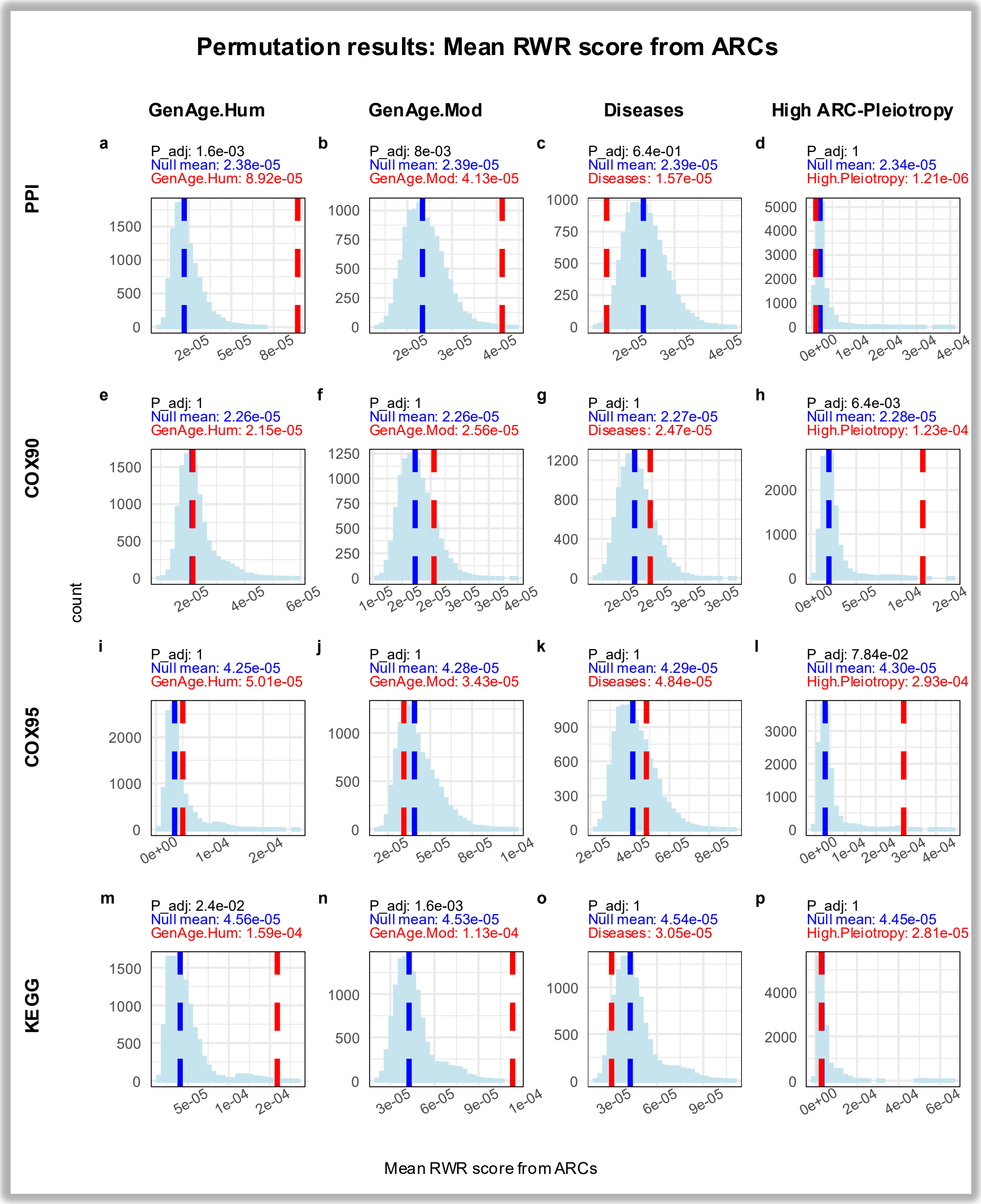

Supplementary Fig. 19. Permutation-derived null distribution (blue) of the mean RWR score between genes from a given set (columns) and the genes defining each ARC within a specific network (rows). The RWR score represents the steady-state probability that a random walker starting from ARC-associated genes will reach members of the tested set, thus capturing their diffusion-based topological association. Blue dashed lines mark the null mean, while red dashed lines indicate the observed mean value. Panels are organized by network (rows: PPI, COX_{90}, COX_{95}, $KEGG$) and gene set (columns: $GenAge_{Hum}$, $GenAge_{Mod}$, Diseases, High *ARC-Pleiotropy*). Elevated observed RWR scores (rightward shifts from the null) denote increased connectivity and functional closeness to ARC-centered subnetworks.

Coexpression Analysis

Supplementary Fig. 20 supports the results depicted in Figs. 3 and 4a of the main text, confirming that *GenAge*-associated genes exhibit higher *intra-set coexpression* than any other group. $GenAge_{Mod}$ exhibited the highest *intra-set coexpression* overall (0.42), followed by $GenAge_{Hum}$ (0.34). Both values were significantly greater than those of every ARC (0.12–0.27) and *ARC-Pleiotropy* group (0.11–0.27) (**** p_adj≤1e−4 in most comparisons). The only exceptions were renal/urology (0.18) and haematology/dermatology (0.24) when compared with $GenAge_{Hum}$, where differences did not reach significance (ns), largely due to their small sample size and higher variance.

Among ARCs, musculoskeletal/trauma (0.27) and cardiovascular (0.25) displayed the highest *intra-set coexpression*, though still significantly lower than both *GenAge* groups (****). Haematology/dermatology (0.24), Endocrine/diabetes (0.21) and neurology/psychiatry (0.21), renal urology (0.18) and gastrointestinal/abdominal disorders (0.18) occupied an intermediate range. By contrast, immunological/systemic disorders showed the lowest mean *intra-set coexpression* (0.12), significantly reduced relative to nearly all other groups (*), except renal/urology where differences were not significant (ns).

When stratified by *ARC-Pleiotropy* level, low *ARC-Pleiotropy* genes (0.25) maintained significantly higher *intra-set coexpression* than high *ARC-Pleiotropy* genes (0.11; ****), mirroring the pattern observed for ageing versus immunological groups. The low *ARC-Pleiotropy* group overlapped more closely with tissue-specific disease modules, whereas high *ARC-Pleiotropy* genes recapitulated the diffuse, weakly coexpressed architecture of immunological/systemic disorders.

Altogether, these results highlight a statistical separation: ageing-related genes consistently form the most tightly coexpressed modules (0.34–0.42), whereas highly *ARC-Pleiotropic* and immunological/systemic disorder genes represent the weakest modules (0.11–0.12), a pattern that was robust to multiple-testing correction.

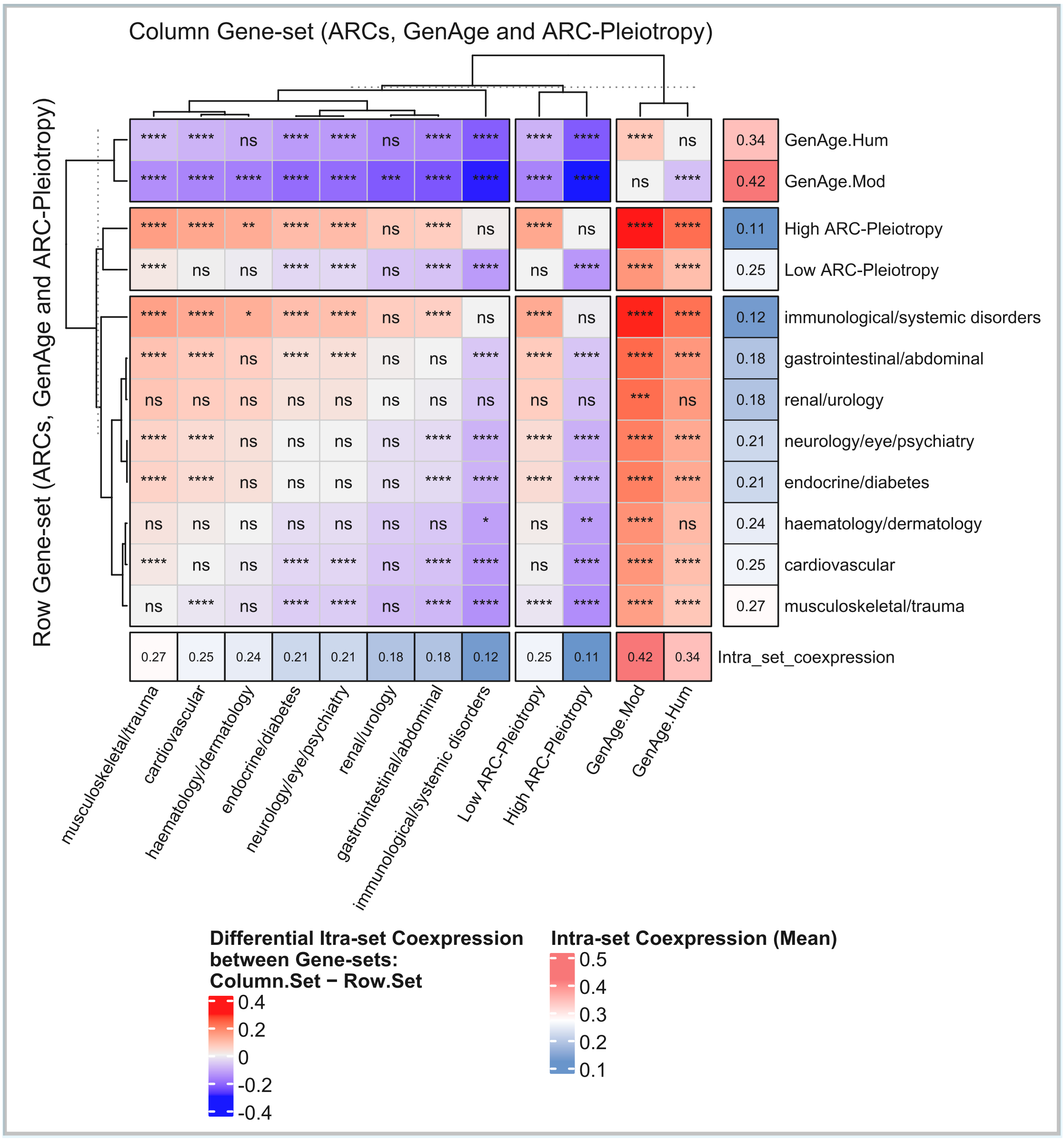

Supplementary Fig. 20: Corrected statistical significance of *Intra-set coexpression* differences. Rows and columns represent groups of *GenAge*-related genes, ARCs-related genes, and genes associated with different degrees of *ARC-Pleiotropy*. The mean *intra-set coexpression* of groups at columns is depicted in the bottom annotation. The mean *intra-set coexpression* of groups in rows is depicted in the right-most annotation. For a group at a column and a group at a row, the difference in means is calculated by subtracting the mean *intra-set coexpression* of the column group (in bottom annotation) minus the mean specificity at the row group (right-most annotation). This subtraction results in the heatmap colours: red is positive differences (the column value exceeds the row), blue denotes negative differences, and white indicates no difference. Text within each cell represents the statistical significance of the mean differences (Asterisks denote the statistical significance of this difference (*ns p_adj>5e-2, * p_adj≤5e-2, ** p_adj≤1e-2, *** p_adj≤1e-3, **** p_adj≤1e-4*). Significances were computed using two-sided t-tests and corrected with Bonferroni.

Tissue Specificity and tissue expression

Supplementary Fig. 21 compares the mean tissue-specificity of each gene-set, highlighting contrasting profiles between ageing, ARC, and *ARC-Pleiotropy* groups. Both *GenAge* groups displayed the lowest tissue specificity (0.36–0.38), significantly below most ARC categories (**** $p\_adj \leq 1e-4$ in most comparisons), consistent with their broad, systemic expression across tissues.

In contrast, *ARC-Pleiotropy* groups showed elevated specificity values, with high *ARC-Pleiotropy* genes (0.71) representing the most tissue-specific subset, significantly higher than Low *ARC-Pleiotropy* (0.57) and all ARC groups (0.56–0.61) (****) other than immunological/systemic disorders (0.72). The low *ARC-Pleiotropy* group exhibited specificity values (0.57) that align with the lowest-specific ARCs such as cardiovascular (0.56), musculoskeletal/trauma (0.57), and haematology/dermatology (0.57).

Among ARCs, the most tissue-specific sets were immunological/systemic disorders (0.72) and gastrointestinal/abdominal (0.63), both displaying significantly higher *Tau* values than both *GenAge* groups and low *ARC-Pleiotropy* groups (****). Conversely, renal/urology (0.62), endocrine/diabetes (0.57), and neurology/eye/psychiatry (0.61) exhibited intermediate values, while haematology/dermatology (0.57), musculoskeletal/trauma (0.57) and cardiovascular (0.56) displayed the broadest expression patterns among diseases.

Altogether, these findings reveal a divergence between *GenAge*-associated and *ARC-Pleiotropic* genes: ageing-related genes are broadly expressed across tissues, consistent with systemic roles in organismal regulation, whereas high *ARC-Pleiotropy* (i.e. immunological systemic disorder) genes are highly tissue-specific, reflecting context-dependent and immune-related regulation.

Tau Index Distribution Across *ARC-interactors* and *ARC-Pleiotropic* Genes

Supplementary Fig. 22 presents box plots of the distribution of *Tau* Scores across diverse ranges of *ARC-Interactions* and *ARC-Pleiotropies*.

In the PPI network (Supplementary Fig. 22a), *Tau* values decreased progressively with higher interactivity, from a median of 0.28 in *ARC-Interaction_1* to 0.17 in *ARC-Interaction_4+*, with significant contrasts across nearly all categories, including a modest but significant difference between interactions 1 and 4+. Median *Tau* values also tended to lie near the center of their interquartile ranges. Gene counts declined sharply with increasing interactivity (2,452 → 496). COX_{90} showed a similar decline in *Tau* (Supplementary Fig. 22c), from 0.37 in group 1 to 0.25 in group 3 and ~0.24 in group 4+. In this case, however, gene counts behaved differently, rising substantially at 4+ from <800 in groups 1–3 to 4,034 genes. Thus, both networks follow a trend of declining *Tau*, but diverge markedly in gene representation at higher interactivity.

COX_{95} and *ARC-Pleiotropy* both displayed non-linear profiles, with stable *Tau* at lower interaction levels and sharp increases at the highest category (Supplementary Fig. 22b, e). In COX_{95}, *Tau* values hovered around 0.23–0.22 in groups 1–3 but rose sharply to 0.85 in group 4+, accompanied by a modest increase in gene counts (513 → 625). Most pairwise comparisons were significant, except among ARC-Interactivity groups 1–2, 2–3, and 1–3.

$KEGG$ exhibited a more stable pattern overall (Supplementary Fig. 22d). *Tau* values remained broadly similar across groups 1–3 (~0.41–0.49), with only a modest decline in group 4+ (0.36). Gene counts decreased progressively (1,330 → 304). Pairwise comparisons were mostly non-significant, with only marginal contrasts involving the highest category. This

relative uniformity distinguishes $KEGG$ from the other networks, as *Tau* distributions remained largely consistent across interactivity levels.

Tissue expression across tissues

We next analyzed tissue expression patterns across 30 human tissues to determine whether ageing-related genes, disease-associated genes, and high *ARC-Pleiotropy* immunological disorder genes occupy distinct transcriptional profiles. Expression distributions for these three categories are compared in Supplementary Figs. 23-24, while their gene-tissue expression heatmap and corresponding *Tau* distributions are shown in Supplementary Fig. 25.

Across virtually all tissues, *GenAge* genes ($GenAge_{Hum}$ and $GenAge_{Mod}$) displayed higher and more consistent expression than the other categories. This broadly distributed transcription is consistent with their low *Tau* values, reinforcing that ageing-related genes act across multiple physiological contexts rather than being confined to a single organ system. Their expression does not peak sharply in any specific tissue, but instead remains relatively uniform, suggesting a role in basal cellular maintenance and systemic ageing processes.

Disease-associated genes showed intermediate expression levels with tissue-dependent variation, accompanied by a broad *Tau* distribution, consistent with a mixed biological scope that combines shared and more specialized functions. Within this category, low *ARC-Pleiotropy* genes exhibited heterogeneous but lower overall expression, with localized peaks most frequently observed in reproductive (testis, ovary), digestive (liver, intestine), and immune tissues (spleen, lymphoid organs), reflecting moderate specialization rather than global transcriptional activity.

In contrast, high *ARC-Pleiotropy* (immunological disorder) genes exhibited the lowest global expression across most tissues, yet simultaneously displayed the highest tissue-specificity, with *Tau* values clustering near 0.8–1.0 (Supplementary Fig. 25). Expression was stronger in testis, blood, and immune-related tissues (e.g., spleen, lymphoid), suggesting that these genes are primarily active in immune or reproductive contexts. This suggests that the broad phenotypic relevance does not arise from ubiquitous expression, but instead from context-dependent activity in a small number of regulatory or stress-responsive tissues.

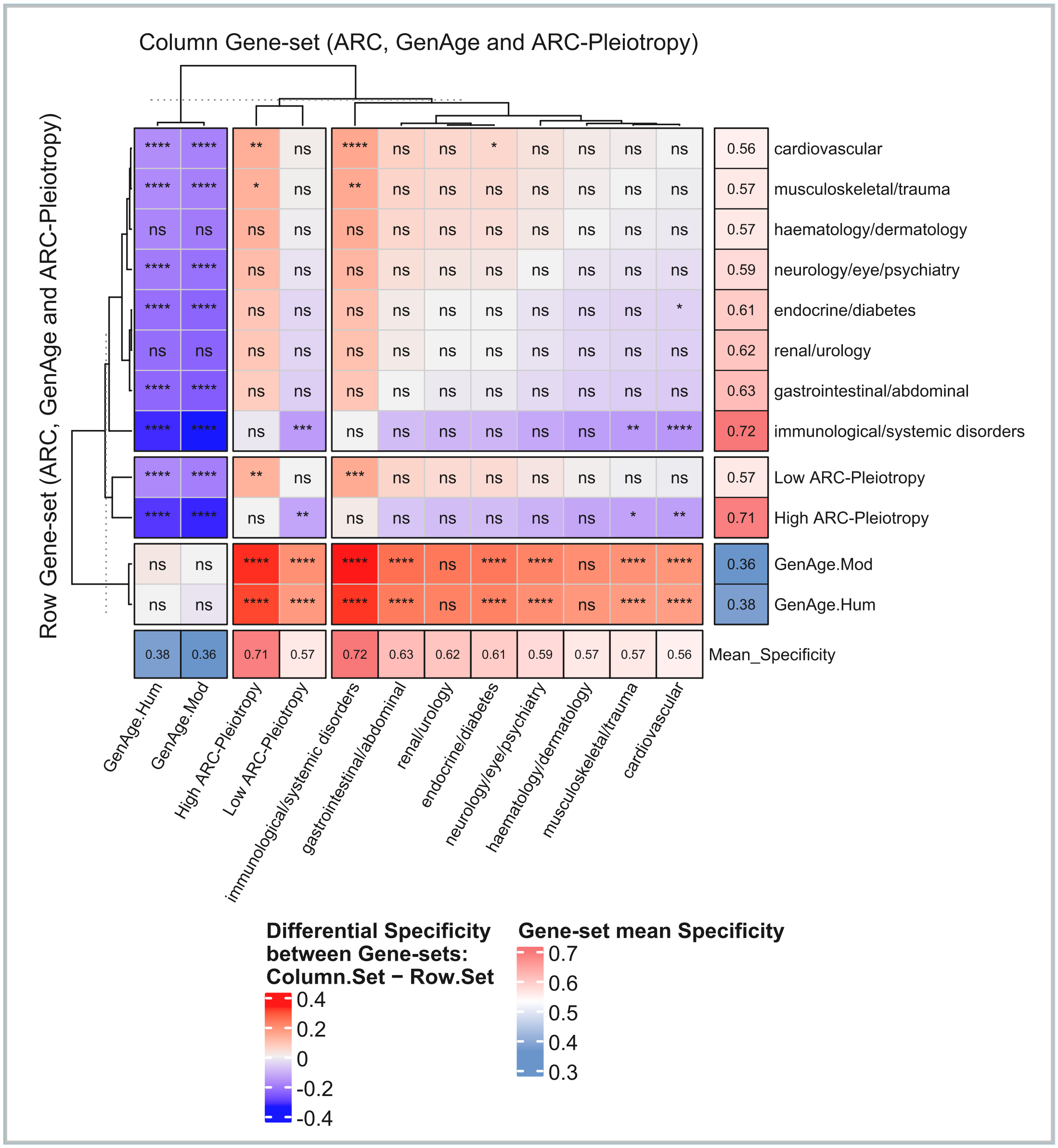

Supplementary Fig. 21: Corrected statistical significance of tissue specificity differences. Rows and columns represent groups of GenAge-related genes, ARCs-related genes, and genes associated with different degrees of ARC-Pleiotropy. The mean specificity of groups at columns is depicted in the bottom annotation. The mean specificity of groups in rows is depicted in the right-most annotation. For a group at a column and a group at a row, the difference in means is calculated by subtracting the mean specificity of the column group (in button annotation) minus the mean specificity at the row group (right-most annotation). This subtraction results in the heatmap colours: red is positive differences (where the column value exceeds the row), blue denotes negative differences, and white indicates no difference. Text within each cell represents the statistical significance of the mean differences (Asterisks denote the statistical significance of this difference (*ns p_adj>5e-2, * p_adj ≤5e-2, ** p_adj ≤1e-2, *** p_adj ≤1e-3, **** p_adj ≤1e-4*). Significances were computed using two-sided Wilcoxon rank-sum tests due to the non-Gaussian distribution of the data and corrected with Bonferroni. The differences were primarily non-significant for most groups. Broadly, the GenAge groups tend to have significantly lower specificity than most other groups.

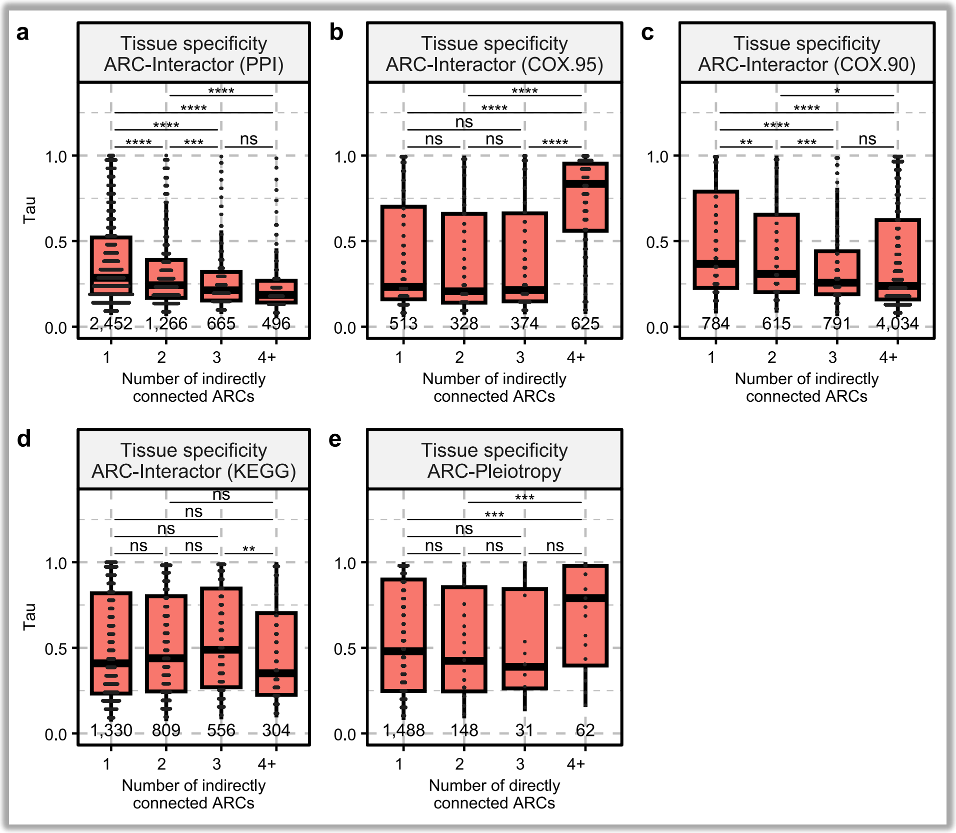

Supplementary Fig. 22: *Tau* score vs and *ARC interactions* and *ARC-Pleiotropy*. The X-axis represents the number of ARCs a gene can reach, either indirectly (*ARC-Interation* corresponding to Sub-Figs a-f) or directly (*ARC-Pleiotropy,* corresponding to Sub-Fig e). The Y-axis denotes the corresponding *Tau* value, which reflects tissue specificity. Specifically, the bar presented at the X-value "1" in each graph depicts the distribution of *Tau* values for the set of genes that are associated with only one ARC. The number "2" similarly represents genes associated with two ARCs, "3" for those associated with three ARCs, and the "4+" symbolizes the *Tau* values for genes that are directly or indirectly associated with four or more ARCs. **a**. PPI network. **b.** COX_{95} network. **c.** COX_{90} network. **d**. $KEGG$ network. **e**. *Tau* vs *ARC-Pleiotropy* across all the ARDs genes. Statistical differences were computed using two-sided Wilcoxon rank-sum test and adjusted for multiple tests with Bonferroni.

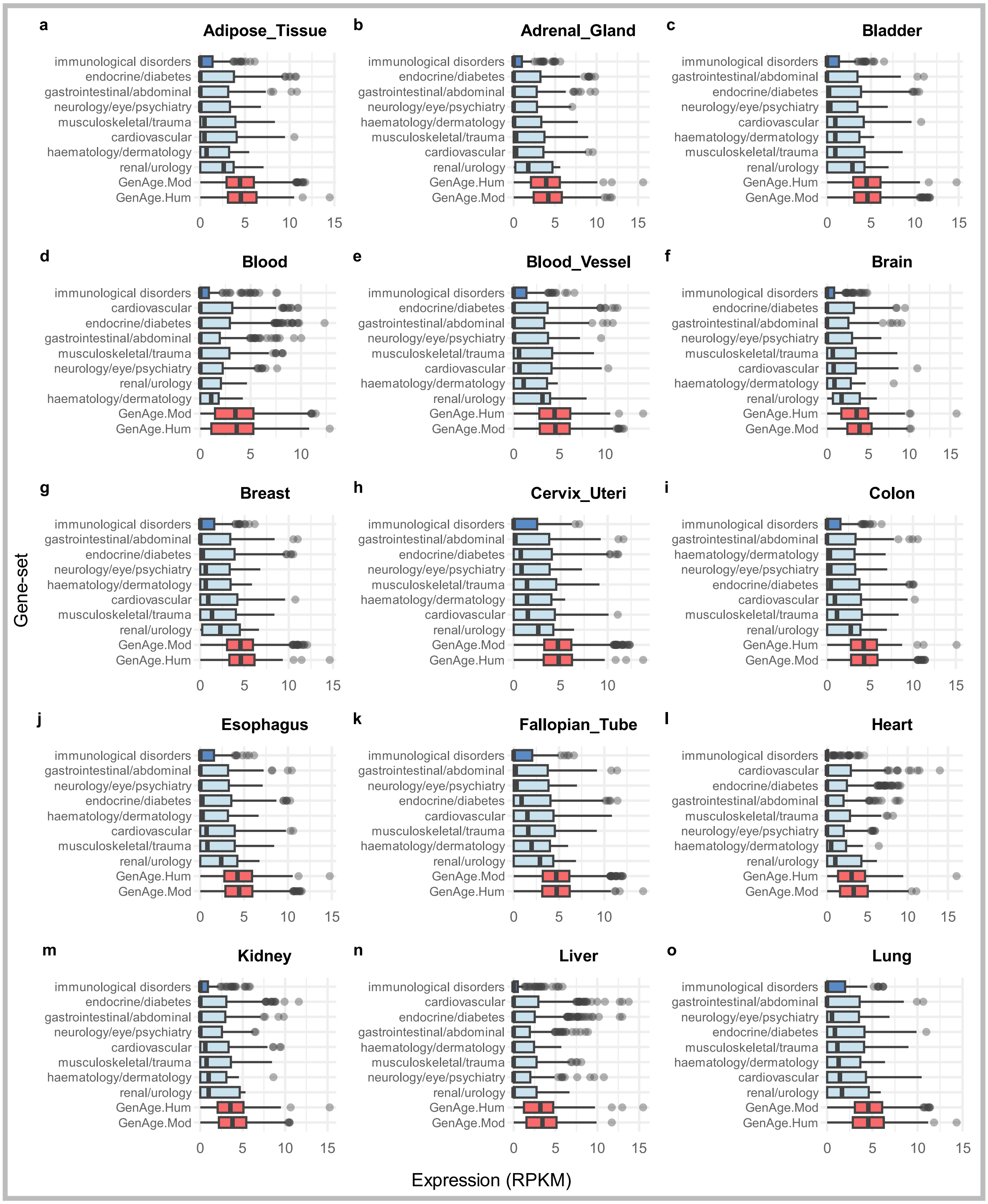

Supplementary Fig. 23. Distribution of tissue expression of disease-associated genes across ARCs. Panels show horizontal boxplots summarizing the distribution of expression levels of genes associated with different disease categories (*e.g.*, immunological, cardiovascular, endocrine, neurological, etc.) across human tissues. Each box represents the dispersion and median expression of genes from a given category, while the horizontal axis indicates the number of expressed genes per tissu e. Data were obtained from *GTEx*, and expression values were normalized per tissue to reflect relative transcriptional activity within each ARC. Blue tones correspond to lower expression medians, whereas red tones denote higher cross-tissue expression, allowing visual comparison of tissue breadth and activity across disease domains. Panels are organized by network layer (PPI, $KEGG$, COX_{95}, and COX_{90}), and display two reference groups, $GenAge_{Hum}$ and $GenAge_{Mod}$, to facilitate comparison between disease- and ageing-associated expression patterns. This figure corresponds to the first part of the tissue expression analysis. The second part (Supplementary Fig. 24) extends this comparison to ageing-related genes and their coexpression with disease-associated modules across the same tissue categories.

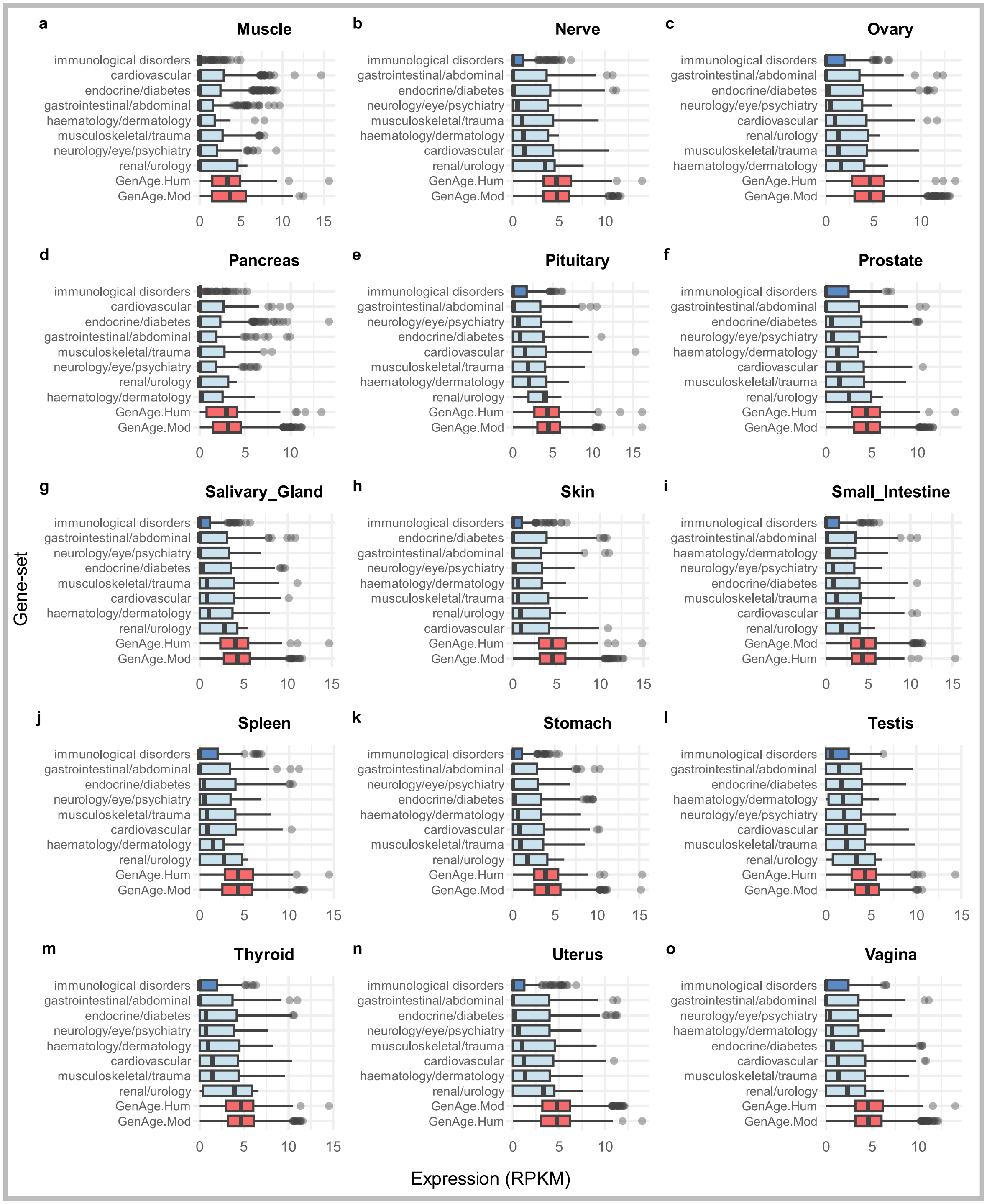

Supplementary Fig. 24. Comparative tissue expression of ageing-related genes relative to disease-associated categories across ARCs. This second panel complements Fig. 23 (part 1) by showing horizontal boxplots of tissue expression for ageing-related genes ($GenAge_{Hum}$ and $GenAge_{Mod}$) compared with disease-associated categories across the same tissue groups. Each boxplot summarizes the distribution of expression levels (*GTEx* TPM > 1) of genes linked to specific ARCs, highlighting differences in expression breadth and median activity between ageing- and disease-related gene sets. Color tones correspond to relative expression levels, from blue (lower median expression) to red (higher median expression), enabling visual assessment of which tissues or disease systems display broader transcriptional engagement. Panels are arranged by network layer (PPI, $KEGG$, COX_{95}, COX_{90}), mirroring the structure of Supplementary Fig. 23 (part 1).

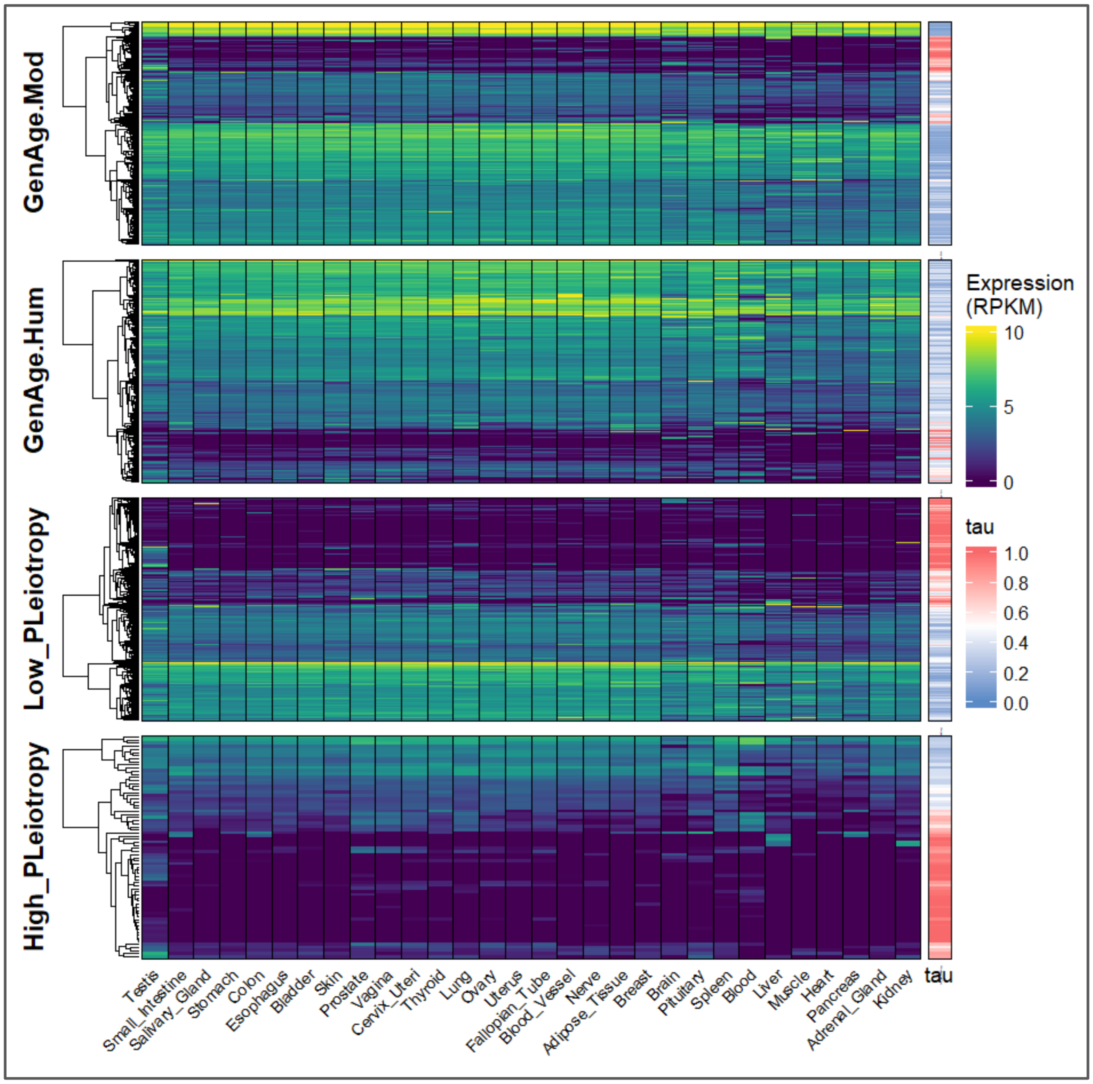

Supplementary Fig. 25. Expression and tissue-specificity of ageing- and *ARC-Pleiotropy*-related genes. Expression heatmaps across *GTEx* tissues show distinct transcriptional breadth among $GenAge_{Mod}$, $GenAge_{Hum}$, *Low ARC-Pleiotropy*, and *High ARC-Pleiotrop*y genes. Expression levels (RPKM) are shown in green-to-yellow, while the vertical color bar on the right represents each gene's tissue-specificity index (*Tau*), from 0 (broad expression) to 1 (highly tissue-specific). Ageing-related genes exhibit widespread expression, whereas *ARC-Pleiotropic* genes show progressively higher τ values and more restricted activity, with strongest enrichment in testis, brain, and adrenal gland.

Machine Learning

This section complements the *Methods* section of the manuscript by providing a detailed description of how ML was used to predict novel genes associated with ageing. The 307 genes curated in $GenAge_{Hum}$, hereafter referred to as *ageing-related genes*, were used as the positive class, while all genes present in the networks were treated as instances in the classification task. Predictive features were derived from network-based topological properties that quantify the relative positioning of each gene with respect to genes associated with ARCs and *ARDs* within each network layer. These connectivity features capture complementary aspects of gene–disease architecture, including local neighbourhood structure, path-based proximity, and diffusion-based relationships, enabling a systematic assessment of how network organization informs ageing-gene prediction.

Definition of Connectivity Measures

To quantify how ageing-related genes are positioned relative to ARC- and ARD-associated genes in network space, we defined four complementary measures of connectivity (Supplementary Figs. 16–17, 26):

- ***Shortest_path_Proximity_to_disease***. Inverse of the shortest path length plus one between a reference gene and any disease-associated gene. Values decrease with increasing distance (*e.g.*, one-step=0.5, two-step=0.33). Because *Proximity* is the inverse of distance, higher values correspond to shorter paths. This formulation avoids infinite values for genes with no path to disease-associated targets. For each gene, the

score corresponds to the maximum *Proximity* (*i.e.*, the shortest path) among all disease-associated targets.

- ***Average_path_Proximity_to_disease***. Mean of *Proximity* values (*1 / [Distance + 1]*) between a reference gene and all disease-associated genes. Higher values indicate shorter average distances. The average is computed only across connected targets to prevent bias from disconnected nodes.
- ***Neighbours_associated_with_disease***. Number of direct neighbours of a reference gene that are annotated as disease-associated. Unlike *Proximity*-based metrics, this measure does not depend on path length and captures only immediate adjacency within the network.
- ***RWR_seeded_from_diseases***. Steady-state probabilities of visiting each gene during a RWR process initiated from disease seed nodes. At each step, the walker either moves to a neighbouring node with uniform transition probability or returns to the seed set with a predefined restart probability (*r*). The resulting steady-state vector represents the probability distribution of reaching each gene from disease-associated nodes after infinite iterations of the diffusion process.

Dataset Construction

Using the four connectivity measures (*Shortest_Path_Proximit_to_diseasey*, *Average_Path_Proximity_to_disease*, *Neighbours_associated_with_disease*, and *RWR_seeded_from_diseases*) connectivity profiles were organized into a structured collection of datasets capturing complementary aspects of gene–disease architecture across multiple

network layers. Each dataset encodes the relative positioning of individual genes with respect to ARCs and ARDs within one or more network (Supplementary Table 5).

Connectivity scores were computed across the PPI, COX_{90}, COX_{95}, and $KEGG$ networks and summarized at two levels of aggregation: ARC-based (8 features per gene) and ARD-based (57 features per gene). These results were then organized into parallel datasets according to three complementary design strategies. First, a $Metric \times Network$ design combined every connectivity measure with every network, generating a complete set of ARC- and ARD-based datasets. Second, *per-network* and *per-metric* integrations aggregated scores across metrics or networks, respectively, to yield composite connectivity profiles. Finally, a *Multiplex RWR seeded_from_diseases* analysis was performed on an integrated multilayer network that combined the PPI, coexpression, and $KEGG$ layers, producing additional ARC-, ARD-, and combined-level datasets.

Together, this framework produced 43 datasets systematically describing gene connectivity patterns at multiple resolutions, enabling direct comparison of local, path-based, and diffusion-based relationships across networks and disease groupings. Further details of them are explained in this section.

Levels of aggregation

The *Shortest_Path_Proximity_to_diseases*, *Average_Path_Proximity_to_diseases*, *Neighbours_associated_with_diseases*, and *RWR_seeded_from_diseases* metrics were computed at two levels of aggregation:

- ARC-based: one score per gene for each of the eight ARCs (8 features).
- ARD-based: one score per gene for each of the 57 ARDs (57 features).

$\boldsymbol{Metric} \times \boldsymbol{Network}$ design

For each of the four networks (PPI, COX_{90}, COX_{95}, $KEGG$), all four measures were calculated at both ARC- and ARD-based levels. This design yielded:

- 16 ARC-based datasets (4 networks × 4 measures).
- 16 ARD-based datasets (4 networks × 4 measures).
- In total, 32 datasets were generated in this category.

***Per-network* integration**

Within each network, the four measures were integrated into a single feature set, with both ARC-based (8 features) and ARD-based (57 features) scores included. Thus, each gene was represented by 65 features in these datasets. One integrated dataset was created per network, producing 4 datasets in total.

***Per-metric* integration**

Conversely, for each measure, we combined results across all networks, retaining only genes shared among them. Again, both ARC- and ARD-based scores were included (65 features per gene). One dataset was generated per measure, producing 8 datasets in total.

Multiplex RWR_seeded_from_diseases

Lastly, a *Multiplex* network was constructed by integrating PPI, COX_{90}, COX_{95} and $KEGG$ into a unified multilayer graph. Each layer preserved its native topology, while all layers shared an identical set of gene nodes connected through inter-layer links joining each gene to its counterparts across layers. During the diffusion process, the random walker had a 30% probability of not returning to the seed set. Within this probability, 0.25 was assigned to remaining in the current layer and 0.25 divided equally across the other layers, allowing

transitions between equivalent gene nodes across network types. This configuration enabled information originating in one connectivity layer to propagate through others while preserving the identity of each gene. RWR was then applied on this structure to estimate steady-state association probabilities from disease seed genes. Scores were computed at three aggregation levels: ARC-based (8 features), ARD-based (57 features), and combined ARC+ARD (65 features), resulting in three datasets in total.

Summary of datasets

Altogether, the strategy produced 47 datasets:

- $Metric \times Network$: 32
- *Per-network* integration: 4
- *Per-metric* integration: 8
- *Multiplex RWR_seeded_from_diseases*: 3

Machine Learning Strategy

Algorithm choice

Because our datasets contained between 6,000 and 11,000 genes per network, but only ~300 annotated as ageing-related, the classification task was highly imbalanced. To address this, we implemented Balanced Random Forests (BRF), an ensemble method specifically designed for imbalanced data. BRF resamples the minority class at each tree construction, thereby reducing bias toward the majority class and improving sensitivity for ageing-gene prediction (Supplementary Methods).

Model training

Each BRF model was built with 500 decision trees, implemented in the *imbalanced-learn Python* library (Lemaître et al., 2017). At each node, candidate features were sampled randomly from either *n* or *log2(n)*, where *n* denotes the number of features. Both class-balanced and undersampling-balanced weight configurations were tested. Hyperparameters were optimized using nested cross-validation (CV), with 10 outer folds and 5 inner folds, implemented via *GridSearchCV* from *scikit-learn*. The *random_state* was fixed at 42 to ensure reproducibility.

Evaluation metric

Predictive performance was assessed using the Area Under the ROC Curve (AUC) as the primary metric (Supplementary Table 6). AUC was selected for its robustness to class imbalance and its ability to capture performance across all probability thresholds, rather than at a single decision cutoff.

Predicted Genes and Functional Enrichment

Models Performance

The performance of all the combinations of datasets and algorithms is depicted in Supplementary Table 6. The *Multiplex RWR_seeded_from_ARDs* configuration achieved the highest predictive performance of all tested models (AUC=0.88), confirming that diffusion-based propagation across multiple molecular layers captures the most informative topological signals for ageing-gene prediction.

This result illustrates that integrating the four networks (PPI, COX_{90}, COX_{95} and $KEGG$) enhances robustness and generalization beyond any single network.

Across connectivity metrics (row-wise means), diffusion-based and path-averaging approaches consistently outperformed local descriptors. *RWR_seeded_from_ARDs* obtained the strongest mean AUCs 0.74y), followed by *Shortest_path_Proximity_to_ARDs* (≈0.72) *and Average_path_Proximity_to_ARDs* (≈0.72). These trends indicate that long-range connectivity and distributed information flow provide more predictive power than immediate neighbourhood overlap alone. ARD-based datasets also surpassed their ARC-based counterparts by roughly 0.02–0.03 AUC points on average, suggesting that disease-level resolution offers finer discrimination of ageing-related network architecture.

Across network layers (column-wise means), predictive accuracy increased from *coexpression networks* (COX_{90}=0.57; COX_{95}=0.57) to PPI (0.81) and $KEGG$ (0.81), reaching its maximum in the *Multiplex* configuration (0.86) and remaining high in the Combined ensemble (0.73).

Candidate genes

From the *Multiplex RWR_seeded_from_ARDs* predictions, we prioritized genes with high ageing-related probabilities (≥ 0.9). The top ten candidates (Supplementary Table 7) represent high-confidence false positives, genes not annotated in *GenAge* yet consistently predicted as ageing-associated.

Notably, these candidates show strong multilayer connectivity to ARD modules, particularly through the PPI and $KEGG$ layers, where most display multiple ARC-level interactions (2–5 per layer). Top-ranked predictions include *SMAD2*, *SMAD4*, *CSNK2A1*, *HSP90AB1*, and *MAPK1* (genes deeply embedded in intracellular signaling and regulatory cascades). Interestingly,

none of these candidates overlapped with previously annotated *GenAge* entries, suggesting that the *Multiplex* diffusion approach identifies novel regulatory hubs potentially missed by single-layer analyses.

Functional enrichment

Functional enrichment analysis of the top 30 genes predicted by the *Multiplex RWR_seeded_from_ARD* model revealed a strong overrepresentation of signaling-centric regulatory processes (Supplementary Table 8). Significantly enriched GO Biological Process terms (FDR < 0.05) converged on intracellular and intercellular signaling regulation, including intracellular signal transduction (GO:0035556), regulation of cell communication (GO:0010646), and regulation of signal transduction (GO:0009966). These processes were closely linked to signal propagation mechanisms, notably protein phosphorylation (GO:0006468). In addition, the gene set was enriched for regulatory control of cellular fate and physiology, encompassing regulation of developmental processes (GO:0050793), positive regulation of metabolic processes (GO:0009893), responses to endogenous stimuli (GO:0009719), and programmed cell death (GO:0012501). Overall, these enrichments indicate that high-confidence predictions preferentially map to pathways governing signal integration, metabolic regulation, and cell fate decisions, which are central to ageing-related physiology.

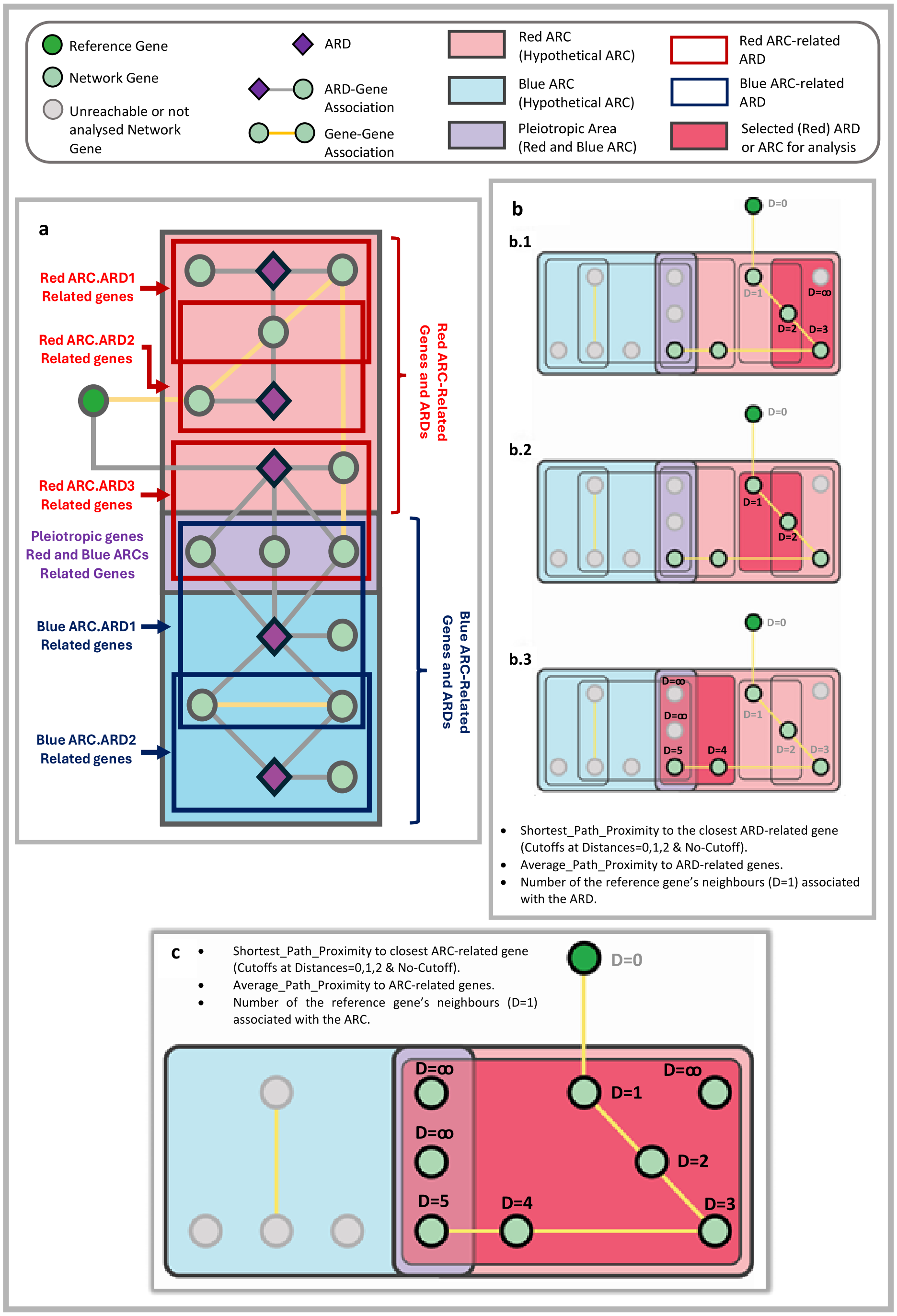

Supplementary Fig. 26: *Proximity*-based ML-features. Distances between the Reference Gene (dark green circle), ARDs (purple diamonds), and ARCs (red and blue sections). Green circles are genes. Yellow lines represent Gene-Gene interactions and grey lines represent Gene-ARD associations. Genes associated with each ARD are contained inside a rectangular for ease of identification. **a**. Two hypothetical ARCs (Red and Blue) and the intersections between them (light purple section). The intersection occurs for ARD-related genes in common between the two ARCs, but ARDs (purple diamonds) do not overlap. **b**. Distance between the Reference Gene and the genes associated with the ARD of interest, highlighted by a red rectangle. The purple diamonds (representation of ARDs) are omitted to visualize only the ARD-related genes (the calculation is based solely on the gene interaction network). The ARD-related feature values are assigned to the reference gene as explained in Supplementary Table 5 for the algorithms *Shortest_Path_Proximity_to_ARD*, *Average_Path_Proximity_to_ARD* and *Neighbours_associated_with_ARD*. **c**. ARC-related feature value assigned to the reference gene is similar to that in Subfigure 'b' but using genes associated with ARCs (*i.e.*, groups of ARDs) instead of single ARDs. The ARC-related feature values are assigned to the reference gene as explained in Supplementary Table 5 for the algorithms *Shortest_Path_Proximity_to_ARC*, *Average_Path_Proximity_to_ARC*, and *Neighbours_associated_with_ARC*.

Supplementary Table 5: Gene-ARD/ARC association algorithms (see Supplementary Figs. 16, 17 and 26).

Algorithm	Description	Target
Shortest_Path_Proximity_to_ARD	Inverse shortest-path distance from queried genes to ARD-associated genes.	ARD
Average_Path_Proximity_to_ARD	Inverse mean shortest-path distance from queried genes to all ARD-associated genes.	
Neighbours_associated_with_ARD	Number of neighbouring genes associated with the queried ARD.	
RWR_seeded_from_ARD	Steady-state probabilities of reaching queried genes from random walks initiated at ARD-associated genes.	
Shortest_Path_Proximity_to_ARC	Inverse shortest-path distance from queried genes to ARC-associated genes.	ARC
Average_Path_Proximity_to_ARC	Inverse mean shortest-path distance from queried genes to all ARC-associated genes.	
Neighbours_associated_with_ARC	Number of neighbouring genes associated with the queried ARC.	
RWR_seeded_from_ARC	Steady-state probabilities of reaching queried genes from random walks initiated at ARC-associated genes.	

Supplementary Table 6. Predictive performance (in AUC) of multiple network-based algorithms evaluated across our four single-layer networks (PPI, COX_{90}, COX_{95}, $KEGG$) and two integrative configurations (*Multiplex* and *Combined*). Algorithms include four topological Proximity measures (Shortest Path Proximity, Average Path Proximity, *Neighbours associated with Diseases*, and *RWR seeded from diseases*), each computed with respect to either ARD or ARC. A composite "Combined" model aggregates predictions from all methods. The "Mean" column on the right represents the average performance of each algorithm across all network configurations, reflecting its overall robustness and generalization capacity. The "Mean" row at the bottom summarizes the average performance of each network across all algorithms, providing an estimate of how predictive each molecular layer is on average. Color intensity ranges from blue (lowest) to red (highest), with white representing intermediate values, providing a visual gradient of relative performance.

Dataset/Algorithm	PPI	COX_{90}	COX_{95}	$KEGG$	*Multiplex*	Combined (per-metric)	Mean
Shortest_Path_Proximity_to_ARD	81	55	56	81		86	72
Shortest_Path_Proximity_to_ARC	74	56	47	73		83	67
Average_Path_Proximity_to_ARD	80	55	55	81		87	72
Average_Path_Proximity_to_ARC	78	56	53	80		84	70
Neighbours_associated_with_ARD	75	48	44	71		84	64
Neighbours_associated_with_ARC	73	51	50	70		84	66
RWR_seeded_from_ARD	82	59	50	81	88	81	74
RWR_seeded_from_ARC	79	60	44	80	83	79	71
Combined (per-network)	81	57	57	81	88		73
**Mean**	78	55	52	78	86	84	

Supplementary Table 7. Top-ranked gene predictions from the best-performing model (*Multiplex RWR_seeded_from_ARD*). The table lists the top ten genes prioritized by the *Multiplex Random.Walker2ARD* model, which achieved the highest predictive performance among all tested algorithms (mean accuracy=86.3%). This model integrates multiple molecular layers (PPI, $KEGG$, COX_{90}, COX_{95}) through a RWR–based diffusion process to estimate the association of candidate genes to ARDs modules. Rank: Position of each gene in the final prioritized list, sorted by predicted probability of association with ARCs. Gene: Official *HUGO* gene symbol of the predicted candidate. Prob: Model-derived probability (0–1) reflecting the confidence that a gene is mechanistically linked to ageing-related disease modules. High *ARC-Pleiotropy*: Number *ARCs* to which the gene shows direct genetic or functional association; higher values indicate broader disease involvement. *ARC-Interactor* (PPI / $KEGG$): Number of network layers (PPI and $KEGG$) in which the gene physically or functionally interacts with ARC-associated genes; larger values indicate stronger multilayer connectivity and diffusion overlap.

Rank	Gen	Prob	*ARC-Pleiotropy*	*ARC-Interactor* PPI	*ARC-Interactor* $KEGG$
1	*SMAD2*	0.982	0	4	3
2	*SMAD4*	0.976	0	2	3
3	*CSNK2A1*	0.974	0	4	2
4	*HSP90AB1*	0.974	0	5	1
5	*MAPK1*	0.974	0	3	5
6	*CDKN1B*	0.972	0	4	3
7	*PIK3R2*	0.97	0	2	5
8	*PRKCB*	0.97	0	2	4
9	*SMAD3*	0.97	0	5	3
10	*MET*	0.966	0	2	3

Supplementary Table 8. Gene Ontology enrichment for the top 30 genes predicted by the *Multiplex RWR_seeded_from_ARD* model. The table summarizes the top significantly enriched Biological Process (GO:BP) terms identified among the 30 highest-ranking genes predicted by the *Multiplex Random.Walker2ARD* model. Enrichment analyses were performed using the *clusterProfiler* R package (Xu *et al.*, 2024), with gene annotations from *org.Hs.eg.db* (Carlson, 2023) and redundancy reduction of GO terms based on semantic similarity using *GOSemSim* (Huber *et al.*, 2015). Analyses were restricted to GO:BP terms, applying a *p*-value cutoff of 0.05, a minimum gene set size of 15, and a similarity threshold of 0.8 for collapsing redundant categories. All enrichments were tested against custom background gene sets, comprising genes present across the four evaluated networks (PPI, $KEGG$, COX_{90}, COX_{95}). Columns indicate the Term name, GO identifier, adjusted *p*-value (FDR-corrected), term size (number of background genes annotated to the term), query size (30 predicted genes), and intersection size (overlapping genes per term).

Term Name	Term id	Adjusted p_value	Term Size	Query Size	Intersection Size
intracellular signal transduction	GO:0035556	7.86E-14	2673	30	25
regulation of response to stimulus	GO:0048583	8.81E-14	4131	30	28
cellular response to chemical stimulus	GO:0070887	8.09E-13	3116	30	25
positive regulation of nitrogen compound metabolic process	GO:0051173	1.02E-12	3175	30	25
response to endogenous stimulus	GO:0009719	2.35E-12	1637	30	20
regulation of cell communication	GO:0010646	5.45E-12	3464	30	25
regulation of signaling	GO:0023051	5.45E-12	3477	30	25
regulation of signal transduction	GO:0009966	5.45E-12	3075	30	24
response to chemical	GO:0042221	5.45E-12	4494	30	27
positive regulation of cellular metabolic process	GO:0031325	7.12E-12	3133	30	24
positive regulation of macromolecule metabolic process	GO:0010604	7.12E-12	3570	30	25
cellular response to organic substance	GO:0071310	1.07E-11	2464	30	22
positive regulation of metabolic process	GO:0009893	4.96E-11	3897	30	25
response to organic substance	GO:0010033	7.80E-11	3109	30	23
protein phosphorylation	GO:0006468	1.52E-10	1578	30	18
phosphorylation	GO:0016310	1.52E-10	1853	30	19
cellular response to endogenous stimulus	GO:0071495	2.72E-10	1387	30	17
regulation of developmental process	GO:0050793	3.10E-10	2584	30	21
programmed cell death	GO:0012501	5.06E-10	2000	30	19

Supplementary References

Dönertaş HM, Fabian DK, Valenzuela MF, Partridge L, Thornton JM (2021) Common genetic associations between age-related diseases. *Nat Aging* 1:400–412. https://doi.org/10.1038/s43587-021-00051-5

Lemaître, G., Nogueira, F., & Aridas, C. K. Imbalanced-learn: A Python Toolbox to Tackle the Curse of Imbalanced Datasets in Machine Learning. J. *Mach. Learn. Res.*, 18(17), 1-5 (2017).

Valdeolivas A, Tichit L, Navarro C, Perrin S, Odelin G, Levy N, Cau P, Remy E, Baudot A (2019) Random walk with restart on multiplex and heterogeneous biological networks. *Bioinformatics* **35**(3):497–505. https://doi.org/10.1093/bioinformatics/bty637

Weighill D, Jones P, Bleker C, Ranjan P, Shah M, Zhao N, Martin M, DiFazio S, Macaya-Sanz D, Schmutz J, Sreedasyam A, Tschaplinski T, et al (2019) Multi-Phenotype Association Decomposition: Unraveling Complex Gene-Phenotype Relationships. *Front Genet* 10:417. https://doi.org/10.3389/fgene.2019.00417
